# First Insights into the Diverse Human Archaeome: Specific Detection of Archaea in the Gastrointestinal Tract, Lung, and Nose and on Skin

**DOI:** 10.1128/mBio.00824-17

**Published:** 2017-11-14

**Authors:** Kaisa Koskinen, Manuela R. Pausan, Alexandra K. Perras, Michael Beck, Corinna Bang, Maximilian Mora, Anke Schilhabel, Ruth Schmitz, Christine Moissl-Eichinger

**Affiliations:** a Medical University of Graz, Graz, Austria; b BioTechMed-Graz, Graz, Austria; c University of Regensburg, Regensburg, Germany; d Christian-Albrechts-Universität zu Kiel, Kiel, Germany; University of Vienna; Pacific Northwest National Laboratory

**Keywords:** archaeome, methanogens, microbiome, Archaea

## Abstract

Human-associated archaea remain understudied in the field of microbiome research, although in particular methanogenic archaea were found to be regular commensals of the human gut, where they represent keystone species in metabolic processes. Knowledge on the abundance and diversity of human-associated archaea is extremely limited, and little is known about their function(s), their overall role in human health, or their association with parts of the human body other than the gastrointestinal tract and oral cavity. Currently, methodological issues impede the full assessment of the human archaeome, as bacteria-targeting protocols are unsuitable for characterization of the full spectrum of *Archaea*. The goal of this study was to establish conservative protocols based on specifically archaea-targeting, PCR-based methods to retrieve first insights into the archaeomes of the human gastrointestinal tract, lung, nose, and skin. Detection of *Archaea* was highly dependent on primer selection and the sequence processing pipeline used. Our results enabled us to retrieve a novel picture of the human archaeome, as we found for the first time *Methanobacterium* and *Woesearchaeota* (DPANN superphylum) to be associated with the human gastrointestinal tract and the human lung, respectively. Similar to bacteria, human-associated archaeal communities were found to group biogeographically, forming (i) the thaumarchaeal skin landscape, (ii) the (methano)euryarchaeal gastrointestinal tract, (iii) a mixed skin-gastrointestinal tract landscape for the nose, and (iv) a woesearchaeal lung landscape. On the basis of the protocols we used, we were able to detect unexpectedly high diversity of archaea associated with different body parts.

## IMPORTANCE

In summary, our study highlights the importance of the primers and NGS data processing pipeline used to study the human archaeome. We were able to establish protocols that revealed the presence of previously undetected *Archaea* in all of the tissue samples investigated and to detect biogeographic patterns of the human archaeome in the gastrointestinal tract, on the skin, and for the first time in the respiratory tract, i.e., the nose and lungs. Our results are a solid basis for further investigation of the human archaeome and, in the long term, discovery of the potential role of archaea in human health and disease.

## INTRODUCTION

Our invisible organ, the human microbiome, is composed of *Bacteria*, eukaryotes, *Archaea*, and viruses. As *Bacteria* are considered to be the predominant component of the human microbiome, the overwhelming majority of studies focus on their contribution (1). Recently, research has been directed to the eukaryome, mycobiome (2–4), and virome (5). However, the human archaeome remains largely uninvestigated, although *Archaea* are known to strongly impact human health and well-being (6, 7). In particular, methanoarchaea (methane-producing archaea, methanogens) are believed to be keystone species that may have a greater influence on the composition and function of the whole gastrointestinal microbial community than we currently know (8–10).

To date, four species of methanogenic archaea have been cultivated and isolated from human body samples: the first isolated and described human-associated archaeon *Methanobrevibacter smithii* (11), *Methanosphaera stadtmanae* (12), and most recently *Methanomassiliicoccus luminyensis* (13) were all isolated from human feces. A fourth isolate, *Methanobrevibacter oralis* (14) was cultivated and described from oral mucosa. In recent years, with the help of molecular methods, several other archaeal groups have been found in the human body. For example, two candidate species, “*Candidatus* Methanomassiliicoccus intestinalis” and “*Candidatus* Methanomethylophilus alvus,” as well as several unknown members of the orders *Methanosarcinales*, *Methanobacteriales*, *Methanococcales*, *Methanomicrobiales*, and *Methanopyrales*, have been found to inhabit the human gut (15). It is estimated that up to 96% of all people carry *M. smithii* in their guts (16, 17). The abundance of this archaeon in the human gut differs in individual human subjects, but it can account up to 10% of the anaerobic microbes in the colon (18). In addition, *M. stadtmanae* was reported to be highly abundant and was detected in about 30% of the subjects tested (16). Together with the recently discovered species *M. luminyensis*, with a prevalence of 4% in the population studied (17), *M. smithii* and *M. stadtmanae* are currently known to predominantly inhabit the human gut.

In natural environments, most methane-producing archaea use C_1_ and C_2_ carbon sources and hydrogen for methane production in the last step of the anaerobic food chain. The constant removal of molecular hydrogen drives preceding bacterial metabolic processes (primary and secondary fermenters), as higher hydrogen partial pressure turns these activities thermodynamically unfavorable (19). On the other hand, methanogenic archaea have been reported to be associated with different disease patterns, such as the development of inflammatory bowel disease (20), dental disease (21, 22), or potentially even brain abscesses (23).

However, not all human gut-associated archaea are methanogens; unknown members of the orders *Desulfurococcales*, *Sulfolobales*, *Thermoproteales*, *Nitrososphaerales*, and *Halobacteriales* have also been detected in the human intestine (15). Recently, a new species of halophilic archaea, *Haloferax massiliensis* (24), has been isolated from the human gut, again raising the question of whether halophilic archaea could potentially be permanent residents (25). Besides the gut, *Archaea* have also been reported to be widely distributed in other human body sites, such as the vaginal cavity (*M. smithii*) and skin (*Thaumarchaeota*) (26–28). Although their role is largely unknown, skin-associated *Thaumarchaeota* can make up as much as 10% of the microbiome, in particular in skin samples from elderly persons and children (29).

Research on microbiomes and on the human microbiome in particular was pushed forward years ago by the application of next-generation sequencing (NGS) in amplicon-based studies. Since then, the search for optimal methods in preparatory laboratory work, subsequent primer selection, and PCR amplification has begun. The ability to create more data in less time also increased the need to optimize data processing workflows and displaying methods after sequencing. Although the results of human microbiome research are exciting, a certain incomparability between microbiome studies that have used various methods on different levels has recently been observed. The influence of the chosen methodology was recognized as the major driving force behind the dissimilarity of results, as shown in one example of indoor microbiome studies (30). On the basis of these observations, standardized procedures have been proposed by world-leading projects such as the Human Microbiome Project (HMP) (1) and the Earth Microbiome Project (EMP) (31) to simplify comparisons between different studies. In the HMP, the focus is on the bacteriome (the diversity of bacteria), with standard operating procedures (SOPs) proposing the use of so-called “universal,” but mainly bacteria-targeting, primers for the 16S rRNA gene amplicon sequencing approach. Consequently, the diversity and abundance of archaea are mostly inferred from “side products” of studies targeting bacteria (27, 32). As a consequence, archaeal 16S rRNA gene information is frequently filtered from the data set during sequence processing (33). Such problems are also reflected in metagenomic analyses, in which assignable archaeal reads typically represent a small minority as well (34). The specific detection of archaeal signatures suffers from methodological problems on various levels, including DNA extraction, selection of suitable primers, a low abundance of archaeal DNA, or incompleteness of 16S rRNA gene reference databases (for more detailed information on these issues, see Text S1 in the supplemental material; for a summary of the targeted archaeal groups and genes, sample types, molecular methods, and primers that have been used to detect *Archaea* in human-associated samples, see Table S1).

10.1128/mBio.00824-17.1 TEXT S1  Additional information on the introduction, Materials and Methods, and Discussion. Download TEXT S1, DOCX file, 0.2 MB. Copyright © 2017 Koskinen et al. 2017 Koskinen et al. This content is distributed under the terms of the Creative Commons Attribution 4.0 International license.

10.1128/mBio.00824-17.8 TABLE S1  Summary of the targeted archaeal groups and genes, sample types, molecular methods, and primers used to detect *Archaea* in human samples (literature overview). Download TABLE S1, XLSX file, 0.03 MB. Copyright © 2017 Koskinen et al. 2017 Koskinen et al. This content is distributed under the terms of the Creative Commons Attribution 4.0 International license.

Besides those methodological problems, archaeome research faces another challenge; with no known archaeal pathogen, these species are mostly ignored and the need for a detailed analysis is not recognized within the big and well-funded field of clinical microbiology (35, 36).

In this study, we are taking the first step in a long row of necessary improvements for the specific detection of archaea in large studies. Here, we focused on optimal primer selection and analyzed the ability of available NGS sequence processing pipelines, namely mothur (37), QIIME (Quantitative Insights Into Microbial Ecology) (38), and DADA2 (Divisive Amplicon Denoising Algorithm) (39), to expand the picture of the human archaeome.

More specifically, we present two main approaches. We first used a PCR-based detection method to determine the presence or absence of the well-known human-associated methanoarchaea in a variety of biopsy samples from the gut, providing a sound basis for determining the involvement of these archaea in inflammatory processes in the human gut.

The second approach addresses the need for archaea-specific NGS-based diversity analyses. We critically assessed the capacity of archaeal and “universal” primers to detect archaea-specific signals via NGS in different human tissue samples and processed data with different NGS pipelines. As a proof of principle, we applied our most specific and conservative protocol to a variety of human tissue samples, including skin samples, lung samples, biopsy specimens from the gastrointestinal tract (GIT), stool samples, and nasal samples, uncovering a previously unknown diversity of archaea associated with specific sites of the human body.

## RESULTS AND DISCUSSION

Human-associated archaea remain, similar to fungi or viruses, understudied in the field of microbiome research. Numerous studies have indicated the potential of human-associated archaea (in particular, methanogens) to represent keystone species, as they can be major drivers of metabolic processes in the gut. Recent studies have shown that the human archaeome still holds many surprises, including the discovery of the seventh order of methanogens in the human gut (13), the cultivation of a halophilic archaeon from human samples (24), and the detection of *Thaumarchaeota* on human skin (27). Unfortunately, studies on the human archaeome are rare, mainly due to methodological issues and a lack of motivation from a medical point of view because no firm evidence of an archaeal pathogen has been discovered. In this study, we focused on primer choice and sequence processing protocols to optimize specific detection of (methano)archaea in human samples. The most conservative and most specific protocol was then used to determine the diversity of archaea associated with human skin, lung, GIT, stool, and nasal samples.

### A PCR-based methanoarchaea-targeting approach allows the specific detection of human-associated methanogens (*M. smithii*, *M. stadtmanae*, and *M. luminyensis*) in healthy and inflamed gut biopsy samples (approach 1).

In the first approach tested in this study, we concentrated on facilitated, NGS-independent detection of the three most abundant (identified) methanoarchaea of the human gut microbiome. With this setup, we addressed the question of whether there is a pattern of co-occurrence of various mucosa-associated methanoarchaeal strains with the manifestation of inflammatory processes involving the human GIT.

Biopsy samples retrieved from healthy controls and diseased persons (Crohn’s disease [CD], ulcerative colitis [UC]) at both inflamed and uninflamed locations were analyzed for the presence of *M. smithii*, *M. stadtmanae*, and *M. luminyensis*. Primers were selected and designed on the basis of a previously described quantitative reverse transcription-PCR setup for quantifying *M. smithii* and *M. stadtmanae rpoB* genes (16). Primers for the detection of *M. luminyensis* targeted the 16S rRNA gene and were specifically designed in this study (see Materials and Methods). In all cases, the evaluation demonstrated that a nested PCR was required, since a single PCR with strain-specific primers never provided immediate positive results for *M. luminyensis* and *M. stadtmanae* and only in rare cases for *M. smithii*. The results of the second PCR are shown in Table 1. All nested PCRs were performed in triplicates if not mentioned otherwise.

**TABLE 1:** Results obtained for the species-specific detection of *Methanoarchaea* in gut biopsy samples of healthy controls and UC and CD patients^a^

Target or parameter	Healthy controls					Inflamed UC					Uninflamed UC					Inflamed CD					Uninflamed CD				
	1^b^	2	3	4	5	1	2	3	4	5	1	2	3	4	5	1	2	3	4	5	1	2	3	4	5
*M. smithii*	−/+/+	−	+/+/+	−	−	−/+/−	+/+/+	+/+/−	+/−/−	+/+/−	−/+/−	+/−/−	−/+/−	−/−/−	−/+/−	+/+/−	+/−/+	+/+/−	+/−/−	−/+/+	+/−/−	+/+/−	−/+/−	−/+/−	−/+/−
*M. stadtmanae*	−/+/−	−/−	−/+/−	−/−	−/+	−/−/−	−/−/+	−/−/−	−/−/−	−/−/−	+/−/−	−/+/+	−/−/−	−/−/−	−/−/−	−/−/−	+/−/−	−/−/−	−/−/−	−/+/−	−/−/−	−/+/−	−/−/−	−/−/−	−/−/−
*M. luminyensis*	−/−/−	−/−/−	+/−/−	−/−/−	+/−/−	−/−/−	−/−/−	−/−/−	−/+/−	−/−/−	−/−/−	−/−/−	−/−/−	−/−/−	−/−/−	−/−/−	+/+/−	+/−/−	+/−/−	−/−/−	−/−/−	−/−/−	−/−/−	−/−/−	−/−/−
*Archaea^c^*	−/−/−	−/+/−	+/+/+	−/−/−	+/−/+	+/+/+	−/+/−	−/−/−	−/−/+	−/−/−	−/−/−	−/+/+	−/+/+	−/−/−	−/+/+	−/+/−	−/+/−	−/+/−	−/−/−	−/−/+	−/−/+	+/+/+	+/+/−	−/+/−	−/−/−
Identity^d^		*M. smithii*	*M. smithii*, *M. stadtmanae*		Euryarchaeote SCGC AAA003-G23	*M. smithii*	*M. smithii*, *M. stadtmanae*		*M. stadtmanae*			*M. smithii*	*M. smithii*, *M. stadtmanae*		*M. smithii*	*M. smithii*	*M. smithii*, *M. stadtmanae*	*M. smithii*		*M. stadtmanae*	*M. smithii*, *M. stadtmanae*	*M. smithii*	*M. stadtmanae*	*M. smithii*	

^a^ Nested PCR of the *rpoB* gene (and of the 16S rRNA gene as a control) was performed as described in Materials and Methods. Positive (+) and negative (−) results out of three replicates are shown (each symbol reflects one replicate). Where only one or two symbols are shown, three replicates could not be analyzed because of a low DNA yield.

^b^ Biopsy sample number.

^c^ 16S rRNA gene.

^d^ Obtained by Sanger sequencing.

Overall, signatures of *M. smithii* were found to be the most abundant, as 10 of 25 samples (at least two positive results out of three replicates) or 21 of 25 samples (at least one positive result) revealed positive amplification. *M. stadtmanae* was found in 1 of 25 samples (at least two positive results out of three replicates) or in 8 of 25 samples (at least one positive result). These findings were in congruence with the archaea-targeting results (see below). *M. luminyensis* signatures were detected in 1 of 25 samples (at least two positive results out of three replicates) or 7 of 25 samples (at least one positive result out of three replicates; Table 1). For verification, all samples were also subjected to 16S rRNA gene amplification (also by a nested PCR approach) targeting archaea in general (Table 1; Text S1; Fig. S1). With this experimental setup, the presence of archaeal signatures in 9 of 25 samples (at least two positive results out of three replicates) or 17 of 25 samples (at least one positive result out of three replicates) could be shown. Sanger sequencing of these PCR products identified mainly gene signatures of *M. smithii* (12 samples), as well as *M. stadtmanae* (8 samples), whereas signatures of *M. luminyensis* were not confirmed. Although the results basically overlapped, several samples that were negative in the archaea-targeting PCR were positive when a species-specific nested PCR assay was performed (Table 1).

10.1128/mBio.00824-17.2 FIG S1  Nested PCR results obtained with primer pairs 342F/1204R and 571F/976R. Primer pair 342F/1204R was used to detect the 16S rRNA gene in 100 ng of template DNA from 25 biopsy samples (A). Subsequently, 2 µl of the PCR product from panel A was used in a nested PCR with primer pair 571F/976R (B). One hundred picograms of archaeal DNA served as a positive control, and 100 pg of bacterial DNA and sterile double-distilled water served as negative controls. Download FIG S1, TIF file, 0.3 MB. Copyright © 2017 Koskinen et al. 2017 Koskinen et al. This content is distributed under the terms of the Creative Commons Attribution 4.0 International license.

In general, the nested PCR approach proposed was found to be suitable for the detection of specific strains in human biopsy specimens. An advantage of the strain-specific detection method described is proof of the presence of known strains despite their comparatively low abundance. However, the overall results of this method were not always clear, probably because of the comparatively small proportion of archaeal DNA (40, 41), and no obvious pattern was obtained regarding the presence of methanoarchaeal strains in patients with inflamed or uninflamed UC or CD. Information needs to be obtained from quantitative studies and larger cohorts to resolve this question. One reason for the inconsistency of PCR-based results might be the presence of a large amount of eukaryotic DNA in the purified DNA fractions. Thus, determination of archaeal strains in samples containing large amounts of eukaryotic DNA, such as biopsy specimens, might be made more consistent by using, e.g., NGS-based analyses. This will be addressed later. It is noteworthy that the proven presence of methanoarchaea in biopsy samples suggests that these microorganisms most likely are associated with the mucosa and are not only present in the lumen of the GIT. Previous studies have hypothesized the attachment of these strains to the intestinal epithelia because of their ability to form biofilms on nonliving surfaces (42). This hypothesis is supported by genomic studies of *M. smithii* and *M. stadtmanae* that showed that both species express adhesion-enabling proteins similar to bacterial adhesins (18).

### Primer pairs and sequence data processing pipelines determine the detection of archaeal operational taxonomic units (OTUs) and ribosomal sequence variants (RSVs) in natural mock communities (approach 2).

In a preliminary step, we tested the efficiency of three different primer pairs suitable for NGS (short read length), namely, 515f/806r targeting *Bacteria* and *Archaea* (43), 349af/519ar (344f/915r) targeting *Archaea*, and 519af/785ur (344f/915r) targeting *Bacteria* and *Archaea* (44), with respect to their (specific) ability to detect the diverse archaea in human stool samples, serving as natural mock communities. We used two human stool samples from healthy persons, as this type of sample is often used for microbiome analyses and as archaea have been successfully detected in human stool samples in the past (16).

The first primer pair we tested was the commonly used “universal” primer pair 515f/806r, targeting both *Bacteria* and *Archaea* (43) (proposed by the HMP and the EMP). The other two primer pairs were used in a nested PCR strategy with an archaea-specific first PCR. For the first PCR, we used the primer pair 344f/915r (27, 45, 46). The nested PCR method was used to (i) preselect archaeal fragments, (ii) increase the amount of archaeal signatures, and (iii) avoid the formation of primer dimers, which were observed when primers tagged with Illumina adapter and barcode were used without previous amplification.

After the initial PCR with the well-established primer pair 344f/915r, which is very selective for the archaeal domain, either primer combination 349af/519ar (coverage without a mismatch, 78.7% of *Archaea* and 0% of *Bacteria* on the basis of *in silico* analysis), targeting *Archaea*, or primer combination 519af/785ur (coverage without a mismatch, 88.7% of *Archaea* and 88.5% of *Bacteria*), targeting both *Bacteria* and *Archaea* (44), were used in a subsequent PCR approach (see Materials and Methods for details). All three protocols allowed the successful amplification of 16S rRNA gene fragments, which were sequenced by Illumina MiSeq. The raw sequence data set retrieved was then subjected to three different analysis pipelines based on QIIME, mothur, and DADA2 (37–39) to assess the impact of the data processing pipeline used on the results. All three pipelines were used in accordance with the SOPs proposed by their developers.

The different primer combinations revealed diverse levels of specificity with respect to *Archaea* detection (Table 2) (detailed results [OTU and RSV tables] are available upon request). The primer pair 349af/519ar (following the first PCR with 344f/915r) allowed the retrieval of solely archaeal sequences and was, as expected, found to be the most specific approach. With all of the data processing pipelines used, the percentage of archaeal reads was 100%. However, the number of archaeal OTUs/RSVs observed was significantly less than that obtained with the 519af/785ur primer combination when the sequence data were processed with mothur (*t* test, *P* = 0.00007) or DADA2 (*t* test, *P* = 0.002). Conversely, a different picture was obtained from the QIIME processing pipeline, as the same data sets resulted in a higher number of archaeal OTUs from the more specific primer pair 349af/519ar (344f/915r) than from the combination 519af/785ur (344f/915r) (*t* test, *P* = 0.00004).

**TABLE 2:** Summary of the bioinformatics pipelines and primer combinations used and archaeal and bacterial OTUs/RSVs detected in the stool samples studied^a^

Primers	QIIME					Mothur					DADA2				
	Archaeal OTUs		Bacterial OTUs		% of archaeal OTUs	Archaeal OTUs		Bacterial OTUs		% of archaeal OTUs	Archaeal OTUs		Bacterial OTUs		% of archaeal OTUs
	Sample 1	Sample 2	Sample 1	Sample 2		Sample 1	Sample 2	Sample 1	Sample 2		Sample 1	Sample 2	Sample 1	Sample 2	
349af/519ar (344f/915r)	484 ± 80^b^	622 ± 27	0 ± 0.5	0 ± 0	100	320 ± 70	411 ± 31	0 ± 0	0 ± 0	100	8 ± 2	15 ± 5	0 ± 0	0 ± 0	100
519af/785ur (344f/915r)	36 ± 2.5	37 ± 2	216 ± 90	84 ± 44	16–44	8,990 ± 2,030	7,869 ± 1,182	146 ± 45	83 ± 40	98–99	63 ± 22	52 ± 13	69 ± 27	21 ± 19	48–71
515f/806r	0 ± 0	1 ± 0.5	4,873 ± 1,146	6,618 ± 874	0–0.02	1 ± 0	1 ± 0	20,648 ± 6,208	25,063 ± 7,033	0.004–0.005	0 ± 1	1 ± 1	166 ± 24	274 ± 43	0–0.36

^a^ The primer pair in parentheses (344f/915r) was used in the first round of a nested PCR. The samples were amplified in three replicates. The same sequence data sets were used for the different pipelines.

^b^ Median ± interquartile range.

Primer pair 519af/785ur (344f/915r) was capable of retrieving the broadest diversity of archaea, but because of the different primer specificities, a vast variety of bacterial signatures were also obtained. The percentage of OTUs classified as archaea and the number of archaeal OTUs/RSVs detected depended largely on the data processing pipeline used (one-way analysis of variance [ANOVA] *P* = 1,09E-10) (Table 2). Additionally, our results show that the universal and widely used primer pair 515f/806r failed to detect the diverse archaea in stool samples. By this approach, all of the data processing pipelines tested were able to capture only one archaeal OTU per sample, corresponding to 0 to 0.36% of the OTUs and RSVs retrieved (Table 2, last column).

On the basis of our analyses, primer pair 349af/519ar proved superior to the other primer pairs tested with respect to archaeal specificity and thus was used later in this study to analyze human tissue samples. All of the different pipelines reflected a similar picture of the results, although the mothur pipeline tended to overestimate the number of OTUs. DADA2 allowed the retrieval of the most conservative data set, with a lower number of RSVs than mothur and QIIME. In principle, and as also shown by *in silico* analysis, primer pair 349af/519ar could even be used for NGS amplicon studies without a nested approach with a first PCR with 344f/915r, as the amplicon has an appropriate length and the overall coverage for archaea would allow the identification of a wide taxonomic diversity (44). However, our methodology required the addition of Illumina adapter sequences to the primers, which resulted in severe primer dimer formation during a single-step PCR, making this approach ineffective in this experimental study (for further details, see Text S1).

### The combination of primer pair 349af/519ar (344f/915r) and the DADA2 pipeline allows for the most conservative detection of archaeal signatures in human samples.

Our NGS approach was based on the use of the Illumina MiSeq platform to study the community composition and diversity of archaea in human tissue samples. In proof-of-principle experiments, we assessed the efficiency of one primer pair, namely, 349af/519ar (344f/915r) (selected on the basis of its clear archaeal specificity and promising results in the natural mock community experiment), to analyze the diversity of archaea in various human microbiome samples, including gut biopsy, lung (bronchoalveolar lavage [BAL] fluid), olfactory mucosa (nasal) samples, and skin wipe samples from the forearm, chest, and back. As the Illumina data error profiles have not been well understood, and the widely used publicly available sequence data analysis pipelines were originally not designed for the characteristics of Illumina data (47), we were particularly interested in the impact of the sequence processing platforms chosen, namely, QIIME, mothur, and DADA2, on the results acquired (for further details on QIIME and mothur, see Text S1). DADA2, published in 2016, is an open-source software package designed to model and correct Illumina-sequenced amplicon errors in particular. It has been demonstrated to identify more variants and produce fewer spurious sequence reads than other sequence processing tools. DADA2 does not have a clustering step, and it is a full amplicon workflow with steps including filtering, dereplication, sample inference, chimera identification, and merging of paired-end reads (39). The different principle of DADA2 was clearly reflected in our results, as DADA2 retrieved approximately 200 bacterial RSVs from the unspecific approach with primer pair 515f/806r, whereas mothur detected more than 20,000 OTUs in the same sequence data sets. The same trend was observed with archaeal OTUs and RSVs, respectively, as we obtained 911 archaeal OTUs with QIIME, 999 OTUs with mothur, and 50 RSVs with DADA2 (for full details, see Table S2.)

10.1128/mBio.00824-17.9 TABLE S2  OTUs and RSVs of samples from different body sites. Download TABLE S2, XLSX file, 1.5 MB. Copyright © 2017 Koskinen et al. 2017 Koskinen et al. This content is distributed under the terms of the Creative Commons Attribution 4.0 International license.

When analyzing the stool samples that served as natural mock communities, the most conservative, and thus most stringent, approach was found to be the combination of primer pair 349af/519ar (344f/915r) amplicons with data processing through DADA2. Here, DADA2 also retrieved the most conservative estimation of archaeal diversity as estimates based on the number of RSVs but also reflected the broadest diversity based on the number of taxa identified. Consequently, in the following, we will focus on the results retrieved from the DADA2 pipeline. However, additional results of pipeline comparisons are fully described in Text S1.

### First insights into the archaeome of the human GIT, skin, nose, and lung reveal unexpected diversity and novel human archaeome signatures.

**FIG 1:**
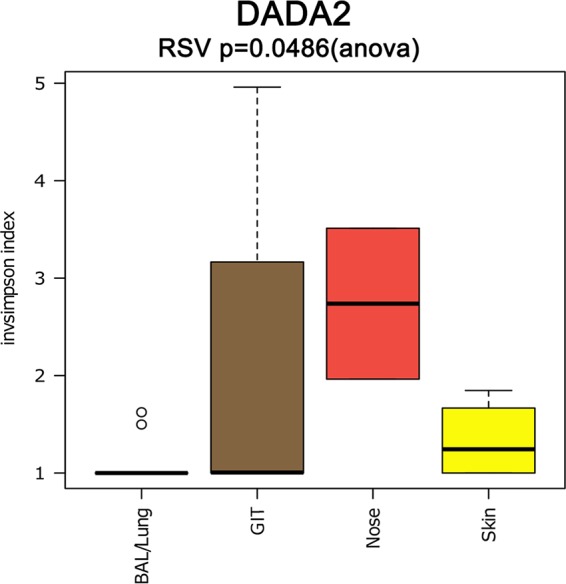
Alpha diversity based on the inverse Simpson diversity index in BAL fluid/lung (blue), GIT (brown), nasal (red), and skin (yellow) samples.

On the basis of the above-described conservative NGS protocol, the nasal samples carried the highest diversity of archaea, followed by skin and gut samples (Fig. 1; Text S1; Fig. S2A), whereas the lowest diversity was retrieved from lung samples. The difference in alpha diversity between the body sites was found to be significant (*P* = 0.0486; DADA2 output; Fig. 1).

10.1128/mBio.00824-17.3 FIG S2  (A) Alpha diversity based on the inverse Simpson diversity index in BAL fluid/lung (blue), GIT (brown), nasal (red), and skin (yellow) samples; results obtained with the three different pipelines are shown. (B) RDA plots for samples from different body sites, i.e., BAL fluid/lung (blue circles), gut (brown squares), nose (red diamonds), and skin (yellow triangles). Download FIG S2, TIF file, 0.1 MB. Copyright © 2017 Koskinen et al. 2017 Koskinen et al. This content is distributed under the terms of the Creative Commons Attribution 4.0 International license.

In total, signatures from five archaeal phyla (*Euryarchaeota*, *Thaumarchaeaota*, *Diapherotrites*, *Woesearchaeota*, *Aenigmarchaeota*; the last three phyla are associated with the DPANN [*Diapherotrites*, *Parvarchaeota*, *Aenigmarchaeota*, *Nanoarchaeota*, *Nanohaloarchaea*] superphylum) (48) were identified in the human samples, along with numerous unclassified sequences (Table S2; more information is shown below).

Besides the finding that not only the skin (27) and gut, but also the nose and lung harbor a unique archaeal community, we were most surprised by the retrieval of *Methanobacterium* signatures from the human gut (see below) and the detection of a broad variety of DPANN superphylum-associated sequences from diverse human samples. In particular, members of *Woesearchaeota* were identified as the dominating archaeal taxon in the human lung. Signatures from the DPANN superphylum have never been associated with the human microbiome before. However, the presence of *Woesearchaeota* signatures has been reported in human-associated areas such as door handles (49) and in dust on the International Space Station (50), as well as in other environmental samples (49, 51, 52). Information on this clade of archaea is sparse, except for speculations on a potential parasitic/symbiotic lifestyle based on the observation of a small, reduced genome (53). Very recent studies suggest a close relationship to *Eukarya* (54), but their role in the environment is still unclear.

Besides methanogens, haloarchaea have also been reported to be coinhabitants of the colon and potentially also of the skin (24, 27, 55). Notably, haloarchaeal taxa were not detected in any of the tissue samples in our study. Since the primers used were found to be able to also target haloarchaea *in silico*, our results indicate an absence of this archaeal clade in our samples.

### Archaea-specific amplification, NGS, and sequence processing procedures allow novel insights into the composition and distribution of the human GIT archaeome.

On the basis of the data sets retrieved, we further explored the archaeal communities associated with different parts of the human GIT. As stool samples do not allow a deeper insight into the mucosa-associated microbiota, we selected biopsy specimens from seven different GIT locations, the corpus, antrum, duodenum, ileum, appendix, right colon, and left colon, for this study.

To explore possible differences between the archaeal GIT communities, alpha diversity (inverse Simpson index) was assessed and multivariate analyses were performed (Text S1; Fig. S3A). Statistical analyses did not show a significant (*P* > 0.05) influence of the sample location within the GIT on the diversity of archaea and community composition. Redundancy analysis (RDA), however, indicated a potential separate clustering of left colon and ileum communities (Text S1; Fig. S3B).

10.1128/mBio.00824-17.4 FIG S3  (A) Alpha diversity analysis (inverse Simpson index) of the archaeal communities retrieved from biopsy specimens from various locations along the human GIT with all of the pipelines used in this study. (B) RDA plots for the antrum, appendix, corpus, duodenum, ileum, left (l.) colon, and right (r.) colon obtained with all of the pipelines used in this study. Download FIG S3, TIF file, 0.2 MB. Copyright © 2017 Koskinen et al. 2017 Koskinen et al. This content is distributed under the terms of the Creative Commons Attribution 4.0 International license.

**FIG 2:**
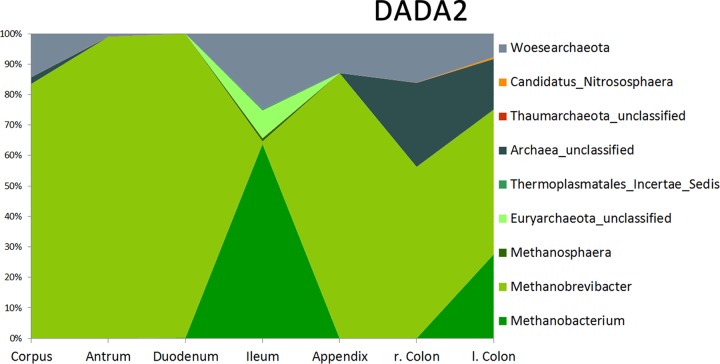
Area plot of the relative abundance (percent) of archaeal signatures (at the genus level) detected in GIT biopsy samples. *Methanoarchaea* are displayed in shades of green. The arrangement on the *x* axis reflects the sequential of the locations in the human GIT (from the stomach to the anus).

This was confirmed by area plots on the genus level, which also revealed a dissimilar picture in different GIT areas (Fig. 2; Text S1; Fig. S4).

10.1128/mBio.00824-17.5 FIG S4  Area plot of the relative abundance (percent) of archaeal signatures (genus level) detected in GIT biopsy samples with all of the pipelines used in this study. *Methanoarchaea* are displayed in shades of green. The sequence of locations on the *x* axis reflects the sequential arrangement of the locations in the human GIT (from the stomach to the anus). Download FIG S4, TIF file, 0.5 MB. Copyright © 2017 Koskinen et al. 2017 Koskinen et al. This content is distributed under the terms of the Creative Commons Attribution 4.0 International license.

Methanobrevibacterial signatures were detected in each location within the GIT, clearly representing the most abundant archaeal taxa therein. However, many additional sequences fell into the *Methanobrevibacter* clade but could not be classified to species level on the basis of the outcomes of the sequence processing pipelines. Taxonomic analysis (tree construction; see below), however, indicated clustering with known *Methanobrevibacter* signatures, including *M. smithii*, *M. oralis*, *M. arboriphilus*, *M. filiformis*, and *M. woesei*.

Overall, 70 to 90% of the reads from GIT samples (depending on the pipeline used) belonged to *Methanobrevibacter*, followed by *Methanobacterium* signatures (5 to 15%). More specifically, DADA2 retrieved 0.8% *Methanobrevibacter* signatures in ileum samples and 99.7% in duodenum biopsy specimens; *Methanobacterium* signatures made up 0.18% of the duodenum samples and 63.7% of the ileum biopsy specimens, indicating a prevalence of *Methanobacterium* particularly in the ileum. Signatures of the *Methanosphaera* clade were also detected in GIT samples, as well as *Methanomassiliicoccales* (“*Methanoplasmatales*”/*Thermoplasmatales*) signatures (56).

As mentioned above, the detection of *Methanobacterium* signatures was unexpected, as the presence of this genus in the human GIT has not been reported before. *Methanobacterium* is usually found in anaerobic digesters, freshwater sediments, marshy soils, and the rumen of cattle or sheep (57). Similar to *Methanobrevibacter*, it can use H_2_ and CO_2_, as well as formate, as a substrate for methanogenesis. However, as no other study has so far reported the presence of *Methanobacterium* in human gut samples and our observations were retrieved from NGS data only, we cannot infer physiological consequences from our findings. Further, as we were able to detect *Methanobacterium* solely in biopsy samples from the ileum and left colon, one might speculate about a potential association with the gut mucosa, hindering proper detection in stool samples.

### The archaeome of the human body is site specific.

To explore possible differences between the archaeal communities of the different body sites, multivariate analyses were performed. RDA was used to visualize how the archaeal communities of the different sites (lung, gut, nose, skin) relate to the other body sites and to test if the variation can be explained by the differences between sampling sites (Text S1; Fig. S2B).

**FIG 3:**
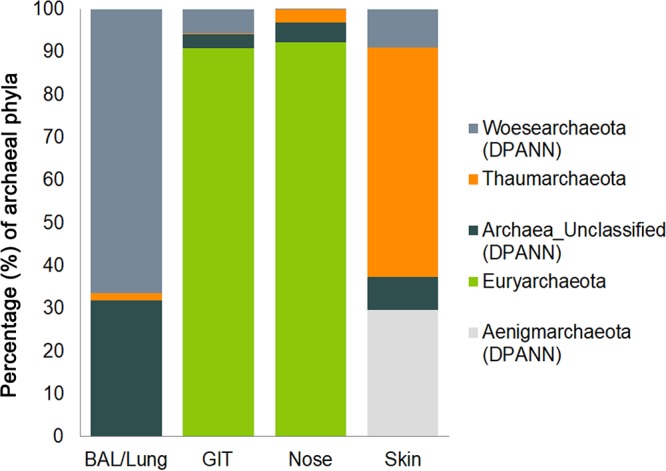
Archaeal composition at the phylum level in BAL fluid/lung, gut, nasal, and skin samples. The bar chart shows the relative abundance (percent) of the archaeal phyla in samples taken from different body sites. The different phyla are indicated by different colors, as shown on the right. DPANN archaeal signatures are shown in different shades of gray.

On the basis of our results, we were able to assign a specific archaeal community to each of the body parts analyzed; independently of the data processing pipeline used, the body location was found to be the determining factor of the microbial community’s composition (*P* < 0.05) (Fig. 3; DADA2 data set; Text S1; Fig. S5). This finding is in congruence with previous findings on bacterial communities in general (33). Costello et al. reported a predictable biogeographic bacterial pattern for body sites and even within different parts of body sites (58), which evidently is also true for archaeal communities.

10.1128/mBio.00824-17.6 FIG S5  Archaeal composition at the phylum level in BAL fluid/lung, gut, nasal, and skin samples. The bar chart shows the relative abundance (percent) of the archaeal phyla in samples taken from different body sites. The different phyla are represented by different colors, as shown on the right. DPANN archaeal signatures are shown in different shades of gray. Download FIG S5, TIF file, 0.8 MB. Copyright © 2017 Koskinen et al. 2017 Koskinen et al. This content is distributed under the terms of the Creative Commons Attribution 4.0 International license.

**FIG 4:**
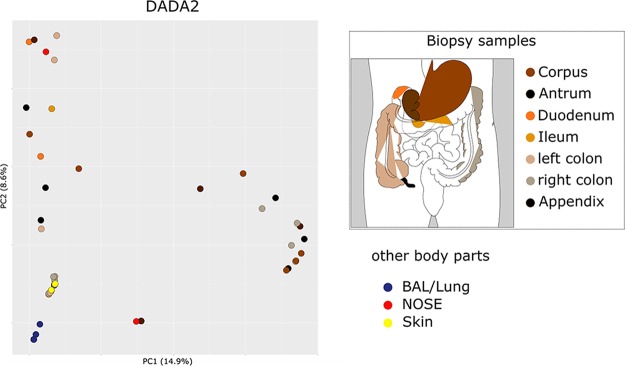
PCoA plot of the archaeal communities retrieved from human GIT, BAL fluid (lung), nasal, and skin samples. The color key explains the coloring of the samples according to their origins.

Although RDA plots revealed a grouping of nose- and skin-associated archaeal communities, the lung and gut communities did not separate clearly, indicating a certain similarity of these communities on the RSV level (Text S1; Fig. S2B). These observations were partially confirmed by principal-coordinate analysis (PCoA) (Fig. 4; Text S1; Fig. S6A).

10.1128/mBio.00824-17.7 FIG S6  (A) PCoA plots of the archaeal communities retrieved from human GIT, BAL fluid (lung), nasal, and skin samples. The color key explains the coloring of the samples according to their origins. (B) Network visualization of OTUs/RSVs retrieved with three sequence data processing pipelines. Download FIG S6, TIF file, 0.4 MB. Copyright © 2017 Koskinen et al. 2017 Koskinen et al. This content is distributed under the terms of the Creative Commons Attribution 4.0 International license.

**FIG 5:**
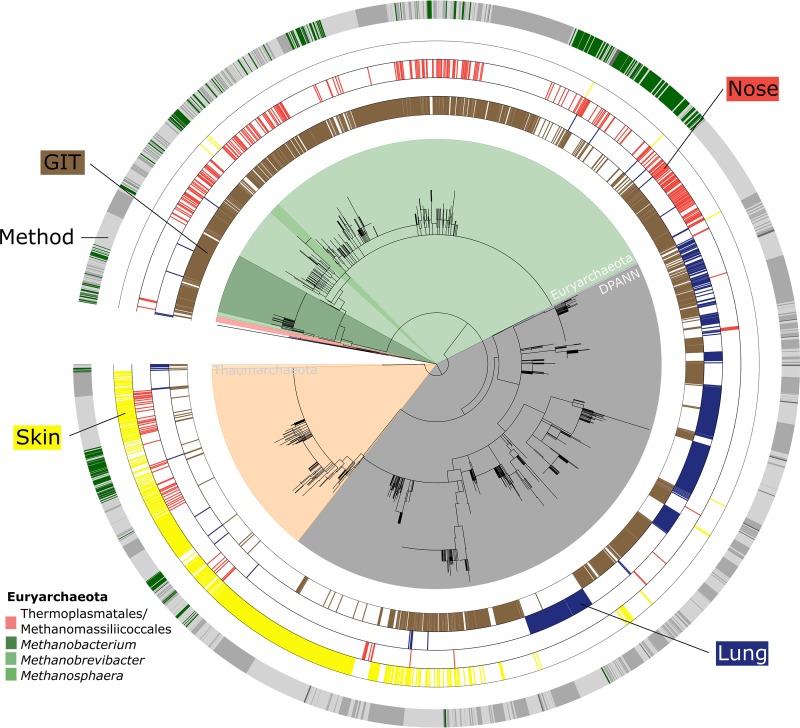
Phylogenetic tree of retrieved archaeal signatures affiliated with *Euryarchaeota*, *Thaumarchaeota*, and the DPANN superphylum. The “method” circle displays the origins of the respective OTUs/RSVs. Green represents database sequences of next neighbors as identified through SILVA SINA aligner. Dark gray represents RSV sequences (DADA2), medium gray represents OTUs retrieved through the mothur approach, and light gray represents the QIIME procedure. The four inner circles display the origins of the respective OTUs, i.e., the skin (yellow), nose (red), lung (blue), and gut (brown).

Overall, the information retrieved indicated a niche differentiation of human-associated archaeal communities; independently of the pipeline used, (anaerobic) methanogenic *Euryarchaeota* signatures were frequently detected throughout the anoxic human GIT (15) and nasal samples but were hardly found in skin or lung samples. Nasal samples harbored both methanogenic *Euryarchaeota* and skin-associated *Thaumarchaeota* signatures, indicating an overlap of the niche properties of the skin and digestive tract. The predominance of thaumarchaeal signatures on oxygenated, ammonia/urea-rich human skin (27, 29) was confirmed, and DPANN-associated archaea (*Woesearchaeota* and unassigned archaea) were detected specifically in samples from the lung (Fig. 5).

On the basis of these findings, the archaeal biogeographic pattern can be divided into (i) a thaumarchaeal skin landscape, (ii) a (methano)euryarchaeal GIT landscape, (iii) a mixed skin-GIT landscape for the nose, and (iv) a woesearchaeal lung landscape.

### Network analyses reveal the connection of the archaeal communities on the OTU/RSV level.

**FIG 6:**
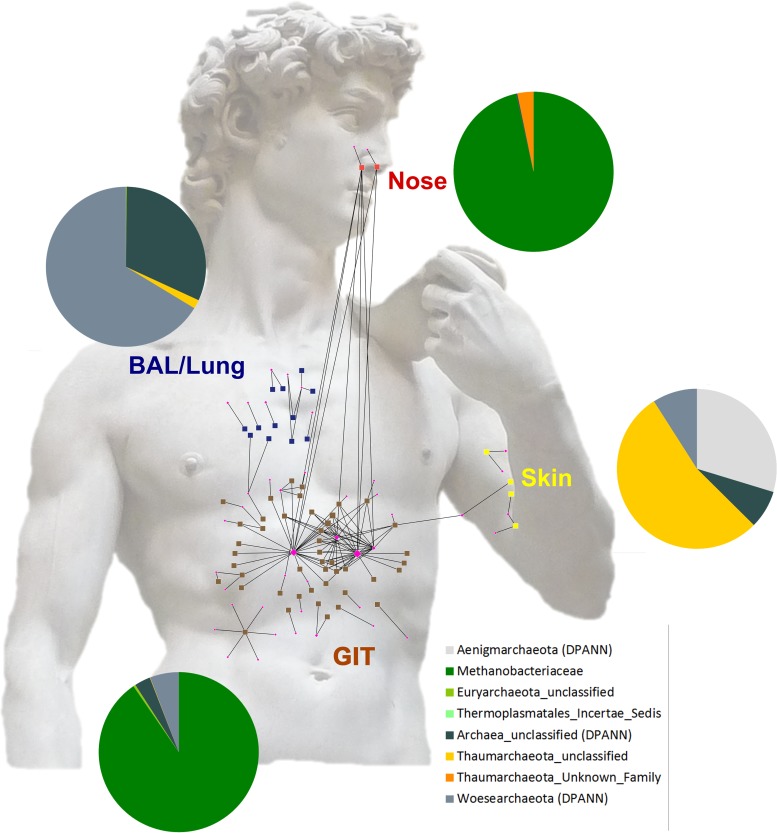
The archaeal taxonomic landscape of the human body visualized as a network. RSVs (DADA2 output) obtained from four sampling sites (BAL fluid/lung, blue; skin, yellow; nose, red; GIT, brown) are plotted and arranged according to their sampling origins.

OTUs/RSVs of all three processing pipelines were visualized as networks (Text S1; Fig. S6B). For a better overview, the network generated on the basis of the DADA2 output was arranged according to the different body sites as shown in Fig. 6.

The most abundant RSVs (assigned to the genus *Methanobrevibacter*) were connected with archaeal communities from all parts of the GIT. However, some GIT archaeal communities (i.e., from the left colon, right colon, ileum, and corpus) remained unconnected on the RSV level and contained only one RSV per sample. These RSVs were assigned as unclassified *Archaea*, *Woesearchaeota*, or *Euryarchaeota*.

Notably, only one GIT sample (right colon) shared one RSV node (thaumarchaeal signature) with two BAL fluid/lung samples; however, a remarkably high percentage of biopsy samples (56%) were connected with RSVs also abundant in the nose (mainly assigned to *Methanobrevibacter*). Only one skin sample was connected with the antrum sample through a thaumarchaeal node. Overall, archaeal signatures deriving from the GIT were connected to all other body parts (in particular, nasal samples), but skin and lung samples were not well connected with those from other body parts on the basis of RSVs.

### Outlook.

Although our study is one of the first steps in the elucidation of the human archaeome, much more investigation and development is required to assess the abundance (i.e., the ratio of *Bacteria* to *Archaea*), distribution, and function of the human-associated archaea. Other methods and technologies, including visualization, cultivation, and functional *in vivo* studies, are necessary to answer the final question of whether archaea are really “never bad” (59).

In summary, our study highlights the importance of the choice of primers and data processing pipeline for studying the human archaeome. Despite these pitfalls, we were able to establish protocols that allow the specific detection of a broad diversity of human-associated archaea. Moreover, this technique allowed us to identify potential novel archaeal members of the human microbial communities, detect archaeal signatures in the nose and lung, and finally reveal body site specificity for the human archaeome.

## MATERIALS AND METHODS

### General remarks.

Research involving human material was performed in accordance with the Declaration of Helsinki and was approved by the local ethics committees (the Ethics Committee at the Medical University of Graz, Graz, Austria, and the University of Kiel, Kiel, Germany). (Bacterial) microbiome studies of these samples have already been published elsewhere (60–63), and details of the ethics approvals obtained are also shown there. DNA extracted in those previous experiments was used to analyze the archaeome in this study.

### Primer sequences and target genes for the specific detection of archaea in human samples.

The primer sequences used, target genes, and references are shown in Text S1.

### Samples, sample processing, and amplification procedure for the detection of specific methanoarchaeal gene signatures in gut biopsy samples (approach 1).

For this approach, 25 biopsy samples (3 to 20 mg) taken from the lower part of the colon were processed, including 5 samples from healthy volunteers, 10 samples from patients with UC (5 samples from inflamed tissue and 5 from uninflamed tissue), and 10 samples from patients with CD (5 samples from inflamed tissue and 5 from uninflamed tissues). Samples were immediately frozen at −80°C. DNA was isolated by a combination of mechanical lysis (glass beads) and chemical and enzymatic digestion. For details, see Text S1.

DNA extracted from gut biopsy specimens (5 healthy, 5 UC inflamed, 5 UC uninflamed, 5 CD inflamed, 5 CD uninflamed) was subjected to a nested PCR approach specifically targeting *M. smithii*, *M. stadtmanae*, and *M. luminyensis*, the most abundant methanogenic archaea in the human gut. On the basis of the study of Dridi et al. (16), we decided to target the *rpoB* gene of *M. smithii* and *M. stadtmanae* because of its higher specificity than the 16S rRNA gene system. This gene encodes the β subunit of RNA polymerase and has been previously used to unravel phylogenetic relationships within the archaeal domain (16, 64) (Text S1).

DNA extracted from *M. smithii* DSM861, *M. stadtmanae* DSM3091, and *M. luminyensis* B10 DSM25720, obtained from the German Collection of Microorganisms and Cell Cultures (DSMZ), Braunschweig, Germany, served as positive controls, whereas sterile double-distilled water and DNA extracted from *Escherichia coli* K-12 served as negative controls.

Template DNA (100 ng of biopsy sample DNA, 100 pg of control DNA) was added to a 25-µl PCR mixture including GoTaq DNA polymerase (Promega, Mannheim, Germany), 10 µM primer, and 0.4 mM deoxynucleoside triphosphates. For the cycling conditions used, see Text S1.

Following the PCR or nested PCRs, amplified fragments were analyzed by agarose gel electrophoresis. To confirm the identities of the signatures retrieved, selected nested PCR products were subjected to Sanger sequencing at the sequencing facility of the Institute of Clinical Molecular Biology, University of Kiel, Kiel, Germany. For details, see Text S1. As the PCR products obtained were too long for Illumina-based NGS, they were TOPO-TA cloned (see Text S1). For the corresponding sequences, see the appendix in Text S1.

### Stool samples, sample processing, and amplicon generation for NGS-based specific assessment of the diversity of archaea in a natural mock community (method establishment and verification, approach 2).

In our study, stool samples from two healthy adults served as a natural mock community. For sample processing details, see Text S1. All samples were immediately stored at −80°C until DNA isolation. The DNA was extracted by a combination of mechanical lysis and chemical and enzymatic digestion. For details, see Text S1. DNA extracted from two different stool samples, serving as natural mock communities, was subjected to PCR with three primer combinations to evaluate the quality of the sequencing results retrieved by NGS with regard to the diversity of archaea obtained and the specificity of the approach chosen. First, we chose primers 515f and 806r, targeting both *Bacteria* and *Archaea*, which currently represents the primer pair used the most for microbiome studies, as proposed by the HMP and the EMP (43). For the other approaches, we selected more archaea-specific procedures, based on a nested PCR approach using primers 344f and 915r in the first PCR and primer pair S-D-Arch-0349-a-S-17/S-D-Arch-0519-a-A-16 (here 349af/519ar), targeting *Archaea*, or primer pair S-D-Arch-0519-a-S-15/S-D-Bact-0785-b-A-18 (here 519af/785ur), targeting both *Bacteria* and *Archaea*, in the subsequent PCR (44). For the primer sequences and amplification protocols used, see Text S1. Briefly, amplified fragments from the first PCR were gel purified and 1 µl of the eluate obtained was subjected to the subsequent PCR for 25 additional cycles. We used DNA from *Pyrococcus furiosus* as a positive control and PCR grade water as a negative control. All samples were amplified and sequenced in triplicate. All of the data sets retrieved were subjected to data processing as described below.

### Samples, sample processing, and amplicon generation for the NGS-based, specific assessment of the diversity of archaea in human tissue samples (method application, approach 2).

For this part of the study, we processed a variety of human samples, including biopsy specimens from the GIT, BAL fluid, and nasal and skin swab samples (for details, see Text S1). The biopsy samples were collected at the Medical University of Graz from seven different sites in the GIT ([gastric] corpus [*n* = 11], [gastric] antrum [*n* = 11], duodenum [*n* = 11], [terminal] ileum [*n* = 11], appendiceal orifice [appendix; *n* = 11], ascending [right] colon [*n* = 11], and sigmoid [left] colon [*n* = 11]). For biopsy specimen collection, 11 volunteers underwent gastroduodenoscopy, followed by colonoscopy on the next day (60, 61). Patients (>18 years old) were recruited for BAL fluid sampling (*n* = 36). The study subjects were nonneutropenic intubated and mechanically ventilated patients in an intensive care unit (62). Lung microbiome samples were obtained by bronchoscopy of the right lung through endotracheal tubes. Nasal microbiome samples from healthy volunteers were taken by an ear-nose-throat physician from the olfactory mucosa located at the ceiling of the nasal cavity with ultra minitip nylon flocked swabs (Copán, Brescia, Italy; *n* = 2). Skin samples were taken from two volunteers at three different body locations, i.e., the exterior side of the left forearm (*n* = 2), chest (*n* = 2), and back (*n* = 2), with the BD Culture Swabs EZ Collection and Transport system (63).

For the screening of different human samples (biopsy specimens and lung, nose, and skin samples), we chose one amplification approach, namely, the first PCR with primer pair 344f/915r and a subsequent nested PCR with primer pair 349af/519ar as described in Text S1. In this case, amplicons from the first PCR were purified with the MinElute PCR Purification kit (Qiagen, Hilden, Germany). For an overview of the samples processed (type and number) and the approach chosen, see Text S1.

### NGS, bioinformatics, and statistical analyses.

All of the amplicons produced were sequenced at ZMF Core Facility Molecular Biology in Graz, Austria, with the available MiSeq platform (63).

Sequence data processing was performed with three different sequence data analysis programs, namely, QIIME (v1.9.1), mothur (v1.36.1), and DADA2 (v1.1). Data processing with QIIME and mothur was conducted with an in-house Galaxy server setup (65, 66). The parameters for the pipelines were created in accordance with the SOPs provided by their developers (67) (Illumina overview tutorial [http://nbviewer.jupyter.org/github/biocore/qiime/blob/1.9.1/examples/ipynb/illumina_overview_tutorial.ipynb]). Briefly, for QIIME and mothur, paired-end reads were joined, trimmed, and checked for chimeric sequences. OTUs were picked in accordance with the open reference strategy in QIIME and with the average neighbor algorithm in mothur with a cutoff of 97% identity. Taxonomic affiliations were determined with SILVA v123 (68) as the reference database.

The third type of amplicon processing was performed with the open source package DADA2 (39) in R (version 3.2.2) as described previously (50). In general, the DADA2 approach is based on the modeling and correction of amplicon errors without constructing OTUs, and in contrast to mothur and QIIME, DADA2 performs merging of paired-end reads after denoising. In brief, the DADA2 approach turns paired-end fastq files into merged, denoised, chimera-free, and inferred sample sequences, so-called RSVs with fewer incorrect sequences than any OTU method (39). In our analysis, sequences were quality checked, filtered, and trimmed to a consistent length (140 bp). Passed sequences were dereplicated and subjected to the DADA2 core algorithm. After chimera filtering and merging of paired reads, sequences were assigned a taxonomic classification by using the RDP classifier and the SILVA database v123 (68). In the resulting RSV table, each row corresponds to a nonchimeric inferred sample sequence with a separate taxonomic classification.

During data processing, samples with no archaeal OTUs/RSVs were removed and only samples with archaeal signals were processed further.

Every step of the analysis included negative controls consisting of blank DNA extractions for each group of samples and negative controls during PCR amplifications. OTUs/RSVs that overlapped negative controls and samples were removed from the data sets.

After data processing, alpha diversity and beta diversity (RDA) were calculated and visualized with Calypso (69). PCoA (based on Bray-Curtis distance) for OTU/RSV clustering was performed with the package phyloseq (70). The OTU/RSV tables obtained were used to summarize taxon abundance at different taxonomic levels (domain, phylum, class, order, family, and genus). The taxonomic profiles obtained were used to generate bar graphs at the phylum level for all of the body locations investigated, area plots at the genus level for the biopsy specimens from the GIT, and pie charts at the genus level on the DADA2 results for all samples. In the pretest to evaluate the performance of primer pairs in the detection of archaeal OTUs/RSVs in human stool samples, we assessed the differences in the richness of OTUs/RSVs by one-way ANOVA and a *t* test.

To analyze and visualize the diversity of archaea detected in human samples, a phylogenetic tree was constructed on the basis of one representative sequence from each OTU retrieved, as obtained through the QIIME and mothur pipelines, and on the basis of RSV sequences taken from the DADA2 output. To include closely related reference sequences from a quality-checked and regularly updated database, SILVA SINA (71) was used to identify the 10 most closely related available sequences (neighbors). The neighbor sequences were downloaded and included in the retrieved sequence data set. All sequences were aligned and cropped to the same length before a tree based on the maximum-likelihood algorithm (MEGA6) (72) was constructed. The Newick output was processed further with the iTOL interactive online platform (73). Data sets for, e.g., the method or the origin of the OTU were added in accordance with the instructions on the iTOL help pages.

To visualize the relationship between archaeal OTUs/RSVs within the human archaeome, a network analysis of the data sets retrieved from the processing pipelines (QIIME, mothur, and DADA2) was performed. Respective OTUs and RSVs were clustered, and E weights were calculated with a stochastic spring-embedded algorithm. The resulting edge and node tables were visualized with cytoscape 2.8.3 (74). OTUs/RSVs were colored according to their sample origins, and their abundance was correlated with node size.

### Accession number(s).

The MiSeq amplicon sequence data obtained in this study were deposited in the European Nucleotide Archive under study accession number PRJEB19529.
