# Supplementary material for: First Insights into the Diverse Human Archaeome: Specific Detection of Archaea in the Gastrointestinal Tract, Lung, and Nose and on Skin
Source: mBio. 2017 Nov 14;8(6):e00824-17. doi: 10.1128/mBio.00824-17 (PMC5686531; doi:10.1128/mBio.00824-17)
Supplement: TEXT S1 [file mbo005173581s1.docx]

**Text S1**

First insights into the diverse human archaeome: specific archaea detection in the gastrointestinal tract, lung, nose and on skin

Kaisa Koskinen^a,b^, Manuela R. Pausan^a^, Alexandra K. Perras^a,c^, Michael Beck^a*^, Corinna Bang^d *^, Maximilian Mora^a^, Anke Schilhabel^d*^, Ruth Schmitz^d^, Christine Moissl-Eichinger^a,b^#

Medical University of Graz, Department of Internal Medicine, Graz, Austria^a^; BioTechMed-Graz, Graz, Austria^b^; University of Regensburg, Regensburg, Germany^c^; Christian-Albrechts-University Kiel, Kiel, Germany^d^

Running title: First insights into the diverse human archaeome

# Address correspondence to Christine Moissl-Eichinger, christine.moissl-eichinger@medunigraz.at

KK, MRP and AKP contributed equally to this work.

* Current address M. Beck: Roche Diagnostics GmbH, Nonnenwald 2, Penzberg, Germany; Current address C. Bang: University Hospital Schleswig-Holstein, Institute of Clinical Molecular Biology, Kiel, Germany; Current address A. Schilhabel: University Hospital Schleswig-Holstein, Department of Hematology, Kiel, Germany

**Introduction (additional information)**

The specific detection of archaeal signatures suffers from methodological problems on various levels, for example: i) DNA extraction protocols have often been optimised for bacterial DNA extraction, but archaeal cells usually have a more rigid cell wall (1, 2). ii) Most of the 16S rRNA gene primers used in human-associated archaeal studies have a low coverage based on *in silico* analysis (3). As a consequence, primers targeting other genes, such as *mcrA*, *amoA*, *mtaB1*, *NifH* and *cnp60*, have additionally been used to explore the diversity of archaea in human samples (a table summarizing targeted archaeal groups and genes, sample types, molecular methods, and primers which have been used to detect Archaea in human-associated samples is shown in **Table S1**). In case archaea-specific primers are used, a frequent cross-reaction with human (eukaryotic) DNA is observed (4), which could, however, be solved by using a nested PCR approach. iii) The identification of Archaea in human tissue samples is often hindered due to a relatively low abundance of archaeal DNA, and most amplification protocols are not adapted to low abundant DNA. iv) 16S rRNA gene reference databases are, due to the fragmentary information on the archaeal domain, incomplete and thus hinder proper taxonomic affiliation of sequence reads. As a consequence, archaeal 16S rRNA gene information is frequently filtered from the dataset during sequence processing (5). Such problems are also reflected in metagenomic analyses, in which assignable archaeal reads typically represent a small minority as well (e.g. (6)).

**Materials and methods (additional information)**

**Overview on primer sequences and target genes for the specific detection of archaea in human samples**

**Text S1, Table** **A**: Target genes, primer sequences, and references for the specific detection of archaea in human samples. If no reference is given, the primer was designed in this study.

| **Amplified gene** | **Nested PCR round 1°/2°** | **forward primer 5’-à 3’** | **reverse primer 5’-à 3’** | **Reference** |
| --- | --- | --- | --- | --- |
| rpoB *M. smithii* | 1° | Ms_rpoBF | Ms_rpoBR | (7) |
|  |  | CTCACGATAAGGACCATTACAC | CTGAAGGTTCTGATGAACAAGA |  |
|  | 2° | Ms_rpoB2F | Ms_rpoB2R | This study |
|  |  | AAGGGATTTGCACCCAACAC | CCAGAGGGTCCTAACTGTGGTC |  |
| rpoB *M. stadtmanae* | 1° | stadt_rpoBF | stadt_rpoBR | (7) |
|  |  | TGCAGCAGGAGAACTAAAATG | GGTGGACAAGAAGATCACTTTG |  |
|  | 2° | Mst_rpoB2F | Mst_rpoB2R | This study |
|  |  | TGCTTGGTATTTGTGCTGGA | TGACAAAACAGGCTCTTGGA |  |
| 16S rRNA *M. luminyensis* | 1° | M.lum.16S-1F | M.lum.16S-1R | This study |
|  |  | GCCTAAAACGTCCGTAGCC | AAGCCCATTGTCCCGTC |  |
|  | 2° | M.lum.16S-1F | M.lum.16S-1R | This study |
|  |  | TCAACGCCGAAAAACTCAC | GACCTCTCCTTCCTCTGAC |  |
| 16S rRNA Archaea universal 1. | 1° | 344f | 915r | 344f: (8) |
|  |  | ACGGGGYGCAGCAGGCGCGA | GTGCTCCCCCGCCAATTCCT | 915r: (9) |
|  | 2° | SD-Ar-349aS17 | SD-Ar-519aA16 | 349f: (10) |
|  |  | GYGCASCAGKCGMGAAW | TTACCGCGGCKGCTG | 519r: (11) |
| 16S rRNA Archaea universal 2. | 1° | 344f | 915r | 344f: modified from (8) |
|  |  | ACGGGGYGCAGCAGGCGCGA | GTGCTCCCCCGCCAATTCCT | 915r: (9) |
|  | 2° | SD-Ar-519aS15 | SD-Ar-0785bA18 | 519f: (12) |
|  |  | CAGCMGCCGCGGTAA | TACNVGGGTATCTAATCC | 785r: (13) |
| 16S rRNA universal |  | 515F | 806R | (14) |
|  |  | GTGCCAGCMGCCGCGGTAA | GGACTACHVGGGTWTCTAAT |  |

**Sample processing and amplification procedure for the detection of specific methanoarchaeal gene signatures in gut biopsy samples (approach 1)**

***DNA extraction from gut biopsy samples (approach 1)***

Prior to DNA isolation, the 25 biopsy samples were transferred to 2 ml-tubes containing 50 µl sterile PeiW buffer (15) and glass beads (0.1 mm and 2.5 mm) for mechanical cell disruption for 2 min at 1,300 strokes/min at room temperature using the Geno/Grinder 2000 (BT&C/OPS Diagnostics, Bridgewater, NJ, USA). Subsequently, 5 µl dithiothreitol and PeiW (0.02 U) enzyme in the respective PeiW buffer were added to each sample and incubated for 30 min at 71 °C according to the protocol of Luo *et al*. under anaerobic conditions (15). Next, genomic DNA of biopsy samples was isolated using the RTP® Bacteria DNA Mini Kit (STRATEC Molecular, Birkenfeld, Germany) following the manufacturer`s instruction (protocol 5) and used for nested-PCR approaches described in the main manuscript. The detailed information on used primers and cycling conditions are summarized in Table B of Text S1.

**Text S1, Table** **B**: Primer combinations and cycling conditions for approach 1.

| **Amplified gene** | **Nested PCR round 1°/2°** | **forward primer 5’-à 3’/ reverse primer 5’-à 3’** | **Initial denaturation** | **Denaturation** | **Annealing** | **Elongation** | **Final Elongation** | **No. of cycles** | **Product size (bp)** |
| --- | --- | --- | --- | --- | --- | --- | --- | --- | --- |
| 16S rRNA Archaea universal | 1° | 342F / 1204R | 5’ 95°C | 45'' 95°C | 45'' 55°C | 1.5' 72°C | 10’ 72°C | 30 | 862 |
|  | 2° | 571F / 976R | 5’ 95°C | 45'' 95°C | 45'' 55°C | 45'' 72°C | 10’ 72°C | 30 | 405 |
| rpoB *M. smithii* | 1° | Ms_rpoBF/ Ms_rpoBR | 15’ 95°C | 30'' 95°C | 45'' 54°C | 30'' 72°C | 7’ 72°C | 35 | 457 |
|  | 2° | Ms_rpoB2F/ Ms_rpoB2R | 15’ 95°C | 30'' 95°C | 45'' 56°C | 30'' 72°C | 7’ 72°C | 35 | 70 |
| rpoB *M. stadtmanae* | 1° | stadt_rpoBF/ stadt_rpoBR | 15’ 95°C | 30'' 95°C | 45'' 54°C | 30'' 72°C | 7’ 72°C | 35 | 530 |
|  | 2° | Mst_rpoB2F/ Mst_rpoB2R | 15’ 95°C | 30'' 95°C | 45'' 56°C | 30'' 72°C | 7’ 72°C | 35 | 95 |
| 16S rRNA *M. luminyensis* | 1° | M.lum.16S-1F/ M.lum.16S-1R | 5’ 95°C | 45'' 95°C | 45'' 58°C | 45'' 72°C | 10’ 72°C | 35 | 698 |
|  | 2° | M.lum.16S-1F/ M.lum.16S-1R | 5’ 95°C | 45'' 95°C | 45'' 55°C | 45'' 72°C | 10’ 72°C | 35 | 224 |

***Archaea identification (approach 1)***

In general, the presence of Archaea was confirmed by a nested PCR approach using primers 342F and 1204R in the first PCR, and primer pair 571F and 976R in the second PCR ((16); with minor modifications for 342F; Tables A+B). These PCR products were purified using Gelex Kit (Macherey-Nagel, Düren, Germany). As they were too long for Illumina-based next generation sequencing, PCR products were TOPO-TA cloned according to the manufacturer's protocol (Invitrogen, Darmstadt, Germany). Plasmids of positive DH5α clones were purified from 5 ml overnight cultures grown in Luria Bertani (LB)-medium using the NucleoSpin® Plasmid Quick Pure (Macherey-Nagel, Düren, Germany). M13-specific primer pairs were used for Sanger sequencing at the sequencing facility at the Institute of Clinical Molecular Biology, University of Kiel, Kiel, Germany (IKMB). For strain-specific primer pairs the obtained PCR products were purified (Gelex Kit) and directly sequenced using the respective primer pairs. The detection of the specific strains was confirmed by nucleotide blastn search at NCBI nucleotide database (17). Primer pairs Ms_rpoB, Mst_rpoB, M.lum.16S-1 and M.lum.16S-2 were designed by using NCBI Primer-BLAST with the following parameters: ~ 20 nucleotides in length, GC-content above 50%, optimal melting temperature 58 °C as well as PCR product sizes between 400 and 800 bp for first PCR reactions and smaller than 250 bp for the second ones (18).

**Stool samples, sample processing and amplicon generation for the NGS based, specific assessment of archaeal diversity in a natural mock community (method establishment and verification; approach 2)**

DNA from stool samples provided by two healthy persons was extracted using E.Z.N.A stool DNA kit (VWR International/ Omega Biotek; included a bead-beating step) according to manufacturer’s instructions. DNA concentrations were measured with Qubit dsDNA HS Assay Kit (Thermo Fisher Scientific, USA), and were: 23.2 ng/μl (human stool #1) and 14.7 ng/μl (human stool #2).

The stool samples were subjected to PCR with three primer combinations, 515f/806r, 349f/519r (344/915), and 519f/785r(344/915). Details about primer combinations and cycling conditions are given in Table C.

**Text S1, Table** **C**: Primer combinations and cycling conditions for Approach 2.

| **Amplified gene** | **Nested PCR round 1°/2°** | **forward primer 5’-à 3’/ reverse primer 5’-à 3’** | **Initial denaturation** | **Denaturation** | **Annealing** | **Elongation** | **Final Elongation** | **No. of cycles** | **Product size (bp)** |
| --- | --- | --- | --- | --- | --- | --- | --- | --- | --- |
| 16S rRNA | 1° | 344f/915r | 2’ 95°C | 30'' 96°C (first 10 cycl.), 25'' 94°C | 30'' 60°C | 1’ 72°C | 10’ 72°C | 25 | 571 |
|  | 2° | Illu-SD-Ar-349aS17/ Illu-SD-Ar-519aA16 | 5’ 95°C | 40'' 95°C | 2’ 63°C | 1’ 72°C | 10’ 72°C | 25 | 170 |
| 16S rRNA | 1° | 344f/915r | 2’ 95°C | 30'' 96°C (first 10 cycl.), 25'' 94°C | 30'' 60°C | 1’ 72°C | 10’ 72°C | 25 | 571 |
|  | 2° | Illu-SD-Ar-519aS15/ Illu-SD-Ar-0785bA18 | 5’ 95°C | 40'' | 2’ 63°C | 1’ 72°C | 10’ 72°C | 25 | 266 |
| 16S rRNA |  | Illu_515F/ Illu_806R | 3’ 94°C | 45'' 94°C | 60'' 50°C | 1.5' 72°C | 10’ 72°C | 35 | 191 |

For purification of PCR products between the first and second round of nested PCR, we applied gel purification method. The gel-chamber and all equipment were treated with DNA-ExitusPlus™ beforehand (application time: minimum 10 min.), and the fragments were cut on a gel-documentation station using a DNA-free scalpel. For every PCR product a new DNA-ExitusPlus™ treated scalpel was used. One µl of the obtained eluate was subjected to the subsequent PCR for 25 additional cycles.

The PCR reaction mixture contained TAKARA Ex Taq® buffer with MgCl_2_ (10 X; Takara Bio Inc., Tokyo, Japan), primers 300 nM, BSA (Roche Lifescience, Basel, Switzerland) 1 mg/ml, dNTP mix 200 µM, TAKARA Ex Taq® Polymerase 0.5 U, and water (Lichrosolv®; Merck, Darmstadt, Germany) up to total volume of 20 µl. We used genomic DNA from *Pyrococcus furiosus* as a positive control and PCR grade water as negative control. All samples were amplified and sequenced in triplicates.

**Samples, sample processing and amplicon generation for the NGS based, specific assessment of archaeal diversity in human tissue samples (method application)**

***Gastrointestinal tract biopsy samples***

The gastrointestinal tract biopsy samples were collected at Medical University of Graz from 7 different sites in the GIT (corpus, antrum, duodenum, ileum, appendix, right colon, and left colon). For the biopsy collection, 11 volunteers underwent a gastroduodenoscopy followed by a colonoscopy on the next day. The samples were immediately stored at −80 °C until DNA isolation. MagNA Pure LC DNA Isolation Kit III (bacteria, fungi) were used for automated DNA isolation according to the manufacturer’s instructions. Detailed description of sampling and DNA isolation procedures are described in Bashir *et al*. (19) and Kump *et al*. (20).

***Broncho-alveolar-lavage (BAL) samples***

Broncho-alveolar-lavage (BAL) samples were collected at Department of Internal Medicine, Medical University of Graz. Patients of >18 years old were recruited for the lung microbiome sampling. The study subjects were non-neutropenic intubated and mechanically ventilated patients at intensive care unit. All study subjects gave written informed consent for study participation. Unconscious intubated and mechanically ventilated patients were asked for study participation after their awakening according to approval of the ethical committee.

Lung microbiome samples (Bronchoalveolar lavage; BAL) of the right lung (20 ml normal saline) were obtained by bronchoscopy through endotracheal tubes. The samples were immediately brought to the inhouse microbiology laboratory, and stored at -70°C until further processing. The total DNA was isolated with a combination of mechanical and enzymatic lysis with the MagnaPure LC DNA Isolation Kit III (Bacteria, Fungi; Roche, Mannheim, Germany) as described in Krause *et al*. (21).

***Nose microbiome samples***

The nose microbiome samples of healthy volunteers were taken by an ENT physician from the olfactory mucosa using ultra minitip nylon flocked swab (COPAN), located at the ceiling of the nasal cavity. The sample tubes were placed on dry ice immediately after sampling, and in laboratory the samples were stored at -80 °C until further processing. Genomic DNA was extracted from the swabs using FastDNA SPIN Kit (MP Biomedicals, Germany) according to manufacturer’s instructions. Concentration of the isolated DNA was quantified with Qubit dsDNA HS Assay Kit (Thermo Fisher Scientific, USA).

***Skin microbiome samples***

The skin samples were taken from two volunteers at three different body locations: exterior side of the left forearm, chest, and back using the BD Culture SwabsTM EZ Collection and Transport system. The total DNA was isolated with a combination of mechanical and enzymatic lysis with the MagnaPure LC DNA Isolation Kit III (Bacteria, Fungi; Roche, Mannheim, Germany) as described in Klymiuk *et al*. (22).

An overview on all used samples is given in Table D.

**Text S1, Table** **D**: Overview on retrieved samples and the selected primers.

| **Sample type** | **Description** | **Primer pair** |
| --- | --- | --- |
| Gastrointestinal tract (GIT) microbiome (biopsy) | Corpus, 11 samples | S-D-Arch-0**349**-a-S-17/  S-D-Arch-0**519**-a-A-16 (344f/915r) |
|  | Antrum, 11 samples |  |
|  | Duodenum, 11 samples |  |
|  | Ileum, 11 samples |  |
|  | Appendix, 11 samples |  |
|  | Right colon, 11 samples |  |
|  | Left colon, 11 samples |  |
| Lung microbiome | Bronchoalveolar lavage, 36 samples |  |
| Nose microbiome | Olfactory mucosa swab, 2 samples |  |
| Skin microbiome (swab) | Left forearm, 2 samples  Chest, 2 samples  Back, 2 samples |  |

**Results (additional information)**

**The choice of the sequence processing pipeline affects the retrievable information on the human archaeome**

Here we aimed to analyze the archaeal diversity in various human microbiome samples, including gut biopsy samples, lung (bronchoalveolar lavage, BAL), olfactory mucosa (nose) and skin samples taken from forearm, chest and back. The raw sequence data set was processed through the three pipelines based on: QIIME, mothur, and DADA2.

The archaeal alpha diversity was determined for each body site by calculating the inverse Simpson diversity index based on the respective OTU/RSV table obtained from each processing pipeline (**Table S2**). In all datasets, the nose samples showed the highest archaeal diversity, followed by skin (mothur and DADA2) and gut samples (QIIME; see **Fig. S2**), whereas the lowest diversity was retrieved from lung samples. The difference of the alpha diversity between the body sites was found to be significant only in the results obtained by DADA2 (p=0.0486; see **Fig. S2**).

The three different processing pipelines provided a relatively similar picture of the archaeal communities associated with the different body sites (**Fig. S5**). All datasets confirmed the predominance of thaumarchaeal signatures on human skin, and a predominance of methanogenic Euryarchaeota in the human GIT. Discrepancies were found, however, in the BAL (lung) datasets with respect to the classification of the DPANN associated archaea (**Fig. S5**). It has to be noted, that the DPANN superphylum is not yet well covered by reference sequences of high quality, causing most likely classification problems. Although the mothur output did not assign further taxonomic information on the DPANN sequences, DADA2 and QIIME assigned most of these sequences to the Woesearchaeota phylum. Notably, DADA2 even allowed the classification of retrieved sequences from the Aenigmarchaeota phylum (skin samples) and the QIIME pipeline retrieved Diapherotrites (GIT and nose). Although we queried OTUs/RSVs against the same database (SILVA v123), a different picture of classification resulted by using the three processing pipelines. This might be explainable by the varying or rather absent clustering methods.

**Network analyses reveal the connection of the archaeal communities on OTU/RSV level**

OTUs/RSVs of all three processing pipelines were visualized as networks (**Fig. S6**).

The network of OTUs retrieved from the QIIME pipeline appeared as most multifaceted, followed by a size-reduced network for the mothur dataset and an even smaller network generated for RSVs (DADA2). The number of nodes represents the number of OTUs/RSVs, so less OTUs/RSVs result in a smaller network. For a better overview, the network generated based on the DADA2 output was arranged according to the different body sites and is visualized in **Fig. 6**.

**Discussion (additional information)**

Human-associated archaea remain, similar to fungi or viruses, understudied in the field of microbiome research. In this study, we focused on primer choice and sequence processing protocols in order to optimize specific detection of archaea in human samples.

**A PCR based, methanoarchaea targeting approach allows the specific detection of human-associated methanogens (*M. smithii, M. stadtmanae* and *M. luminyensis*) in healthy and inflamed gut biopsy samples (approach 1)**

In a first approach tested in this study, we focused on the facilitated, NGS-independent detection of the three most abundant methanoarchaea of the human gut to answer the question, whether there is a co-occurrence pattern of various mucosa-associated methanoarchaeal strains with the manifestation of inflammatory processes involving the human gastrointestinal tract. To explore this proposed pattern, general Archaea-specific and strain-specific nested PCR approaches were established. A total of 25 GIT biopsies were investigated for the presence of specific strains, namely *M. smithii*, *M. stadtmanae* and *M. luminyensis*. 21 samples out of 25 have shown a positive signal for *M. smithii*, but only in 8 samples *M. stadtmanae* was detected, and *M. luminyensis* was found in 7 samples.

Our results nicely reflect earlier results obtained from stool samples, which have shown that *M. smithii* is present in 90% of all subjects, while *M. stadtmanae* is present in only around 30% and *M. luminyensis* was found only in 6% of all investigated subjects (7, 23).

Noteworthy, the herein shown presence of methanoarchaea in biopsy samples suggest that these microorganisms most likely are associated with the mucosa and are not only present in the lumen of the GIT. *In vitro* experiments have shown that methanogens are able to activate immune cells (24), and several studies have indicated a change in archaeal abundance (especially methanogens) in colon biopsies or stool samples of patients with gastrointestinal disorders such as: irritable bowel syndrome (IBS) (25), colorectal cancers (26), inflammatory bowel disease (IBD) (27), obesity (28) and constipation (25, 29), proposing a potential involvement of archaea in human disease. However, many of these results are contradictory, especially the linkage between methanogens and obesity. Some studies have shown that the methanogenic archaeal abundance is higher in obese patients (28), while others have shown that methanogens are more abundant in lean subjects (30, 31). One study with gnotobiotic mice has shown that the association between *M. smithii* and *Bacteroides thetaiotaomicron* affected the energy harvest from dietary glycans, leading to an increased body mass of the mice that were co-colonized by these two microorganisms (32).

As *Methanobrevibacter* only supports associated bacteria by efficient hydrogen consumption, the observed changes in the archaeal abundance in different gastrointestinal disorders could be rather an effect than a cause. Similar processes are discussed for *M. oralis*, whose abundance was significantly increased in periodontitis patients (4, 33). While there is research effort on elucidating the archaeal role in human disease, additional focus should be directed on the potential positive influences of certain archaea such *M. luminyensis*, which has the metabolic capability to detract trimethylamine (TMA) from the gut, a potential factor for arteriosclerosis (“archaebiotics concept”, (34)).

**Primer pairs and sequence data processing pipelines determine the detection of archaeal operational taxonomic units (OTUs) and ribosomal sequence variants (RSVs) in natural mock communities (approach 2)**

With respect to NGS-dependent archaeome analyses, we firstly compared three primer pairs to detect Archaea in human stool samples (natural mock community) namely 515f/806r targeting Bacteria and Archaea (14), 349af/519ar (344f/915r) targeting Archaea, and 519af/785ur (344f/915r) targeting Bacteria and Archaea (3). We found that the universal and widely used primer pair 515f/806r did not work satisfactorily for detecting Archaea in human stool.

However, in silico analysis shows that the primer pair has a fairly good coverage of 52% for all Archaea without a mismatch, while also matching 86.5% of the bacterial domain without a mismatch (SILVA test prime search function (3)). With one mismatch allowed, it targets most archaeal phyla (Crenarchaeota, Euryarchaeota, Korarchaeota, Thaumarchaeota, Woesearchaeota, Pacearchaeota, and Aenigmarchaeota), and in particular the class Methanobacteria, including genera Methanosphaera and Methanobrevibacter, the predominant archaea in the human GIT. Also Methanomassiliicoccus should, based on in silico analyses, be targeted by this primer pair. However, our experiments with stool samples allowed the retrieval of maximal three different archaeal taxa amongst up to more than 20.000 bacterial OTUs.

Obviously, the high complexity of the stool samples, such as varying DNA primary structures of different community members and different template starting concentrations in a PCR mixture, as well as possible presence of inhibitors, problems in efficient cell wall lysis, and not optimal amplification protocols lead to unequal amplification of Bacteria and Archaea (35).

Due to aforementioned reasons, we decided to perform nested PCR for the specific amplification of archaeal signatures. Based on our previous studies (36) we applied primer pair **344f/915r** at the first step of nested PCR, which is very selective for the archaeal domain: without mismatches it targets 45,4% of all Archaea in SILVA SSU 128 database, but no Bacteria and Eukaryotes. Based on in silico analyses, this primer proved suitable, as it covered well the phyla Crenarchaeota, Thaumarchaeota, Woesearchaeota, Diapherotrites and Euryarchaeota, including Halobacteria and GIT associated methanoarchaeal groups.

The second step of nested PCR was performed with two different primer pairs with a rather broad target variety: primer pair 519af/785ur (coverage without mismatch: 88,7% of Archaea and 88,5% of Bacteria) and primer pair 349af/519ar (coverage without mismatch: 78,7% of Archaea and 0% of Bacteria). Primer combination 349af/519ar (344f/915r) was, as expected, found to be the most specific approach, whereas 519af/785ur (344f/915r) was capable of retrieving the broadest diversity of Archaea.

We are aware that nested PCR introduces biases into the detected microbial community composition and diversity. Although we detected the largest number of archaeal OTUs/RSVs using combination 519af/785ur (344f/915r), we most likely also missed taxa due to the double selection by consecutive amplifications with two different primer pairs. In general, nested PCR method is valuable when samples with very low DNA concentration need to be amplified, or when inhibitors hinder the reaction with larger quantity of starting material. However, the bias is more substantial for communities with higher diversity, and when more cycles are employed in the first PCR round; in general, nested PCR should be applied when standard PCR is not successful (37), as observed for human samples in this study and in previous ones (36). To reduce the bias introduced with nested PCR, the first PCR was run for only 25 cycles.

Our next generation sequencing approach was based on the application of the Illumina MiSeq platform to study the community composition and diversity of Archaea in human body tissue samples. Due to the depth of the sequencing method, even the rare biosphere can be uncovered. In general, the NGS methods overcome many problems that biased the results with traditional microbiology methods, including cultivation and cloning techniques. However, the Illumina data error profiles have not yet been well understood, and the widely used publicly available sequence data analysis pipelines have originally not been designed for the characteristics of Illumina data (38). In our study, we were particularly interested in the impact of the chosen sequence processing platforms, namely QIIME, mothur and DADA2 on the acquired results.

Mothur is an open source software package that provides a comprehensive pipeline to analyzy community sequence data (39). It was published in year 2009, at the same time when a growing number of scientists started to study diverse microbial communities by applying pyrosequencing technology, and the demand for such tools was rapidly increasing. Next year, Caporaso and colleagues published another open source software pipeline called QIIME (“quantitative insights into microbial ecology”), which aimed to solve the problem of massive 454 datasets at the time (40). While mothur favored the classic hierarchical clustering algorithms due to their accuracy, QIIME applied heuristics to cluster the sequence reads into OTUs to reduce the computational power required, and allowed analysis of large datasets without access to powerful computer facilities.

However, mothur and QIIME were created before Illumina became the standard in amplicon sequencing, and the standard operating procedures, although they were frequently improved and adapted, have not been originally developed to tackle the specific sequencing errors of Illumina technology. Consequently, the errors are addressed by quality filtering and clustering the sequence reads into operational taxonomic units (OTUs), which reduce the error rate but the fine scale variation is left undetected. This fine scale variation can be substantial regarding the study set up, for example differentiating between closely related taxa in a complex community (41).

DADA2 (Divisive Amplicon Denoising Algorithm), published 2016, is an open-source software package designed to model and correct Illumina-sequenced amplicon errors in particular. It has been observed to identify more variants and produce fewer spurious sequence reads compared to other sequence processing tools (41). DADA2 does not have a clustering step, and it is a full amplicon workflow with steps including filtering, dereplication, sample inference, chimera identification, and merging of paired-end reads. The different principle of DADA2 was clearly reflected in our results, as DADA2 retrieved approximately 200 bacterial RSVs from the unspecific approach with primer pair 515f/806r, whereas mothur detected more than 20.000 OTUs in the same sequence data sets. The same trend was observed for archaeal OTUs and RSVs, respectively.

Overall, the most conservative, and thus most stringent, approach was found to be represented by the combination of primer pair 349af/519ar (344f/915r) amplicons with data processing through DADA2. As we consider the findings of this combination as the most trustworthy and reliable information, we decided to base our additional experiments mainly on this approach.

**Supplementary references**

1. Kandler O, König H. 1998. Cell wall polymers in Archaea (Archaebacteria). Cell Mol Life Sci C 54:305–308.

2. König H, Claus H, Varma A (Ajit). 2010. Prokaryotic cell wall compounds : structure and biochemistry. Springer.

3. Klindworth A, Pruesse E, Schweer T, Peplies J, Quast C, Horn M, Glockner FO. 2013. Evaluation of general 16S ribosomal RNA gene PCR primers for classical and next-generation sequencing-based diversity studies. Nucleic Acids Res 41:e1–e1.

4. Horz H-P, Conrads G. 2011. Methanogenic *Archaea* and oral infections – ways to unravel the black box. J Oral Microbiol 3:5940.

5. Ding T, Schloss PD. 2014. Dynamics and associations of microbial community types across the human body. Nature 509:357–360.

6. Oh J, Byrd AL, Deming C, Conlan S, Barnabas B, Blakesley R, Bouffard G, Brooks S, Coleman H, Dekhtyar M, Gregory M, Guan X, Gupta J, Han J, Ho S, Legaspi R, Maduro Q, Masiello C, Maskeri B, McDowell J, Montemayor C, Mullikin J, Park M, Riebow N, Schandler K, Schmidt B, Sison C, Stantripop M, Thomas J, Thomas P, Vemulapalli M, Young A, Kong HH, Segre JA. 2014. Biogeography and individuality shape function in the human skin metagenome. Nature 514:59–64.

7. Dridi B, Henry M, El Khechine A, Raoult D, Drancourt M. 2009. High prevalence of Methanobrevibacter smithii and Methanosphaera stadtmanae detected in the human gut using an improved DNA detection protocol. PLoS One 4:e7063–e7063.

8. Raskin L, Stromley JM, Rittmann BE, Stahl DA. 1994. Group-specific 16S rRNA hybridization probes to describe natural communities of methanogens. Appl Environ Microbiol 60:1232–40.

9. Stahl DA. 1991. Development and application of nucleic acid probes. Nucleic acid Tech Bact Syst.

10. Takai K, Horikoshi K. 2000. Rapid detection and quantification of members of the archaeal community by quantitative PCR using fluorogenic probes. Appl Environ Microbiol 66:5066–5072.

11. Ovreås L, Forney L, Daae FL, Torsvik V. 1997. Distribution of bacterioplankton in meromictic Lake Saelenvannet, as determined by denaturing gradient gel electrophoresis of PCR-amplified gene fragments coding for 16S rRNA. Appl Environ Microbiol 63:3367–3373.

12. Wang Y, Qian P-Y. 2009. Conservative fragments in bacterial 16S rRNA genes and primer design for 16S ribosomal DNA amplicons in metagenomic studies.

13. Claesson MJ, Wang Q, O’Sullivan O, Greene-Diniz R, Cole JR, Ross RP, O’Toole PW. 2010. Comparison of two next-generation sequencing technologies for resolving highly complex microbiota composition using tandem variable 16S rRNA gene regions. Nucleic Acids Res gkq873.

14. Caporaso JG, Lauber CL, Walters WA, Berg-Lyons D, Huntley J, Fierer N, Owens SM, Betley J, Fraser L, Bauer M. 2012. Ultra-high-throughput microbial community analysis on the Illumina HiSeq and MiSeq platforms. ISME J 6:1621–1624.

15. Luo Y, Pfister P, Leisinger T, Wasserfallen A. 2002. Pseudomurein endoisopeptidases PeiW and PeiP, two moderately related members of a novel family of proteases produced in *Methanothermobacter* strains. FEMS Microbiol Lett 208:47–51.

16. Baker GC, Smith JJ, Cowan DA. 2003. Review and re-analysis of domain-specific 16S primers. J Microbiol Methods 55:541–55.

17. Altschul SF, Gish W, Miller W, Myers EW, Lipman DJ. 1990. Basic local alignment search tool. J Mol Biol 215:403–410.

18. Ye J, Coulouris G, Zaretskaya I, Cutcutache I, Rozen S, Madden TL. 2012. Primer-BLAST: A tool to design target-specific primers for polymerase chain reaction. BMC Bioinformatics 13:134.

19. Bashir M, Prietl B, Tauschmann M, Mautner SI, Kump PK, Treiber G, Wurm P, Gorkiewicz G, Högenauer C, Pieber TR. 2016. Effects of high doses of vitamin D3 on mucosa-associated gut microbiome vary between regions of the human gastrointestinal tract. Eur J Nutr 55:1479–1489.

20. Kump PK, Gröchenig H-P, Lackner S, Trajanoski S, Reicht G, Hoffmann KM, Deutschmann A, Wenzl HH, Petritsch W, Krejs GJ, Gorkiewicz G, Högenauer C. 2013. Alteration of Intestinal Dysbiosis by Fecal Microbiota Transplantation Does not Induce Remission in Patients with Chronic Active Ulcerative Colitis. Inflamm Bowel Dis 19:2155–2165.

21. Krause R, Halwachs B, Thallinger GG, Klymiuk I, Gorkiewicz G, Hoenigl M, Prattes J, Valentin T, Heidrich K, Buzina W, Salzer HJF, Rabensteiner J, Prüller F, Raggam RB, Meinitzer A, Moissl-Eichinger C, Högenauer C, Quehenberger F, Kashofer K, Zollner-Schwetz I. 2016. Characterisation of Candida within the Mycobiome/Microbiome of the Lower Respiratory Tract of ICU Patients. PLoS One 11:e0155033.

22. Klymiuk I, Bambach I, Patra V, Trajanoski S, Wolf P. 2016. 16S based microbiome analysis from healthy subjects’ skin swabs stored for different storage periods reveal phylum to genus level changes. Front Microbiol 7.

23. Dridi B, Henry M, Richet H, Raoult D, Drancourt M. 2012. Age-related prevalence of Methanomassiliicoccus luminyensis in the human gut microbiome. Apmis 120:773–777.

24. Bang C, Weidenbach K, Gutsmann T, Heine H, Schmitz RA. 2014. The intestinal archaea Methanosphaera stadtmanae and Methanobrevibacter smithii activate human dendritic cells. PLoS One 9:e99411.

25. Kim G, Deepinder F, Morales W, Hwang L, Weitsman S, Chang C, Gunsalus R, Pimentel M. 2012. Methanobrevibacter smithii is the predominant methanogen in patients with constipation-predominant IBS and methane on breath. Dig Dis Sci 57:3213–3218.

26. Scanlan PD, Shanahan F, Marchesi JR. 2008. Human methanogen diversity and incidence in healthy and diseased colonic groups using mcrA gene analysis. BMC Microbiol 8:79.

27. Lecours PB, Marsolais D, Cormier Y, Berberi M, Haché C, Bourdages R, Duchaine C. 2014. Increased prevalence of methanosphaera stadtmanae in inflammatory bowel diseases. PLoS One 9:1–7.

28. Zhang H, DiBaise JK, Zuccolo A, Kudrna D, Braidotti M, Yu Y, Parameswaran P, Crowell MD, Wing R, Rittmann BE, Krajmalnik-Brown R. 2009. Human gut microbiota in obesity and after gastric bypass. Proc Natl Acad Sci U S A 106:2365–2370.

29. Furnari M, Savarino E, Bruzzone L, Moscatelli A, Gemignani L, Gianini EG, Zentilin P, Dulbecco P, Savarino V. 2012. Reassessment of the role of methane production between irritable bowel syndrome and functional constipation. J Gastrointest Liver Dis 21:157–163.

30. Million M, Maraninchi M, Henry M, Armougom F, Richet H, Carrieri P, Valero R, Raccah D, Vialettes B, Raoult D. 2012. Obesity-associated gut microbiota is enriched in Lactobacillus reuteri and depleted in Bifidobacterium animalis and Methanobrevibacter smithii. Int J Obes 36:817–825.

31. Armougom F, Henry M, Vialettes B, Raccah D, Raoult D. 2009. Monitoring Bacterial Community of Human Gut Microbiota Reveals an Increase in Lactobacillus in Obese Patients and Methanogens in Anorexic Patients. PLoS One 4:e7125.

32. Samuel BS, Gordon JI. 2006. A humanized gnotobiotic mouse model of host-archaeal-bacterial mutualism. Proc Natl Acad Sci U S A 103:10011–6.

33. Nguyen-Hieu T, Khelaifia S, Aboudharam G, Drancourt M. 2013. Methanogenic archaea in subgingival sites: A review. Apmis 121:467–477.

34. Brugère J-F, Borrel G, Gaci N, Tottey W, O’Toole PW, Malpuech-Brugère C. 2014. Archaebiotics. Gut Microbes 5:5–10.

35. Kalle E, Kubista M, Rensing C. 2014. Multi-template polymerase chain reaction. Biomol Detect Quantif 2:11–29.

36. Probst AJ, Auerbach AK, Moissl-Eichinger C. 2013. Archaea on Human Skin. PLoS One 8:e65388.

37. Yu G, Fadrosh D, Goedert JJ, Ravel J, Goldstein AM. 2015. Nested PCR Biases in Interpreting Microbial Community Structure in 16S rRNA Gene Sequence Datasets. PLoS One 10:e0132253.

38. Schirmer M, Ijaz UZ, D’Amore R, Hall N, Sloan WT, Quince C. 2015. Insight into biases and sequencing errors for amplicon sequencing with the Illumina MiSeq platform. Nucleic Acids Res 43:e37–e37.

39. Schloss PD, Westcott SL, Ryabin T, Hall JR, Hartmann M, Hollister EB, Lesniewski RA, Oakley BB, Parks DH, Robinson CJ. 2009. Introducing mothur: open-source, platform-independent, community-supported software for describing and comparing microbial communities. Appl Environ Microbiol 75:7537–7541.

40. Caporaso JG, Kuczynski J, Stombaugh J, Bittinger K, Bushman FD, Costello EK, Fierer N, Peña AG, Goodrich JK, Gordon JI, Huttley GA, Kelley ST, Knights D, Koenig JE, Ley RE, Lozupone CA, McDonald D, Muegge BD, Pirrung M, Reeder J, Sevinsky JR, Turnbaugh PJ, Walters WA, Widmann J, Yatsunenko T, Zaneveld J, Knight R. 2010. QIIME allows analysis of high-throughput community sequencing data. Nat Methods 7:335–6.

41. Callahan BJ, McMurdie PJ, Rosen MJ, Han AW, Johnson AJA, Holmes SP. 2016. DADA2: High-resolution sample inference from Illumina amplicon data. Nat Methods 13:581–583.

**Appendix: Retrieved Sanger sequences from approach 1.**

Healthy Control #2

HC2.1 for

TTCGACTACTATAGGGCGAATTGGGCCCTCTAGATGCATGCTCGAGCGGCCGCCAGTGTG

ATGGATATCTGCAGAATTCGCCCTTCAGGCGTTGAATCCAATTAAACCGCAGGCTCCACG

CGTTGTGGTGCTCCCCCGCCAATTCCTTTAAGTTTCAGTCTTGCGACCGTACTTCCCAGG

CGGCGGACTTAACAGCTTCCCTTCGGCACTGGGACAGCTCAAAGCCACCCCAACACCAAG

TCCGCATCGTTTACAGCTAGGACTACCCGGGTATCTAATCCGGTTCGCGCCCCTAGCTTT

CGTCCCTCACCGTCAGAATCGTTCCAGTCAGACGCCTTCGCAACAGGCGGTCCTCCCAGG

ATTACAGAATTTCACCTCTACCCTGGGAGTACCTCTAACCTCTCCCGATCTCAAGTCTAA

TAGTATCTCCAGCAATTCCCACAGTTAAGCTGCAGGATTTCACCAGAGACTTATTAAACC

GGCTACGGCTGCTTTAAGCAAGGGCGAATTCCAGCACACTGGCGGCCGTTACTAGTGGAT

CCGAGCTCGGTACCAAGCTTGATGCATAGCTTGAGTATTCTATAGTGTCACCTAAATAGC

TTGGCGTAATCATGGTCATAGCTGTTTCCTGTGTGAAATTGTTATCCGCTCACAATTCCA

CACAACATACGAGCCGGAAGCATAAAGTGTAAAGCCTGGGGTGCCTAATGAGTGAGCTAA

CTCACATTAATTGCGTTGCGCTCACTGCCCGCTTTCCAGTCGGGAAACCTGTCGTGCCAG

CTGCATTAATGAATCGGCCAACGCGCGGGAGAGGCGGTTTGCGTATTGGGCGCTCTTCCG

CTTCCTCGCTCACTGACTCCGCTGCGCTCGGTCGTTCGGCTGCGGCGAGGCGGTATTCAG

CTCAC

HC2.1 rev

TACCGCCCGGCTTTTAGGTGACACTATAGAATACTCAAGCTATGCATCAAGCTTGGTACC

GAGCTCGGATCCACTAGTAACGGCCGCCAGTGTGCTGGAATTCGCCCTTGCTTAAAGCAG

CCGTAGCCGGTTTAATAAGTCTCTGGTGAAATCCTGCAGCTTAACTGTGGGAATTGCTGG

AGATACTATTAGACTTGAGATCGGGAGAGGTTAGAGGTACTCCCAGGGTAGAGGTGAAAT

TCTGTAATCCTGGGAGGACCGCCTGTTGCGAAGGCGTCTGACTGGAACGATTCTGACGGT

GAGGGACGAAAGCTAGGGGCGCGAACCGGATTAGATACCCGGGTAGTCCTAGCTGTAAAC

GATGCGGACTTGGTGTTGGGGTGGCTTTGAGCTGTCCCAGTGCCGAAGGGAAGCTGTTAA

GTCCGCCGCCTGGGAAGTACGGTCGCAAGACTGAAACTTAAAGGAATTGGCGGGGGAGCA

CCACAACGCGTGGAGCCTGCGGTTTAATTGGATTCAACGCCTGAAGGGCGAATTCTGCAG

ATATCCATCACACTGGCGGCCGCTCGAGCATGCATCTAGAGGGCCCAATTCGCCCTATAG

TGAGTCGTATTACAATTCACTGGCCGTCGTTTTACAACGTCGTGACTGGGAAAACCCTGG

CGTTACCCAACTTAATCGCCTTGCAGCACATCCCCCTTTCGCCAGCTGGCGTAATAGCGA

AGAGGCCCGCACCGATCGCCCTTCCCAACAGTTGCGCAGCCTGAATGGCGAATGGACGCG

CCCTGTAGCGGCGCATTAAGCGCGGCGGGTGTGGTGGTTACGCGCAGCGTGACCGCTACA

CTTGCCAGCGCCTAGCCGCCCGCTCCTTTCCGCTTTCTTCCCTTCCCTTCTG

HC2.2 for

AGTATCCGACTACTATAGGGCGAATTGGGCCCTCTAGATGCATGCTCGAGCGGCCGCCAG

TGTGATGGATATCTGCAGAATTCGCCCTTGCTTAAAGCGGCCGTAGCCGGTTTAATAAGT

CTCTGGTGAAATCCTGCAGCTTAACTGTGGGAATTGCTGGAGATACTATTAGACTTGAGA

TCGGGAGAGGTTAGAGGTACTCCCAGGGTAGAGGTGAAATTCTGTAATCCTGGGAGGACC

GCCTGTTGCGAAGGCGTCTGACTGGAACGATTCTGACGGTGAGGGACGAAAGCTAGGGGC

GCGAACCGGATTAGATACCCGGGTAGTCCTAGCTGTAAACGATGCGGACTTGGTGTTGGG

GTGGCTTTGAGCTGTCCCAGTGCCGAAGGGAAGCTGTTAAGTCCGCCGCCTGGGAAGTAC

GGTCGCAAGACTGAAACTTAAAGGAATTGGCGGGGGAGCACCACAACGCGTGGAGCCTGC

GGTTTAATTGGATTCAACGCCTGAAGGGCGAATTCCAGCACACTGGCGGCCGTTACTAGT

GGATCCGAGCTCGGTACCAAGCTTGATGCATAGCTTGAGTATTCTATAGTGTCACCTAAA

TAGCTTGGCGTAATCATGGTCATAGCTGTTTCCTGTGTGAAATTGTTATCCGCTCACAAT

TCCACACAACATACGAGCCGGAAGCATAAGTGTAAAGCCTGGGGTGCCTAATGAGTGAGC

TAACTCACATTAATTGCGTTGCGCTCACTGCCCGCTTTCCAGTCGGGAAACCTGTCGTGC

CAGCTGCATTAATGAATCGGCCAACGCGCGGGAGAGGCGGTTTGCGTATTGGGCGCTCTT

CCGCTTTCCTCGCTCACTGACTCGCTGCGCTCGGTCGTTCGGCTGC

HC2.2 rev

AACGCACACTTTTAGGTGACACTATAGAATACTCAAGCTATGCATCAAGCTTGGTACCGA

GCTCGGATCCACTAGTAACGGCCGCCAGTGTGCTGGAATTCGCCCTTCAGGCGTTGAATC

CAATTAAACCGCAGGCTCCACGCGTTGTGGTGCTCCCCCGCCAATTCCTTTAAGTTTCAG

TCTTGCGACCGTACTTCCCAGGCGGCGGACTTAACAGCTTCCCTTCGGCACTGGGACAGC

TCAAAGCCACCCCAACACCAAGTCCGCATCGTTTACAGCTAGGACTACCCGGGTATCTAA

TCCGGTTCGCGCCCCTAGCTTTCGTCCCTCACCGTCAGAATCGTTCCAGTCAGACGCCTT

CGCAACAGGCGGTCCTCCCAGGATTACAGAATTTCACCTCTACCCTGGGAGTACCTCTAA

CCTCTCCCGATCTCAAGTCTAATAGTATCTCCAGCAATTCCCACAGTTAAGCTGCAGGAT

TTCACCAGAGACTTATTAAACCGGCTACGGCCGCTTTAAGCAAGGGCGAATTCTGCAGAT

ATCCATCACACTGGCGGCCGCTCGAGCATGCATCTAGAGGGCCCAATTCGCCCTATAGTG

AGTCGTATTACAATTCACTGGCCGTCGTTTTACAACGTCGTGACTGGGAAAACCCTGGCG

TTACCCAACTTAATCGCCTTGCAGCACATCCCCCTTTCGCCAGCTGGCGTAATAGCGAAG

AGGCCCGCACCGATCGCCCTTCCCAACAGTTGCGCAGCCTGAATGGCGAATGGACGCGCC

CTGTAGCGGCGCATTAAGCGCGGCGGGTGTGGTGGTTACGCGCAGCGTGACCGCTACACT

TGCCAGCGCCCTAGCGCCCGCTCCTTTCGCTTTCTTCCCTTCCTTCTCGCCACGTTCGCC

GGCTTTCCCCGTCAAGCTCTAAATCGGGGGGCTCCCTTTTAGGGTTCCGAATTA

Healthy Control #3

HC3.1 for

CATCGCCCGCAATTTAGGTGACACTATAGAATACTCAAGCTATGCATCAAGCTTGGTACC

GAGCTCGGATCCACTAGTAACGGCCGCCAGTGTGCTGGAATTCGCCCTTGCTTAAAGCGG

CCGTAGCCGGTTTGATAAGTCTTTGGTGAAAGCTTGTAGCTTAACTATAAGAATTGCTGA

AGATACTGTCAGACTTGAAGTCGGGAGAGGTTAGAGGTACTACCGGGGTAGGGGTGAAAT

CCTATAATCCTGGGAGGACCACCTGTGGCGAAGGCGTCTAACTGGAACGATCTTGACGGT

GAGTAACGAAAGCCAGGGGCGCGAACCGGATTAGATACCCGGGTAGTCCTGGCCGTAAAC

GATGTGGACTTGGTGTTGGAATGGCCTCGAGTTGTTCCGGTGCCGAAGGGAAGCTGTTAA

GTCCACCGCCTGGGAAGTACGGTCGCAAGACTGAAACTTAAAGGAATTGGCGGGGGAGCA

CCACAACGCGTGGAGCCTGCGGTTTAATTGGATTCAACGCCAGAAGGGCGAATTCTGCAG

ATATCCATCACACTGGCGGCCGCTCGAGCATGCATCTAGAGGGCCCAATTCGCCCTATAG

TGAGTCGTATTACAATTCACTGGCCGTCGTTTTACAACGTCGTGACTGGGAAAACCCTGG

CGTTACCCAACTTAATCGCCTTGCAGCACATCCCCCTTTCGCCAGCTGGCGTAATAGCGA

AGAGGCCCGCACCGATCGCCCTTCCCAACAGTTGCGCAGCCTGAATGGCGAATGGACGCG

CCCTGTAGCGGCGCATTAAGCGCGGCGGGTGTGGTGGTTACGCGCAGCGTGACCGCTACA

CTTGGCCAGCGCCCTAGCGCCCCGCTCCTTTCGCTTTCTTCCCTTTCCTTTCTCGCCCCG

TTCGCCCGGC

HC3.1 rev

AGCATCCGACTCTATAGGGCGAATTGGGCCCTCTAGATGCATGCTCGAGCGGCCGCCAGT

GTGATGGATATCTGCAGAATTCGCCCTTCTGGCGTTGAATCCAATTAAACCGCAGGCTCC

ACGCGTTGTGGTGCTCCCCCGCCAATTCCTTTAAGTTTCAGTCTTGCGACCGTACTTCCC

AGGCGGTGGACTTAACAGCTTCCCTTCGGCACCGGAACAACTCGAGGCCATTCCAACACC

AAGTCCACATCGTTTACGGCCAGGACTACCCGGGTATCTAATCCGGTTCGCGCCCCTGGC

TTTCGTTACTCACCGTCAAGATCGTTCCAGTTAGACGCCTTCGCCACAGGTGGTCCTCCC

AGGATTATAGGATTTCACCCCTACCCCGGTAGTACCTCTAACCTCTCCCGACTTCAAGTC

TGACAGTATCTTCAGCAATTCTTATAGTTAAGCTACAAGCTTTCACCAAAGACTTATCAA

ACCGGCTACGGCCGCTTTAAGCAAGGGCGAATTCCAGCACACTGGCGGCCGTTACTAGTG

GATCCGAGCTCGGTACCAAGCTTGATGCATAGCTTGAGTATTCTATAGTGTCACCTAAAT

AGCTTGGCGTAATCATGGTCATAGCTGTTTCCTGTGTGAAATTGTTATCCGCTCACAATT

CCACACAACATACGAGCCGGAAGCATAAAGTGTAAAGCCTGGGGTGCCTAATGAGTGAGC

TAACTCACATTAATTGCGTTGCGCTCACTGCCCGCTTTCCAGTCGGGAAACCTGTCGTGC

CAGCTGCATTAATGAATCGGCCAACGCGCGGGGAGAGGCGGTTTGCGTATTGGGCGCTCT

TCCGCTTCCTCGCTCACTGACTCGCTGCGCTCGGTCGTTCGGCTGGCGGCGAGCGGTATC

AGCCTCACCTCAAAGGCGGTTAA

HC3.2 for

TAACGCCGCTTTTAGGTGACACTATAGAATACTCAAGCTATGCATCAAGCTTGGTACCGA

GCTCGGATCCACTAGTAACGGCCGCCAGTGTGCTGGAATTCGCCCTTGCTTAAAGCGGCC

GTAGCCGGTTTGATAAGTCTTTGGTGAAAGCTTGTAGCTTAACTATAAGAATTGCTGAAG

ATACTGTCAGACTTGAAGTCGGGAGAGGTTAGAGGTACTACCGGGGTAGGGGTGAAATCC

TATAATCCTGGGAGGACCACCTGTGGCGAAGGCGTCTAACTGGAACGATCTTGACGGTGA

GTAACGAAAGCCAGGGGCGCGAACCGGATTAGATACCCGGGTAGTCCTGGCCGTAAACGA

TGTGGACTTGGTGTTGGAATGGCCTCGAGTTGTTCCGGTGCCGAAGGGAAGCTGTTAAGT

CCACCGCCTGGGAAGTACGGTCGCAAGACTGAAACTTAAAGGAATTGGCGGGGGAGCACC

ACAACGCGTGGAGCCTGCGGTTTAATTGGATTCAACGCCAGAAGGGCGAATTCTGCAGAT

ATCCATCACACTGGCGGCCGCTCGAGCATGCATCTAGAGGGCCCAATTCGCCCTATAGTG

AGTCGTATTACAATTCACTGGCCGTCGTTTTACAACGTCGTGACTGGGAAAACCCTGGCG

TTACCCAACTTAATCGCCTTGCAGCACATCCCCCTTTCGCCAGCTGGCGTAATAGCGAAG

AGGCCCGCACCGATCGCCCTTCCCAACAGTTGCGCAGCCCTGAATGGCGAATGGACGCGC

CCTGTAGCGGCGCATTAAGCGCGGCGGGTGTGGTGGTTACGCGCAGCGTGACCGCTACAA

CTTGCCAGCGCCCTAGCGCCCGCTCCCTTTCGCTTTCTTCCTTTCCTTTTCTCGCCACGT

TCCGCC

HC3.2 rev

ATTATCGACTCTATAGGGCGAATTGGGCCCTCTAGATGCATGCTCGAGCGGCCGCCAGTG

TGATGGATATCTGCAGAATTCGCCCTTCTGGCGTTGAATCCAATTAAACCGCAGGCTCCA

CGCGTTGTGGTGCTCCCCCGCCAATTCCTTTAAGTTTCAGTCTTGCGACCGTACTTCCCA

GGCGGTGGACTTAACAGCTTCCCTTCGGCACCGGAACAACTCGAGGCCATTCCAACACCA

AGTCCACATCGTTTACGGCCAGGACTACCCGGGTATCTAATCCGGTTCGCGCCCCTGGCT

TTCGTTACTCACCGTCAAGATCGTTCCAGTTAGACGCCTTCGCCACAGGTGGTCCTCCCA

GGATTATAGGATTTCACCCCTACCCCGGTAGTACCTCTAACCTCTCCCGACTTCAAGTCT

GACAGTATCTTCAGCAATTCTTATAGTTAAGCTACAAGCTTTCACCAAAGACTTATCAAA

CCGGCTACGGCCGCTTTAAGCAAGGGCGAATTCCAGCACACTGGCGGCCGTTACTAGTGG

ATCCGAGCTCGGTACCAAGCTTGATGCATAGCTTGAGTATTCTATAGTGTCACCTAAATA

GCTTGGCGTAATCATGGTCATAGCTGTTTCCTGTGTGAAATTGTTATCCGCTCACAATTC

CACACAACATACGAGCCGGAAGCATAAAGTGTAAAGCCTGGGGTGCCTAATGAGTGAGCT

AACTCACATTAATTGCGTTGCGCTCACTGCCCGCTTTCCAGTCGGGAAACCTGTCGTGCC

AGCTGCATTAATGAATCGGCCAACGCGCGGGGAGAGGCGGTTTGCGTATTGGGCGCTCTT

CCGCTTCCTCGCTCACTGACTCGCTGCCGCTCGGTCGTTCGGCTGCG

Healthy Control #5

HC5.1 for

TTTATCCGACCTCTATAGGGCGAATTGGGCCCTCTAGATGCATGCTCGAGCGGCCGCCAG

TGTGATGGATATCTGCAGAATTCGCCCTTGCCTAAAGCGGCCGTAGCCGGTCTGGTACAT

TCGTGGGTAAATCAACCAGCTCAACTGGTTGAATTCTGCGAGCACGGCCAGACTTGGGAC

CGGGAGAGGTGTGGGGTACTCTCAGGGTAGGGGTAAAATCCTGTCATCCTGAGAGGACCA

CCTGTTGCGAAGGCGCCACACTAGAACGGATCCGACGGTCAGGGACGAAGCCTAGGGGCA

CGAACCGGATTAGATACCCGGGTAGTCCTAGGTGTAAACGCTGTGGACTTGATGTTGGGA

GCGCTCCGAGCGCCCTCAATGTCGAAGCGAAGGTGATAAGTCCACTGCCTGGGGAGTACG

GTCGCAAGGCTGAAACTTAAAGGAATTGGCGGGGGAGCACCGCAACCTGAGGATTGTGCG

GTTTAATTGGATTCAACGCCGGAAGGGCGAATTCCAGCACACTGGCGGCCGTTACTAGTG

GATCCGAGCTCGGTACCAAGCTTGATGCATAGCTTGAGTATTCTATAGTGTCACCTAAAT

AGCTTGGCGTAATCATGGTCATAGCTGTTTCCTGTGTGAAATTGTTATCCGCTCACAATT

CCACACAACATACGAGCCGGAAGCATAAAGTGTAAAGCCTGGGGTGCCTAATGAGTGAGC

TAACTCACATTAATTGCGTTGCGCTCACTGCCCGCTTTCCAGTCGGGAAACCTGTCGTGC

CAGCTGCATTAATGAATCGGCCAACGCGCGGGGAGAGGCGGTTTGCGTATTGGCGCTCTT

CCGCTTTCCTCGCTCACTGACTCGCTGCGCTCGGTCGTTCGGCTGCGGCGAGCGGTATC

HC5.1 rev

TACCGCCCGACTTTTAGGTGACACTATAGAATACTCAAGCTATGCATCAAGCTTGGTACC

GAGCTCGGATCCACTAGTAACGGCCGCCAGTGTGCTGGAATTCGCCCTTCCGGCGTTGAA

TCCAATTAAACCGCACAATCCTCAGGTTGCGGTGCTCCCCCGCCAATTCCTTTAAGTTTC

AGCCTTGCGACCGTACTCCCCAGGCAGTGGACTTATCACCTTCGCTTCGACATTGAGGGC

GCTCGGAGCGCTCCCAACATCAAGTCCACAGCGTTTACACCTAGGACTACCCGGGTATCT

AATCCGGTTCGTGCCCCTAGGCTTCGTCCCTGACCGTCGGATCCGTTCTAGTGTGGCGCC

TTCGCAACAGGTGGTCCTCTCAGGATGACAGGATTTTACCCCTACCCTGAGAGTACCCCA

CACCTCTCCCGGTCCCAAGTCTGGCCGTGCTCGCAGAATTCAACCAGTTGAGCTGGTTGA

TTTACCCACGAATGTACCAGACCGGCTACGGCCGCTTTAGGCAAGGGCGAATTCTGCAGA

TATCCATCACACTGGCGGCCGCTCGAGCATGCATCTAGAGGGCCCAATTCGCCCTATAGT

GAGTCGTATTACAATTCACTGGCCGTCGTTTTACAACGTCGTGACTGGGAAAACCCTGGC

GTTACCCAACTTAATCGCCTTGCAGCACATCCCCCTTTCGCCAGCTGGCGTAATAGCGAA

GAGGCCCGCACCGATCGCCCTTCCCAACAGTTGCGCAGCCTGAATGGCGAATGGACGCGC

CCTGTAGCGGCGCATTAAGCGCGGCGGGTGTGGTGGTTACGCGCAGCGTGACCGCTACAC

TTGCCAGCGCCCTAGCGCCCGCTCCTTTCGCTTTCTTCCCTTCCTTTCTCGCCCCGTTCG

GCGGGCTTTCCCGGTCAAGCTCTA

HC5.2 for

AGGATACGAACTCTATAGGGCGAATTGGGCCCTCTAGATGCATGCTCGAGCGGCCGCCAG

TGTGATGGATATCTGCAGAATTCGCCCTTGCTTAAAGGGGCCGTAGCCGGTCTGGTACAT

TCGTGGGTAAATCAACCAGCTCAACTGGTTGAATTCTGCGAGCACGGCCAGACTTGGGAC

CGGGAGAGGTGTGGGGTACTCTCAGGGTAGGGGTAAAATCCTGTCATCCTGAGAGGACCA

CCTGTTGCGAAGGCGCCACACTAGTACGGATCCGACGGTCAGGGACGAAGCCTAGGGGCA

CGAACCGGATTAGATACCCGGGTAGTCCTAGGTGTAAACGCTGTGGACTTGATGTTGGGA

GCGCTCCGAGCGCCCTCAATGTCGAAGCGAAGGTGATAAGTCCACTGCCTGGGGAGTACG

GTCGCAAGGCTGAAACTTAAAGGAATTGGCGGGGGAGCACCGCAACCTGAGGATTGTGCG

GTTTAATTGGATTCAACGCCTGAAGGGCGAATTCCAGCACACTGGCGGCCGTTACTAGTG

GATCCGAGCTCGGTACCAAGCTTGATGCATAGCTTGAGTATTCTATAGTGTCACCTAAAT

AGCTTGGCGTAATCATGGTCATAGCTGTTTCCTGTGTGAAATTGTTATCCGCTCACAATT

CCACACAACATACGAGCCGGAAGCATAAAGTGTAAAGCCTGGGGTGCCTAATGAGTGAGC

TAACTCACATTAATTGCGTTGCGCTCACTGCCCGCTTTCCAGTCGGGAAACCTGTCGTGC

CAGCTGCATTAATGAATCGGCCAACGCGCGGGAGAGGCGGTTTGCGTATTGGGCGCTCTT

CCGCTTCCTCGCTCACTGACTCCGCTGCGCTCGGTCGTTCGGCTGCGGCGAG

HC5.2 rev

TACGCCCGCTTTTAGGTGACACTATAGAATACTCAAGCTATGCATCAAGCTTGGTACCGA

GCTCGGATCCACTAGTAACGGCCGCCAGTGTGCTGGAATTCGCCCTTCAGGCGTTGAATC

CAATTAAACCGCACAATCCTCAGGTTGCGGTGCTCCCCCGCCAATTCCTTTAAGTTTCAG

CCTTGCGACCGTACTCCCCAGGCAGTGGACTTATCACCTTCGCTTCGACATTGAGGGCGC

TCGGAGCGCTCCCAACATCAAGTCCACAGCGTTTACACCTAGGACTACCCGGGTATCTAA

TCCGGTTCGTGCCCCTAGGCTTCGTCCCTGACCGTCGGATCCGTACTAGTGTGGCGCCTT

CGCAACAGGTGGTCCTCTCAGGATGACAGGATTTTACCCCTACCCTGAGAGTACCCCACA

CCTCTCCCGGTCCCAAGTCTGGCCGTGCTCGCAGAATTCAACCAGTTGAGCTGGTTGATT

TACCCACGAATGTACCAGACCGGCTACGGCCCCTTTAAGCAAGGGCGAATTCTGCAGATA

TCCATCACACTGGCGGCCGCTCGAGCATGCATCTAGAGGGCCCAATTCGCCCTATAGTGA

GTCGTATTACAATTCACTGGCCGTCGTTTTACAACGTCGTGACTGGGAAAACCCTGGCGT

TACCCAACTTAATCGCCTTGCAGCACATCCCCCTTTCGCCAGCTGGCGTAATAGCGAAGA

GGCCCGCACCGATCGCCCTTCCCAACAGTTGCGCAGCCTGAATGGCGAATGGACGCGCCC

TGTAGCGGCGCATTAAGCGCGGCGGGTGTGGTGGTTACGCGCAGCGTGACCGCTACACTT

GCCAGCGCCCTAGCGCCCGCTCCTTTCGCTTTCTTCCCTTCCTTTCTTCGCAA

HC5.3 for

TTGATCGACTCTATAGGGCGAATTGGGCCCTCTAGATGCATGCTCGAGCGGCCGCCAGTG

TGATGGATATCTGCAGAATTCGCCCTTGCCTAAAGCGGCCGTAGCCGGTCTGGTACATTC

GTGGGTAAATCAACCAGCTCAACTGGTTGAATTCTGCGAGCACGGCCAGACTTGGGACCG

GGAGAGGTGTGGGGTACTCTCAGGGTAGGGGTAAAATCCTGTCATCCTGAGAGGACCACC

TGTTGCGAAGGCGCCACACTAGAACGGATCCGACGGTCAGGGACGAAGCCTAGGGGCACG

AACCGGATTAGATACCCGGGTAGTCCTAGGTGTAAACGCTGTGGACTTGATGTTGGGAGC

GCTCCGAGCGCCCTCAATGTCGAAGCGAAGGTGATAAGTCCACTGCCTGGGGAGTACGGT

CGCAAGGCTGAAACTTAAAGGAATTGGCGGGGGAGCACCGCAACCTGAGGATTGTGCGGT

TTAATTGGATTCAACGCCAGAAGGGCGAATTCCAGCACACTGGCGGCCGTTACTAGTGGA

TCCGAGCTCGGTACCAAGCTTGATGCATAGCTTGAGTATTCTATAGTGTCACCTAAATAG

CTTGGCGTAATCATGGTCATAGCTGTTTCCTGTGTGAAATTGTTATCCGCTCACAATTCC

ACACAACATACGAGCCGGAAGCATAAAGTGTAAAGCCTGGGGTGCCTAATGAGTGAGCTA

ACTCACATTAATTGCGTTGCGCTCACTGCCCGCTTTCCAGTCGGGAAACCTGTCGTGCCA

GCTGCATTAATGAATCGGCCAACGCGCGGGGAGAGGCGGTTTGCGTATTGGGCGCTCTTC

CGCTTCCTCGCTCACTGACTCGCTGCGCTCGGTCGTTCGGCTGCGGCGAGCGGTTATCAA

GCTTCAC

HC5.3 rev

TACGGCCCACTATTTAGGTGACACTATAGAATACTCAAGCTATGCATCAAGCTTGGTACC

GAGCTCGGATCCACTAGTAACGGCCGCCAGTGTGCTGGAATTCGCCCTTCTGGCGTTGAA

TCCAATTAAACCGCACAATCCTCAGGTTGCGGTGCTCCCCCGCCAATTCCTTTAAGTTTC

AGCCTTGCGACCGTACTCCCCAGGCAGTGGACTTATCACCTTCGCTTCGACATTGAGGGC

GCTCGGAGCGCTCCCAACATCAAGTCCACAGCGTTTACACCTAGGACTACCCGGGTATCT

AATCCGGTTCGTGCCCCTAGGCTTCGTCCCTGACCGTCGGATCCGTTCTAGTGTGGCGCC

TTCGCAACAGGTGGTCCTCTCAGGATGACAGGATTTTACCCCTACCCTGAGAGTACCCCA

CACCTCTCCCGGTCCCAAGTCTGGCCGTGCTCGCAGAATTCAACCAGTTGAGCTGGTTGA

TTTACCCACGAATGTACCAGACCGGCTACGGCCGCTTTAGGCAAGGGCGAATTCTGCAGA

TATCCATCACACTGGCGGCCGCTCGAGCATGCATCTAGAGGGCCCAATTCGCCCTATAGT

GAGTCGTATTACAATTCACTGGCCGTCGTTTTACAACGTCGTGACTGGGAAAACCCTGGC

GTTACCCAACTTAATCGCCTTGCAGCACATCCCCCTTTCGCCAGCTGGCGTAATAGCGAA

GAGGCCCGCACCGATCGCCCTTCCCAACAGTTGCGCAGCCTGAATGGCGAATGGACGCGC

CCTGTAGCGGCGCATTAAGCGCGCGGGTGTGGTGGTTACGCGCAGCGTGACCGCTACACT

TGCCAGCGCCCTAGCCGCCGCTCCTTTCGCTTTTCTTCCCTTCCCTTTCTCGCCCACG

HC5.4 for

TGAATTCGACCTCCTATAGGGCGAATTGGGCCCTCTAGATGCATGCTCGAGCGGCCGCCA

GTGTGATGGATATCTGCAGAATTCGCCCTTCTGGCGTTGAATCCAATTAAACCGCACAAT

CCTCAGGTTGCGGTGCTCCCCCGCCAATTCCTTTAAGTTTCAGCCTTGCGACCGTACTCC

CCAGGCAGTGGACTTATCACCTTCGCTTCGACATTGAGGGCGCTCGGAGCGCTCCCAACA

TCAAGTCCACAGCGTTTACACCTAGGACTACCCGGGTATCTAATCCGGTTCGTGCCCCTA

GGCTTCGTCCCTGACCGTCGGATCCGTTCTAGTGTGGCGCCTTCGCAACAGGTGGTCCTC

TCAGGATGACAGGATTTTACCCCTACCCTGAGAGTACCCCACACCTCTCCCGGTCCCAAG

CCTGGCCGTGCTCGCAGAATTCAACCAGTTGAGCTGGTTGATTTACCCACGAATGTACCA

GACCGGCTACGGCCGCTTTAGGCAAGGGCGAATTCCAGCACACTGGCGGCCGTTACTAGT

GGATCCGAGCTCGGTACCAAGCTTGATGCATAGCTTGAGTATTCTATAGTGTCACCTAAA

TAGCTTGGCGTAATCATGGTCATAGCTGTTTCCTGTGTGAAATTGTTATCCGCTCACAAT

TCCACACAACATACGAGCCGGAAGCATAAAGTGTAAAGCCTGGGGTGCCTAATGAGTGAG

CTAACTCACATTAATTGCGTTGCGCTCACTGCCCGCTTTCCAGTCGGGAAACCTGTCGTG

CCAGCTGCATTAATGAATCGGCCAACGCGCGGGGAGAGGCGGTTTGCGTATTGGCGCTCT

TCCGCTTCCTCGCTCACTGACTCCGCTGCGCTCGGTCGTTCGGCTGCGGCGGGGCGGTTA

TCCAGCT

HC5.4 rev

TTTCGGCCCGGCCATTTAGGTGACACTATAGAATACTCAAGCTATGCATCAAGCTTGGTA

CCGAGCTCGGATCCACTAGTAACGGCCGCCAGTGTGCTGGAATTCGCCCTTGCCTAAAGC

GGCCGTAGCCGGTCTGGTACATTCGTGGGTAAATCAACCAGCTCAACTGGTTGAATTCTG

CGAGCACGGCCAGGCTTGGGACCGGGAGAGGTGTGGGGTACTCTCAGGGTAGGGGTAAAA

TCCTGTCATCCTGAGAGGACCACCTGTTGCGAAGGCGCCACACTAGAACGGATCCGACGG

TCAGGGACGAAGCCTAGGGGCACGAACCGGATTAGATACCCGGGTAGTCCTAGGTGTAAA

CGCTGTGGACTTGATGTTGGGAGCGCTCCGAGCGCCCTCAATGTCGAAGCGAAGGTGATA

AGTCCACTGCCTGGGGAGTACGGTCGCAAGGCTGAAACTTAAAGGAATTGGCGGGGGAGC

ACCGCAACCTGAGGATTGTGCGGTTTAATTGGATTCAACGCCAGAAGGGCGAATTCTGCA

GATATCCATCACACTGGCGGCCGCTCGAGCATGCATCTAGAGGGCCCAATTCGCCCTATA

GTGAGTCGTATTACAATTCACTGGCCGTCGTTTTACAACGTCGTGACTGGGAAAACCCTG

GCGTTACCCAACTTAATCGCCTTGCAGCACATCCCCCTTTCGCCAGCTGGCGTAATAGCG

AAGAGGCCCGCACCGATCGCCCTTCCCAACAGTTGCGCAGCCTGAATGGCGAATGGACGC

GCCTGTAGCGGCGCATAAAGCGCGGCGGGTGTGGTGGTTACGCGCAGCCGTGACCGCTAC

ACTTTGCCAGCGCCCT

Ulcerative Colitis Inflamed #1

UC Infl.6.4 for

AGGAACCCGAACTACTATAGGGCGAATTGGGCCCTCTAGATGCAAGCTCGAGCGGCCGCC

AGTGTGATGGATATCTGCAGAATTCGCCCTTGCCTAAAGCGGCCGTAGCCGGTTTAATAA

GTCTCTGGTGAAATCCTGCAGCTTAACTGTGGGAATTGCTGGAGATACTATTAGGCTTGA

GATCGGGAGAGGTTAGAGGTACTC

UC Infl.6.4 rev

GCCGCCCACCTTTTAGGTGACACTATAGAATACTCAAGCTATGCATCAAGCTTGGTACCG

AGCTCGGATCCACTAGTAACGGCCGCCAGTGTGCTGGAATTCGCCCTTCAGGCGTTGAAT

CCAATTAAACCGCAGGCTCCACGCGTTGTGGTGCTCCCCCGCCAATTCCTTTAAGTTTCA

GTCTTGCGACCGTACTTCCCAGGCGGCGGACTTAACAGCTTCCCTTCGGCACTGGGACAG

CTCAAAGCCACCCCAACACCAAGTCCGCATCGTTTACAGCTAGGACTACCCGGGTATCTA

ATCCGGTTCGCGCCCCTAGCTTTCGTCCCTCACCGTCAGAATCGTTCCAGTCAGACGCCT

TCGCAACAGGCGGTCCTCCCAGGATTACAGAATTTCACCTCTACCCTGGGAGTACCTCTA

ACCTCTCCCGATCTCAAGCCTAATAGTATCTCCAGCAATTCCCACAGTTAAGCTGCAGGA

TTTCACCAGAGACTTATTAAACCGGCTACGGCCGCTTTAGGCAAGGGCGAATTCTGCAGA

TATCCATCACACTGGCGGCCGCTCGAGCATGCATCTAGAGGGCCCAATTCGCCCTATAGT

GAGTCGTATTACAATTCACTGGCCGTCGTTTTACAACGTCGTGACTGGGAAAACCCTGGC

GTTACCCAACTTAATCGCCTTGCAGCACATCCCCCTTTCGCCAGCTGGCGTAATAGCGAA

GAGGCCCGCACCGATCGCCCTTCCCAACAGTTGCGCAGCCTGAATGGCGAATGGACGCGC

CCTGTAGCGGCGCATTAAGCGCGGCGGGTGTGGTGGTTACGCGCAGCGTGACCCGCTACA

CTTGCCAGCGCCCTAGCGCCCGCTCCTTTCGCCTTTCTTCCCTTTCCTTTCTCGCCACGG

TTCG

Ulcerative Colitis Inflamed #2

UC Infl.7.1 for

AGTAAACCGACTACTATAGGGCGAATTGGGCCCTCTAGATGCATGCTCGAGCGGCCGCCA

GTGTGATGGATATCTGCAGAATTCGCCCTTGCCTAAAGGGGCCGTAGCCGGTTTAATAAG

TCTCTGGTGAAATCCTGCAGCTTAACTGTGGGAATTGCTGGAGATACTATTAGACTTGAG

ATCGGG

UC Infl.7.1 rev

TACCGCCGCTTTTAGGTGACACTATAGAATACTCAAGCTATGCATCAAGCTTGGTACCGA

GCTCGGATCCACTAGTAACGGCCGCCAGTGTGCTGGAATTCGCCCTTCTGGCGTTGAATC

CAATTAAACCGCAGGCTCCACGCGTTGTGGTGCTCCCCCGCCAATTCCTTTAAGTTTCAG

TCTTGCGACCGTACTTCCCAGGCGGCGGACTTAACAGCTTCCCTTCGGCACTGGGACAGC

TCAAAGCCACCCCAACACCAAGTCCGCATCGTTTACAGCTAGGACTACCCGGGTATCTAA

TCCGGTTCGCGCCCCTAGCTTTCGTCCCTCACCGTCAGAATCGTTCCAGTCAGACGCCTT

CGCAACAGGCGGTCCTCCCAGGATTACAGAATTTCACCTCTACCCTGGGAGTACCTCTAA

CCTCTCCCGATCTCAAGTCTAATAGTATCTCCAGCAATTCCCACAGTTAAGCTGCAGGAT

TTCACCAGAGACTTATTAAACCGGCTACGGCCCCTTTAGGCAAGGGCGAATTCTGCAGAT

ATCCATCACACTGGCGGCCGCTCGAGCATGCATCTAGAGGGCCCAATTCGCCCTATAGTG

AGTCGTATTACAATTCACTGGCCGTCGTTTTACAACGTCGTGACTGGGAAAACCCTGGCG

TTACCCAACTTAATCGCCTTGCAGCACATCCCCCTTTCGCCAGCTGGCGTAATAGCGAAG

AGGCCCGCACCGATCGCCCTTCCCAACAGTTGCGCAGCCTGAATGGCGAATGGACGCGCC

CTGTAGCGGCGCATTAAGCGCGGCGGGTGTGGTGGTTACGCGCAGCGTGACCGCTACACT

TGCCAGCGCCCTAGCGCCCGCTCCTTTCGCTTTCTTCCCTTCCTTTTCTCGCCCCGTTCG

G

UC Infl.7.2 for

TGTATCCGACTACTATAGGGCGAATTGGGCCCTCTAGATGCATGCTCGAGCGGCCGCCAG

TGTGATGGATATCTGCAGAATTCGCCCTTCTGGCGTTGAATCCAATTAAACCGCAGGCTA

CACGCGTTGTGGTGCTCCCCCGCCAATTCCTTTAAGTTTCAGTCTTGCGACCGTACTTCC

CAGGCGGCGGACTTAACAGCTTCCCTTCGGCACTGGGACAGCTCAAAGCCACCCCAACAC

CAAGTCCGCATCGTTTACTGCTAGGACTACCCGGGTATCTAATCCGGTTCGCGCCCCTAG

CTTTCGTCCCTCACCGTCAGAATCGTTCCAGTCAGACGCCTTCGCAACAGGCGGTCCTCC

CAGGATTACAGAATTTCACCTCTACCCTGGGAGTACCTCTAACCTCTCCCGATCTCAAGT

CTAACAGTATCTCCAGCAATTCCCACAGTTAAGCTGCAGGATTTCACCAGAGACTTATTA

AACCGGCTACGGCCGCTTTAGGCAAGGGCGAATTCCAGCACACTGGCGGCCGTTACTAGT

GGATCCGAGCTCGGTACCAAGCTTGATGCATAGCTTGAGTATTCTATAGTGTCACCTAAA

TAGCTTGGCGTAATCATGGTCATAGCTGTTTCCTGTGTGAAATTGTTATCCGCTCACAAT

TCCACACAACATACGAGCCGGAAGCATAAAGTGTAAAGCCTGGGGTGCCTAATGAGTGAG

CTAACTCACATTAATTGCGTTGCGCTCACTGCCCGCTTTCCAGTCGGGAAACCTGTCGTG

CCAGCTGCATTAATGAATCGGCCAACGCGCGGGGAGAGGCGGGTTTGCGTATTGGGCGCT

CTTCCGCTTCCTCGCTCACTGACTCGCTGCGCTCGGTCGTTCGGCTGCGGCGAGC

UC Infl.7.2 rev

ATGCCCAACTATTTAGGTGACACTATAGAATACTCAAGCTATGCATCAAGCTTGGTACCG

AGCTCGGATCCACTAGTAACGGCCGCCAGTGTGCTGGAATTCGCCCTTGCCTAAAGCGGC

CGTAGCCGGTTTAATAAGTCTCTGGTGAAATCCTGCAGCTTAACTGTGGGAATTGCTGGA

GATACTGTTAGACTTGAGATCGGGAGAGGTTAGAGGTACTCCCAGGGTAGAGGTGAAATT

CTGTAATCCTGGGAGGACCGCCTGTTGCGAAGGCGTCTGACTGGAACGATTCTGACGGTG

AGGGACGAAAGCTAGGGGCGCGAACCGGATTAGATACCCGGGTAGTCCTAGCAGTAAACG

ATGCGGACTTGGTGTTGGGGTGGCTTTGAGCTGTCCCAGTGCCGAAGGGAAGCTGTTAAG

TCCGCCGCCTGGGAAGTACGGTCGCAAGACTGAAACTTAAAGGAATTGGCGGGGGAGCAC

CACAACGCGTGTAGCCTGCGGTTTAATTGGATTCAACGCCAGAAGGGCGAATTCTGCAGA

TATCCATCACACTGGCGGCCGCTCGACCATGCATCTAGAGGGCCCAATTTTGCCCTATAG

TGAGTCGTATTACAATTCACTGGCCGTCCTTTTACAACGTCGTGACTGGGAAAAACCCTG

GCGTACCCAACTTACATCGCCCT

UC Infl.7.3 for

TTGAAACCGACCTCACTATAGGGCGAATTGGGCCCTCTAGATGCATGCTCGAGCGGCCGC

CAGTGTGATGGATATCTGCAGAATTCGCCCTTGCCTAAAGCGGCCGTAGCCGGTTTGATA

AGTCTTTGGTGAAAGCTTGTAGCTTAACTATAAGAATTGCTGAAGATACTGTCAGACTTG

AAGTCGGGAGAGGTTGGAGGTACTACCGGGGTAGGGGTGAA

UC Infl.7.3 rev

TACCGCCAACTATTTAGGTGACACTATAGAATACTCAAGCTATGCATCAAGCTTGGTACC

GAGCTCGGATCCACTAGTAACGGCCGCCAGTGTGCTGGAATTCGCCCTTCTGGCGTTGAA

TCCAATTAAACCGCAGGCTCCACGCGTTGTGGTGCTCCCCCGCCAATTCCTTTAAGTTTC

AGTCTTGCGACCGTACTTCCCAGGCGGTGGACTTAACAGCTTCCCTTCGGCACCGGAACA

ACTCGAGGCCATTCCAACACCAAGTCCACATCGTTTACGGCCAGGACTACCCGGGTATCT

AATCCGGTTCGCGCCCCTGGCTTTCGTTACTCACCGTCAAGATCGTTCCAGTTAGACGCC

TTCGCCACAGGTGGTCCTCCCAGGATTATAGGATTTCACCCCTACCCCGGTAGTACCTCC

AACCTCTCCCGACTTCAAGTCTGACAGTATCTTCAGCAATTCTTATAGTTAAGCTACAAG

CTTTCACCAAAGACTTATCAAACCGGCTACGGCCGCTTTAGGCAAGGGCGAATTCTGCAG

ATATCCATCACACTGGCGGCCGCTCGAGCATGCATCTAGAGGGCCCAATTCGCCCTATAG

TGAGTCGTATTACAATTCACTGGCCGTCGTTTTACAACGTCGTGACTGGGAAAACCCTGG

CGTTACCCAACTTAATCGCCTTGCAGCACATCCCCCTTTCGCCAGCTGGCGTAATAGCGA

AGAGGCCCGCACCGATCGCCCTTCCCAACAGTTGCGCAGCCTGAATGGCGAATGGACGCG

CCCCTGTAGCGGCGCATTAAGCGCGGCGGGTGTGGTGGTTACGCGCAGCGTGACCCGCTA

CACTTGCCAGCGCCCTAGCGCCCGCTCCTTTCGCTTTTCTTCCCTTCCTTTTCTCGCCCA

CGTTCGGC

Ulcerative Colitis Inflamed #4

UC Infl.9.1 for

TGTATACCGACCTCTATAGGGCGAATTGGGCCCTCTAGATGCATGCTCGAGCGGCCGCCA

GTGTGATGGATATCTGCAGAATTCGCCCTTGCTTAAAGCGGCCGTAGCCGGTTTGATAAG

TCTTTGGTGAAAGCTTGTAGCTTAACTATAAGAATTGCTGAAGATACTGTCAGACTTGAA

GTCGGGAGAGGTTAGAGGTACTACCGGGGTAGGGGTGAAATCCTATAATCCTGGGAGGAC

CACCCGTGGCGAAGGCGTCTAACTGGAACGATCTTGACGGTGAGTAACGAAAGCCAGGGG

CGCGAACCGGATTAGATACCCGGGTAGTCCTGGCCGTAAACGATGTGGACTTGGTGTTGG

AGTGGCCTCGAGTTGTTCCGGTGCCGAAGGGAAGCTGTTAAGTCCACCGCCTGGGAAGTA

CGGTCGCAAGACTGAAACTTAAAGGAATTGGCGGGGGAGCACCACAACGCGTGGAGCCTG

CGGTTTAATTGGATTCAACGCCTGAAGGGCGAATTCCAGCACACTGGCGGCCGTTACTAG

TGGATCCGAGCTCGGTACCAAGCTTGATGCATAGCTTGAGTATTCTATAGTGTCACCTAA

ATAGCTTGGCGTAATCATGGTCATAGCTGTTTCCTGTGTGAAATTGTTATCCGCTCACAA

TTCCACACAACATACGAGCCGGAAGCATAAAGTGTAAAGCCTGGGGTGCCTAATGAGTGA

GCTAACTCACATTAATTGCGTTGCGCTCACTGCCCGCTTTCCAGTCCGGAAACCTGTCGT

GCCAGCTGCATTAATGAATCGGCCAACGCGCGGGGAGAGGCGGGTTTGCGTATTGGGCGC

TTCTTCCGCTTCCTCGCTCACTGACTCGCTGCGCTCGGTCGTTCGGCTGCGGCGAAGCGG

TATCAGCTTACTTC

UC Infl.9.1 rev

TCCCCCCCGCTTTTAGGTGACACTATAGAATACTCAAGCTATGCATCAAGCTTGGTACCG

AGCTCGGATCCACTAGTAACGGCCGCCAGTGTGCTGGAATTCGCCCTTCAGGCGTTGAAT

CCAATTAAACCGCAGGCTCCACGCGTTGTGGTGCTCCCCCGCCAATTCCTTTAAGTTTCA

GTCTTGCGACCGTACTTCCCAGGCGGTGGACTTAACAGCTTCCCTTCGGCACCGGAACAA

CTCGAGGCCACTCCAACACCAAGTCCACATCGTTTACGGCCAGGACTACCCGGGTATCTA

ATCCGGTTCGCGCCCCTGGCTTTCGTTACTCACCGTCAAGATCGTTCCAGTTAGACGCCT

TCGCCACGGGTGGTCCTCCCAGGATTATAGGATTTCACCCCTACCCCGGTAGTACCTCTA

ACCTCTCCCGACTTCAAGTCTGACAGTATCTTCAGCAATTCTTATAGTTAAGCTACAAGC

TTTCACCAAAGACTTATCAAACCGGCTACGGCCGCTTTAAGCAAGGGCGAATTCTGCAGA

TATCCATCACACTGGCGGCCGCTCGAGCATGCATCTAGAGGGCCCAATTCGCCCTATAGT

GAGTCGTATTACAATTCACTGGCCGTCGTTTTACAACGTCGTGACTGGGAAAACCCTGGC

GTTACCCAACTTAATCGCCTTGCAGCACATCCCCCTTTCGCCAGCTGGCGTAATAGCGAA

GAGGCCCCGCACCGATCGCCCTTCCCAACAGTTGCGCAGCCTGAATGGCGAATGGACGCG

CCCTGTAGCGGCGCATTAAGCGCGGCGGGTGTGGTGGTTACGCGCAGCGTGACCGCTACA

ACTTGCCAGCGCCCTAGCGCCCGCTCCTTTCGCTTTCTTCCCTTTCCTTTCTCGCCACGT

TCGGCGGGCTTTCCCCGTCAAGCTCTAAATCGGGGGCTCCCCTTTAGG

UC Infl.9.2 for

GTTATCCGACTACTATAGGGCGAATTGGGCCCTCTAGATGCATGCTCGAGCGGCCGCCAG

TGTGATGGATATCTGCAGAATTCGCCCTTGCCTAAAGCGGCCGTAGCCGGTTTGATAAGT

CTTTGGTGAAAGCTTGTAGCTTAACTATAAGAATTGCTGAAGATACTGTCAGAC

UC Infl.9.2 rev

CCGCCTTTTAGGTGACACTATAGAATACTCAAGCTATGCATCAAGCTTGGTACCGAGCTC

GGATCCACTAGTAACGGCCGCCAGTGTGCTGGAATTCGCCCTTCTGGCGTTGAATCCAAT

TAAACCGCAGGCTCCACGCGTTGTGGTGCTCCCCCGCCAATTCCTTTAAGTTTCAGTCTT

GCGACCGTACTTCCCAGGCGGTGGACTTAACAGCTTCCCTTCGGCACCGGAACAACTCGA

GGCCATTCCAACACCAAGTCCACATCGTTTACGGCCAGGACTACCCGGGTATCTAATCCG

GTTCGCACCCCTGGCTTTCGTTACTCACCGTCAAGATCGTTCCAGTTAGACGCCTTCGCC

ACAGGTGGTCCTCCCAGGATTATAGGATTTCACCCCTACCCCGGTAGTACCTCTAACCTC

TCCCGACTTCAAGTCTGACAGTATCTTCAGCAATTCTTATAGTTAAGCTACAAGCTTTCA

CCAAAGACTTATCAAACCGGCTACGGCCGCTTTAGGCAAGGGCGAATTCTGCAGATATCC

ATCACACTGGCGGCCGCTCGAGCATGCATCTAGAGGGCCCAATTCGCCCTATAGTGAGTC

GTATTACAATTCACTGGCCGTCGTTTTACAACGTCGTGACTGGGAAAACCCTGGCGTTAC

CCAACTTAATCGCCTTGCAGCACATCCCCCTTTCGCCAGCTGGCGTAATAGCGAAGAGGC

CCGCACCGATCGCCCTTCCCAACAGTTGCGCAGCCTGAATGGCGAATGGACGCGCCCTGT

AGCGGCGCATTAAGCGCGGCGGGTGTGGTGGTTACGCGCAGCGTGACCGCTACACTTGCC

AGCGCCCTAGCGCCCGCTCCTTTCGCTTTCTTCCCTTTCCTTTTCTCGCCACGTTCGCCG

G

UC Infl.9.3 for

TTTAACGACCTCTATAGGGCGAATTGGGCCCTCTAGATGCATGCTCGAGCGGCCGCCAGT

GTGATGGATATCTGCAGAATTCGCCCTTGCTTAAAGCGGCCGTAGCCGGTTTGATAAGTC

TTTGGTGAAAGCTTGTAGCTTAACTATAAGAATTGCTGAAGATACTGTCAGACTTGAAGT

CGGGAGAGGTTAGAGGTACTACCGGGGTAGGGGTGAAATCCTATAATCCTGGGAGGGCCA

CCTGTGGGGAAGGCGTCTAACTGGAACGATCTTGACGGTGAGTAACGAAAGCCAGGGGCG

CGAACCGGATTAGATACCCGGGTAGTCCTGGCCGTAAACGATGTGGACTTGGTGTTGGAA

TGGCCTCGAGTTGTTCCGGTGCCGAAGGGAAGCTGTTAAGTCCACCGCCTGGGAAGTACG

GTCGCAAGACTGAAACTTAAAGGAATTAGCGGGGGAGCACCACAACGCGTGGAGCCTGCG

GTTTAATTGGATTCAACGCCTGAAGGGCGAATTCCAGCACACTGGCGGCCGTTACTAGTG

GATCCGAGCTCGGTACCAAGCTTGATGCATAGCTTGAGTATTCTATAGTGTCACCTAAAT

AGCTTGGCGTAATCATGGTCATAGCTGTTTCCTGTGTGAAATTGTTATCCGCTCACAATT

CCACACAACATACGAGCCGGAAGCATAAAGTGTAAAGCCTGGGGGTGCCTAATGAGTGAG

CTAACTCACATTAATTGCGTTGCGCTCACTGCCCGCTTTCCAGTCGGGAAACCTGTCGTG

CCAGCTGCATTAATGAATCGGCCAACGCGCGGGGAGAGGCGGGTTTGCGTATTGGGCGCT

CTTCCGCTTCCTCGCTCACTGACTCGCTGCGCTCGGTCGTTCGGCTGCGGC

UC Infl.9.3 rev

TATGGCCCCACCATTTAGGTGACACTATAGAATACTCAAGCTATGCATCAAGCTTGGTAC

CGAGCTCGGATCCACTAGTAACGGCCGCCAGTGTGCTGGAATTCGCCCTTCAGGCGTTGA

ATCCAATTAAACCGCAGGCTCCACGCGTTGTGGTGCTCCCCCGCTAATTCCTTTAAGTTT

CAGTCTTGCGACCGTACTTCCCAGGCGGTGGACTTAACAGCTTCCCTTCGGCACCGGAAC

AACTCGAGGCCATTCCAACACCAAGTCCACATCGTTTACGGCCAGGACTACCCGGGTATC

TAATCCGGTTCGCGCCCCTGGCTTTCGTTACTCACCGTCAAGATCGTTCCAGTTAGACGC

CTTCCCCACAGGTGGCCCTCCCAGGATTATAGGATTTCACCCCTACCCCGGTAGTACCTC

TAACCTCTCCCGACTTCAAGTCTGACAGTATCTTCAGCAATTCTTATAGTTAAGCTACAA

GCTTTCACCAAAGACTTATCAAACCGGCTACGGCCGCTTTAAGCAAGGGCGAATTCTGCA

GATATCCATCACACTGGCGGCCGCTCGAGCATGCATCTAGAGGGCCCAATTCGCCCTATA

GTGAGTCGTATTACAATTCACTGGCCGTCGTTTTACAACGTCGTGACTGGGAAAACCCTG

GCGTTACCCAACTTAATCGCCTTGCAGCACATCCCCCTTTCGCCAGCTGGCGTAATAGCG

AAGAGGCCCGCACCGATCGCCCTTCCCAACAGTTGCGCAGCCTGAATGGCGAATGGACGC

GCCCTGTAGCGGCGCATTAAGCGCGGCGGGTGTGGTGGTTACGCGCAGCGTGACCGCTAC

ACTTGCCAGCGCCCTAGCGCCCGCTCCTTTCGCTTTCTTCCCTTTCCTTTCT

Ulcerative Colitis Uninflamed #2

UC Uninfl.12.1 for

AGTAATCCGACCTACTATAGGGCGAATTGGGCCCTCTAGTATGCATGCTCGAGCGGCCGC

CAGTGTGATGGATATCTGCAGAATTCGCCCTTCCGGCGTTGAATCCAATTAAACCGCAGG

CTCCACGCGTTGTGGTGCTCCCCCGCCAATTCCTTTAAGTTTCAGTCTTGCGACCGTACT

TCCCAGGCGGTGGACTTAACAGCTTCCCTTCGGCACCGGAACAACTCGAGGCCATTCCAA

CACCAAGTCCACATCGTTTACAGCCAGGACTACCCGGGTATCTAATCCGGTTCGCGCCCC

TGGCTTTCGTTACTCACCGTCAAGATCGTTCCAGTTAGACGCCTTCGCCACAGGTGGTCC

TCCCAGGATTATAGGATTTCACCCCTACCCCGGTAGTACCTCTAACCTCTCCCGACTTCA

AGTCTGACAGTATCTTCAGCAATTCTTATAGTTAAGCTACAAGCTTTCACCAAAGACTTA

TCAAACCGGCTACGGCCGCTTTAGGCAAGGGCGAATTCCAGCACACTGGCGGCCGTTACT

AGTGGATCCGAGCTCGGTACCAAGCTTGATGCATAGCTTGAGTATTCTATAGTGTCACCT

AAATAGCTTGGCGTAATCATGGTCATAGCTGTTTCCTGTGTGAAATTGTTATCCGCTCAC

AATTCCACACAACATACGAGCCGGAAGCATAAAGTGTAAAGCCTGGGGGTGCCTAATGAG

TGAGCTAACTCACATTAATTGCGTTGCGCTCACTGCCCGCTTTCCAGTCGGGAAACCTGT

CGTGCCAGCTGCATTAATGAATCGGCCACGCGCGGGAGAGGCGGTTTGCGTATTGGGCGC

TCTTCCGCTTCCTCGCTCACTGACTCGCTGCGCTCGGTCGTTCGGCTGCGGCGAGCGGTA

TCAGCTCACTCCAAGGCGGGTAAATACGGGTTATTC

UC Uninfl.12.1 rev

GACCGCCCGCTTTTTAGGTGACACTATAGAATACTCAAGCTATGCATCAAGCTTGGTACC

GAGCTCGGATCCACTAGTAACGGCCGCCAGTGTGCTGGAATTCGCCCTTGCCTAAAGCGG

CCGTAGCCGGTTTGATAAGTCTTTGGTGAAAGCTTGTAGCTTAACTATAAGAATTGCTGA

AGATACTGTCAGACTTGAAGTCGGGAGAGGTTAGAGGTACTACCGGGGTAGGGGTGAAAT

Ulcerative Colitis Uninflamed #3

UC Uninfl.13.1 for

TACGCCGCTTTTAGGTGACACTATAGAATACTCAAGCTATGCATCAAGCTTGGTACCGAG

CTCGGATCCACTAGTAACGGCCGCCAGTGTGCTGGAATTCGCCCTTCAGGCGTTGAATCC

AATTAAACCGCAGGCTCCACGCGTTGTGGTGCTCCCCCGCCAATTCCTTTAAGTTTCAGT

CTTGCGACCGTACTTCCCAGGCGGTGGACTTAACAGCTTCCCTTCGGCACCGGAACAACT

CGAGGCCATTCCAACACCAAGTCCACATCGTTTACGGCCAGGACTACCCGGGTATCTAAT

CCGGTTCGCGCCCCTGGCTTTCGATACTCACCGTCAAGATCGTTCCAGTTAGACGCCTTC

GCCACAGGTGGTCCTCCCAGGATTATAAGATTTCACCCCTACCCCGGTAGTACCTCTAAC

CTCTCCCGACTTCAAGTCTGACAGTATCTTCAGCAATTCTTATAGTTAAGCTACAAGCTT

TCACCAAAGACTTATCAAACCGGCTACGGCCGCTTTAGGCAAGGGCGAATTCTGCAGATA

TCCATCACACTGGCGGCCGCTCGAGCATGCATCTAGAGGGCCCAATTCGCCCTATAGTGA

GTCGTATTACAATTCACTGGCCGTCGTTTTACAACGTCGTGACTGGGAAAACCCTGGCGT

TACCCAACTTAATCGCCTTGCAGCACATCCCCCTTTCGCCAGCTGGCGTAATAGCGAAGA

GGCCCGCACCGATCGCCCTTCCCAACAGTTGCGCAGCCTGAATGGCGAATGGACGCGCCC

TGTAGCGGCGCATTAAGCGCGCGGGTGTGGTGGTTACGCGCAGCGTGACCGCTACACTTG

CCAGCGCCCTAGCGCCCGCTCCTTTCGCTTTTCTTCCCTTTCCTTTTCTCCGCCACGTTC

CGCCGGCTTT

UC Uninfl.13.1 rev

AGTAAACCGACTCTATAGGGCGAATTGGGCCCTCTAGATGCATGCTCGAGCGGCCGCCAG

TGTGATGGATATCTGCAGAATTCGCCCTTGCCTAAAGCGGCCGTAGCCGGTTTGATAAGT

CTTTGGTGAAAGCTTGTAGCTTAACTATAAGAATTGCTGAAGATACTGTCAGACTTGAAG

TCGGGAGAGGTTAGAGGTACTACCGGGGTAGGGGTGAAATCTTATAATCCTGGGAGGACC

ACCTGTGGCGAAGGCGTCTAACTGGAACGATCTTGACGGTGAGTATCGAAAGCCAGGGGC

GCGAACCGGATTAGATACCCGGGTAGTCCTGGCCGTAAACGATGTGGACTTGGTGTTGGA

ATGGCCTCGAGTTGTTCCGGTGCCGAAGGGAAGCTGTTAAGTCCACCGCCTGGGAAGTAC

GGTCGCAAGACTGAAACTTAAAGGAATTGGCGGGGGAGCACCACAACGCGTGGAGCCTGC

GGTTTAATTGGATTCAACGCCTGAAGGGCGAATTCCAGCACACTGGCGGCCGTTACTAGT

GGATCCGAGCTCGGTACCAAGCTTGATGCATAGCTTGAGTATTCTATAGTGTCACCTAAA

TAGCTTGGCGTAATCATGGTCATAGCTGTTTCCTGTGTGAAATTGTTATCCGCTCACAAT

TCCACACAACATACGAGCCGGAAGCATAAAGTGTAAAGCCTGGGGTGCCTAATGAGTGAG

CTAACTCACATTAATTGCGTTGCGCTCACTGCCCGCTTTCCAGTCGGGAAACCTGTCGTG

CCAGCTGCATTAATGAATCGGCCAACGCGCGGGGAGAGGCGGTTTGCGTATTGGGCGCTC

TTCCGCTTCCTCGCTCACTGACTCGCTGCGCTCGGTCGTTCGGCTGCGGCGAGGCGGTAT

TCAGCTTCACCTCAAAGGCGGTT

UC Uninfl.13.2 for

TACCCACGCCTATTTAGGTGACACTATAGAATACTCAAGCTATGCATCAAGCTTGGTACC

GAGCTCGGATCCACTAGTAACGGCCGCCAGTGTGCTGGAATTCGCCCTTCAGGCGTTGAA

TCCAATTAAACCGCAGGCTCCACGCGTTGTGGTGCTCCCCCGCCAATTCCTTTAAGTTTC

AGTCTTGCGACCGTACTTCCCAGGCGGCGGACTTAACAGCTTCCCTTCGGCACTGGGACA

GCTCAAAGCCACCCCAACACCAAGTCCGCATCGTTTACAGCTAGGACTACCCGGGTATCT

AATCCGGTTCGCGCCCCTAGCTTTCGTCCCTCACCGTCAGAATCGTTCCAGTCAGACGCC

TTCGCAACAGGCGGTCCTCCCAGGATTACAGAATTTCACCTCTACCCTGGGAGTACCTCT

AACCTCTCCCGATCTCAAGTCTAATAGTATCTCCAGCAATTCCCACAGTTAAGCTGCAGG

ATTTCACCAGAGACTTATTAAACCGGCTACGGCCGCTTTAAGCAAGGGCGAATTCTGCAG

ATATCCATCACACTGGCGGCCGCTCGAGCATGCATCTAGAGGGCCCAATTCGCCCTATAG

TGAGTCGTATTACAATTCACTGGCCGTCGTTTTACAACGTCGTGACTGGGAAAACCCTGG

CGTTACCCAACTTAATCGCCTTGCAGCACATCCCCCTTTCGCCAGCTGGCGTAATAGCGA

AGAGGCCCGCACCGATCGCCCTTCCCAACAGTTGCGCAGCCTGAATGGCGAATGGACGCG

CCCTGTAGCGGCGCATTAAGCGCGCGGGTGTGGTGGTTACGCGCAGCGTGACCGCTACAC

TTGCCAGCGCCTAGCGCCGCTCTTTCGCTTTCTTCCTTCCTTTCTCGGCACGGTTCCGCC

GGCTTCCCGTCAAGCTCTTAAATCCGGGGCCTCCCTTTAG

UC Uninfl.13.2 rev

AGGTATCGACTCCTATAGGGCGAATTGGGCCCTCTAGATGCATGCTCGAGCGGCCGCCAG

TGTGATGGATATCTGCAGAATTCGCCCTTGCTTAAAGCGGCCGTAGCCGGTTTAATAAGT

CTCTGGTGAAATCCTGCAGCTTAACTGTGGGAATTGCTGGAGATACTATTAGACTTGAGA

TCGGGAGAGGTTAGAGGTACTCCCAGGGTAGAGGTGAAATTCTGTAATCCTGGGAGGACC

GCCTGTTGCGAAGGCGTCTGACTGGAACGATTCTGACGGTGAGGGACGAAAGCTAGGGGC

GCGAACCGGATTAGATACCCGGGTAGTCCTAGCTGTAAACGATGCGGACTTGGTGTTGGG

GTGGCTTTGAGCTGTCCCAGTGCCGAAGGGAAGCTGTTAAGTCCGCCGCCTGGGAAGTAC

GGTCGCAAGACTGAAACTTAAAGGAATTGGCGGGGGAGCACCACAACGCGTGGAGCCTGC

GGTTTAATTGGATTCAACGCCTGAAGGGCGAATTCCAGCACACTGGCGGCCGTTACTAGT

GGATCCGAGCTCGGTACCAAGCTTGATGCATAGCTTGAGTATTCTATAGTGTCACCTAAA

TAGCTTGGCGTAATCATGGTCATAGCTGTTTCCTGTGTGAAATTGTTATCCGCTCACAAT

TCCACACAACATACGAGCCGGAAGCATAAAGTGTAAAGCCTGGGGTGCCTAATGAGTGAG

CTAACTCACATTAATTGCGTTGCGCTCACTGCCGCTTTCCAGTCGGGAAACCTGTCGTGC

CAGCTGCATTAATGAATCGGCCACGCGCGGGGAGAGGCGGTTTGCGTATTGGGCGCTCTC

GCTTCTCCGCTCACTGACTCGCTGCGCTCGTCGTCGGCTGCGGCGAGGCGGTATCAGCCT

CACCTCAAAGGCGGTAATACCGGTA

UC Uninfl.13.3 for

TACGCCCACTATTTAGGTGACACTATAGAATACTCAAGCTATGCATCAAGCTCTGGTACC

GAGCTCGGATCCACTAGTAACGGCCGCCAGTGTGCTGGAATTCGCCCTTCAGGCGTTGAA

TCCAATTAAACCGCAGGCTCCACGCGTTGTGGTGCTCCCCCGCCAATTCCTTTAAGTTTC

AGTCTTGCGACCGTACTTCCCAGGCGGCGGACTTAACAGCTTCCCTTCGGCACTGGGACA

GCTCAAAGCCACCCCGACACCAAGTCCGCATCGTTTACAGCTAGGACTACCCGGGTATCT

AATCCGGTTCGCGCCCCTAGCTTTCGTCCCTCACCGTCAGAATCGTTCCAGTCAGACGCC

TTCGCAACAGGCGGTCCTCCCAGGATTACAGAATTTCACCTCTACCCTGGGAGTACCTCT

AACCTCTCCCGATCTCAAGTCTAATAGTATCTCCAGCAATTCCCACAGTTAAGCTGCAGG

ATTTCGCCAGAGACTTATTAAACCGGCTACGGCCGCTTTAAGCAAGGGCGAATTCTGCAG

ATATCCATCACACTGGCGGCCGCTCGAGCATGCATCTAGAGGGCCCAATTCGCCCTATAG

TGAGTCGTATTACAATTCACTGGCCGTCGTTTTACAACGTCGTGACTGGGAAAACCCTGG

CGTTACCCAACTTAATCGCCTTGCAGCACATCCCCCTTTCGCCAGCTGGCGTAATAGCGA

AGAGGCCCGCACCGATCGCCCTTCCCAACAGTTGCGCAGCCTGAATGGCGAATGGACGCG

CCCTGTAGCGGCGCATTAAGCGCGCGGGTGTGGTGGTTACGCGCAGCGTGACCGCTACAC

TGCCAGCGCCCTAGCGCCCGCTCTTTCGCTTTCTCCTCTTTCTCGCACGTCGCCGGCTTT

CCCGTCAGCTTCTAATCGGGGCTCCTTAGGGTTCCGATTTAGTGCT

UC Uninfl.13.3 rev

CTGAAAACCGACCTCCTATAGGGCGAATTGGGCCCTCTAGATGCATGCTCGAGCGGCCGC

CAGTGTGATGGATATCTGCAGAATTCGCCCTTGCTTAAAGCGGCCGTAGCCGGTTTAATA

AGTCTCTGGCGAAATCCTGCAGCTTAACTGTGGGAATTGCTGGAGATACTATTAGACTTG

AGATCGGGAGAGGTTAGAGGTACTCCCAGGGTAGAGGTGAAATTCTGTAATCCTGGGAGG

ACCGCCTGTTGCGAAGGCGTCTGACTGGAACGATTCTGACGGTGAGGGACGAAAGCTAGG

GGCGCGAACCGGATTAGATACCCGGGTAGTCCTAGCTGTAAACGATGCGGACTTGGTGTC

GGGGTGGCTTTGAGCTGCCCCAGTGCCCAAGGGAAGCTGCTAAGTCCGCCGCCTGCGAAG

TACGGTCGCAAGACTGAATCTTAAAGGAATTGGCGGGGGAGCACCACCCACCGTGGAGCC

TGCGGTTTAATTGGATTCAACGCCTGAAGGGCAAATTCCAGCA

UC Uninfl.13.4 for

TACGGCCCACTTTTAGGTGACACTATAGAATACTCAAGCTATGCATCAAGCTTGGTACCG

AGCTCGGATCCACTAGTAACGGCCGCCAGTGTGCTGGAATTCGCCCTTGCTTAAAGCGGC

CGTAGCCGGTTTGATAAGTCTTTGGTGAAAGCTTGTAGCTTAACTATAAGAATTGCTGAA

GATACTGTCAGACTTGAAGTCGGGAGAGGTTAGAGGTACTACCGGGGTAGGGGTGAAATC

CTATAATCCTGGGAGGACCACCTGTGGCGAAGGCGTCTAACTGGAACGATCTTGACGGTG

AGTAACGAAAGCCAGGGGCGCGAACCGGATTAGATACCCGGGTAGTCCTGGCCGTAAACG

ATGTGGACTTGGTGTTGGAATGGCCTCGAGTTGTTCCGGTGCCGAAGGGAAGCTGTTAAG

TCCACCGCCTGGGAAGTACGGTCGCAAGACTGAAACTTAAAGGAATTGGCGGGGGAGCAC

CACAACGCGTGGAGCCTGCGGTTTAATTGGATTCAACGCCAGAAGGGCGAATTCTGCAGA

TATCCATCACACTGGCGGCCGCTCGAGCATGCATCTAGAGGGCCCAATTCGCCCTATAGT

GAGTCGTATTACAATTCACTGGCCGTCGTTTTACAACGTCGTGACTGGGAAAACCCTGGC

GTTACCCAACTTAATCGCCTTGCAGCACATCCCCCTTTCGCCAGCTGGCGTAATAGCGAA

GAGGCCCGCACCGATCGCCCTTCCCAACAGTTGCGCAGCCTGAATGGCGAATGGACGCGC

CCTGTAGCGGCGCATTAAGCGCGGCGGGTGTGGTGGTTACGCGCAGCGTGACCGCTACAC

TTGCCAGCGCCCTAGCGCCCGCTCCTTTCGCTTTCTTCCTTCTTTCTCGCCACGTTCGCC

GCTTTCCCCGTCAAGCTCTAAATCGGGGCTCCCTTAGGTTTCCGAATAA

UC Uninfl.13.4 rev

AGTATCCGACTCTATAGGGCGAATTGGGCCCTCTAGATGCATGCTCGAGCGGCCGCCAGT

GTGATGGATATCTGCAGAATTCGCCCTTCTGGCGTTGAATCCAATTAAACCGCAGGCTCC

ACGCGTTGTGGTGCTCCCCCGCCAATTCCTTTAAGTTTCAGTCTTGCGACCGTACTTCCC

AGGCGGTGGACTTAACAGCTTCCCTTCGGCACCGGAACAACTCGAGGCCATTCCAACACC

AAGTCCACATCGTTTACGGCCAGGACTACCCGGGTATCTAATCCGGTTCGCGCCCCTGGC

TTTCGTTACTCACCGTCAAGATCGTTCCAGTTAGACGCCTTCGCCACAGGTGGTCCTCCC

AGGATTATAGGATTTCACCCCTACCCCGGTAGTACCTCTAACCTCTCCCGACTTCAAGTC

TGACAGTATCTTCAGCAATTCTTATAGTTAAGCTACAAGCTTTCACCAAAGACTTATCAA

ACCGGCTACGGCCGCTTTAAGCAAGGGCGAATTCCAGCACACTGGCGGCCGTTACTAGTG

GATCCGAGCTCGGTACCAAGCTTGATGCATAGCTTGAGTATTCTATAGTGTCACCTAAAT

AGCTTGGCGTAATCATGGTCATAGCTGTTTCCTGTGTGAAATTGTTATCCGCTCACAATT

CCACACAACATACGAGCCGGAAGCATAAAGTGTAAAGCCTGGGGTGCCTAATGAGTGAGC

TAACTCACATTAATTGCGTTGCGCTCACTGCCCGCTTTCCAGTCGGGAAACCTGTCGTGC

CAGCTGCATTAATGAATCGGCCAACGCGCGGGGAGAGGCGGTTTGCGTATTGGGCGCTCT

TCCGCTTCTCGCTCACTGACTCGCTGCGCTCGGTCGTTCGGCTGCGGCGAGCGGTATCAG

CCTCCACTCAAAGGCGGTAATAACGGTAATCCACAAGAATCAGGGGATAACG

Ulcerative Colitis Uninflamed #5

UC Uninfl.15.1 for

GAGGTAAACCGACCTCCTATAGGGCGAATTGGGCCCTCTAGATGCATGCTCGAGCGGCCG

CCAGTGTGATGGATATCTGCAGAATTCGCCCTTCAGGCGTTGAATCCAATTAAGCCGCAG

GCTCCACGCGTTGTGGTGCTCCCCCGCCAATTCCTTTAAGTTTCAGTCTTGCGACCGTAC

TTCCCAGGCGGCGGACTTAACAGCTTCCCCTCGGCACTGGGACAGCTCAAAGCCACCCCA

ACACCAAGTCCGCATCGTTTACAGCTAGGACTACCCGGGTATCTAATCCGGTTCGCGCCC

CTAGCTTTCGTCCCTCACCGTCAGAATCGTTCCAGTCAGACGCCTTCGCAACAGGCGGTC

CTCCCAGGATTACAGAATTTCACCTCTACCCTGGGAGTACCTCTAACCTCTCCCGATCTC

AAGTCTAATAGTATCTCCAGCAATTCCCACAGTTAAGCTGCAGGATTTCACCAGAGACTT

ATTAAACCGGCTACGGCCGCTTTAAGCAAGGGCGAATTCCAGCACACTGGCGGCCGTTAC

TAGTGGATCCGAGCTCGGTACCAAGCTTGATGCATAGCTTGAGTATTCTATAGTGTCACC

TAAATAGCTTGGCGTAATCATGGTCATAGCTGTTTCCCTGTGTGAAATTGCTATCCGCTC

ACAATTTCCCACAACATACGAGCCGGAAGCATAAAGTGCAAAGCCTGGCGGGGCCTAATG

AGCGAG

UC Uninfl.15.1 rev

TTCCCCCACTTTTAGGTGACACTATAGAATACTCAAGCTATGCATCAAGCTTGGTACCGA

GCTCGGATCCACTAGTAACGGCCGCCAGTGTGCTGGAATTCGCCCTTGCTTAAAGCGGCC

GTAGCCGGTTTAATAAGTCTCTGGTGAAATCCTGCAGCTTAACTGTGGGAATTGCTGGAG

ATACTATTAGACTTGAGATCGGGAGAGGTTAGAGGTACTCCCAGGGTAGAGGTGAAATTC

TGTAATCCTGGGAGGACCGCCTGTTGCGAAGGCGTCTGACTGGAACGATTCTGACGGTGA

GGGACGAAAGCTAGGGGCGCGAACCGGATTAGATACCCGGGTAGTCCTAGCTGTAAACGA

TGCGGACTTGGTGTTGGGGTGGCTTTGAGCTGTCCCAGTGCCGAGGGGAAGCTGTTAAGT

CCGCCGCCTGGGAAGTACGGTCGCAAGACTGAAACTTAAAGGAATTGGCGGGGGAGCACC

ACAACGCGTGGAGCCTGCGGCTTAATTGGATTCAACGCCTGAAGGGCGAATTCTGCAGAT

ATCCATCACACTGGCGGCCGCTCGAGCATGCATCTAGAGGGCCCAATTCGCCCTATAGTG

AGTCGTATTACAATTCACTGGCCGTCGTTTTACAACGTCGTGACTGGGAAAACCCTGGCG

TTACCCAACTTAATCGCCTTGCAGCACATCCCCCTTTCGCCAGCTGGCGTAATAGCGAAG

AGGCCCGCACCGATCGCCCTTCCCAACAGTTGCGCAGCCTGAATGGCGAATGGACGCGCC

CTGTAGCGGCGCATTAAGCGCGGCGGGTGTGGTGGTTACGCGCAGCGTGACCGCTACACT

TGCCAGCGCCCTAGCGCCCGCTCCTTTCGCTTTCTTCCCTTTCCTTTCTCCGCCACGTTC

GGCCGGCC

UC Uninfl.15.2 for

AGTTAACCGACTACTATAGGGCGAATTGGGCCCTCTAGATGCATGCTCGAGCGGCCGCCA

GTGTGATGGATATCTGCAGAATTCGCCCTTGCCTAAAGCGGCCGTAGCCGGTTTAATAAG

TCTCTGGTGAAATCCTGCAGCTTAACTGTGGGAATTGCTGGAGATACTATTAGACTTGAG

ATCGGGAGAGGTTAGAGGTACTCCCAGAGTAGAGGTGAAATTCTGTAATCCTGGGAGGAC

CGCCTGTTGCGAAGGCGTCTGACTGGAACGATTCTGACGGTGAGGGACGAAAGCTAGGGG

CGCGAACCGGATTAGATACCCGGGTAGTCCTAGCTGTAAACGATGCGGACTTGGTGTTGG

GGTGGCTTTGAGCTGTCCCAGTGCCGAAGGGAAGCTGTTAAGTCCGCCGCCTGGGAAGTA

CGGTCGCAAGACTGAAACTTAAAGGAATTGGCGGGGGAGCACCACAACGCGTGGAGCCTG

CGGTTTAATTGGATTCAACGCCTGAAGGGCGAATTCCAGCACACTGGCGGCCGTTACTAG

TGGATCCGAGCTCGGTACCAAGCTTGATGCATAGCTTGAGTATTCTATAGTGTCACCTAA

ATAGCTTGGCGTAATCATGGTCATAGCTGTTTCCTGTGTGAAATTGTTATCCGCTCACAA

TTCCACACAACATACGAGCCGGAAGCATAAAGTGTAAAGCCTGGGGTGCCTAATGAGTGA

GCTAACTCACATTAATTGCGTTGCGCTCACTGCCCGCTTTCCAGTCGGGAAACCTGTCGT

GCCAGCTGCATTAATGAATCGGCCAACGCGCGGGGAGAGGCGGTTTGCGTATTGGGCGCT

CTTCCGCTTCCTCGCTCACTGACTCGCTGCGCTCGGTCGTTCGGCTGCGC

UC Uninfl.15.2 rev

TCCGCCACTTTTAGGTGACACTATAGAATACTCAAGCTATGCATCAAGCTTGGTACCGAG

CTCGGATCCACTAGTAACGGCCGCCAGTGTGCTGGAATTCGCCCTTCAGGCGTTGAATCC

AATTAAACCGCAGGCTCCACGCGTTGTGGTGCTCCCCCGCCAATTCCTTTAAGTTTCAGT

CTTGCGACCGTACTTCCCAGGCGGCGGACTTAACAGCTTCCCTTCGGCACTGGGACAGCT

CAAAGCCACCCCAACACCAAGTCCGCATCGTTTACAGCTAGGACTACCCGGGTATCTAAT

CCGGTTCGCGCCCCTAGCTTTCGTCCCTCACCGTCAGAATCGTTCCAGTCAGACGCCTTC

GCAACAGGCGGTCCTCCCAGGATTACAGAATTTCACCTCTACTCTGGGAGTACCTCTAAC

CTCTCCCGATCTCAAGTCTAATAGTATCTCCAGCAATTCCCACAGTTAAGCTGCAGGATT

TCACCAGAGACTTATTAAACCGGCTACGGCCGCTTTAGGCAAGGGCGAATTCTGCAGATA

TCCATCACACTGGCGGCCGCTCGAGCATGCATCTAGAGGGCCCAATTCGCCCTATAGTGA

GTCGTATTACAATTCACTGGCCGTCGTTTTACAACGTCGTGACTGGGAAAACCCTGGCGT

TACCCAACTTAATCGCCTTGCAGCACATCCCCCTTTCGCCAGCTGGCGTAATAGCGAAGA

GGCCCGCACCGATCGCCCTTCCCAACAGTTGCGCAGCCTGAATGGCGAATGGACGCGCCC

TGTAGCGGCGCATTAAGCGCGGCGGGTGTGGTGGTTACGCGCAGCGTGACCGCTACACTT

GCCAGCGCCCTAGCGCCCGCTCCTTTCGCTTTCTTCCCTTTCCTTTCTCCGCCACGTTCC

GCCGGCTTTCCCCGTCAAGCT

UC Uninfl.15.3 for

TTACCGACCTACTATAGGGCGAATTGGGCCCTCTAGATGCATGCTCGAGCGGCCGCCAGT

GTGATGGATATCTGCAGAATTCGCCCTTGCTTAAAGGGGCCGTAGCCGGTTTAATAAGTC

TCTGGTGAAATCCTGCAGCTTAACTGTGGGAGTTGCTGGAGATACTATTAGACTTGAGAT

CGGGAGAGGTTAGAGGTACTCCCAGGGTAGAGGTGAAATTCTGTAATCCTGGGAGGACCG

CCTGTTGCGAAGGCGTCTGACTGGAACGATTCTGACGGTGAGGGACGAAAGCTAGGGGCG

CGAACCGGATTAGATACCCGGGTAGTCCTAGCTGTAAACGATGCGGACTTGGTGTTGGGG

TGGCTTTGAGCTGTCCCAGTGCCGAAGGGAAGCTGTTAAGTCCGCCGCCTGGGAAGTACG

GTCGCAAGACTGAAACTTAAAGGAATTGGCGGGGGAGCACCACAACGCGTGGAGCCTGCG

GTTTAATTGGATTCAACGCCTGAAGGGCGAATTCCAGCACACTGGCGGCCGTTACTAGTG

GATCCGAGCTCGGTACCAAGCTTGATGCATAGCTTGAGTATTCTATAGTGTCACCTAAAT

AGCTTGGCGTAATCATGGTCATAGCTGTTTCCTGTGTGAAATTGTTATCCGCTCACAATT

CCACACAACATACGAGCCGGAAGCATAAAGTGTAAAGCCTGGGGTGCCTAATGAGTGAGC

TAACTCACATTAATTGCGTTGCGCTCACTGCCCGCTTTCCAGTCGGGAAACCTGTCGTGC

CAGCTGCATTAATGAATCGGCCAACGCGCGGGGAGAGGCGGTTTGCGTATTGGGCGCTCT

TTCCGCTTCCTCGCTCACCTGACTCGCTGCGCTCGGTCGTTCGGCTGGCGGCGAGCCGGT

ATCA

UC Uninfl.15.3 rev

CCCCCGCCCATTTAGGTGACACTATAGAATACTCAAGCTATGCATCAAGCTTGGTACCGA

GCTCGGATCCACTAGTAACGGCCGCCAGTGTGCTGGAATTCGCCCTTCAGGCGTTGAATC

CAATTAAACCGCAGGCTCCACGCGTTGTGGTGCTCCCCCGCCAATTCCTTTAAGTTTCAG

TCTTGCGACCGTACTTCCCAGGCGGCGGACTTAACAGCTTCCCTTCGGCACTGGGACAGC

TCAAAGCCACCCCAACACCAAGTCCGCATCGTTTACAGCTAGGACTACCCGGGTATCTAA

TCCGGTTCGCGCCCCTAGCTTTCGTCCCTCACCGTCAGAATCGTTCCAGTCAGACGCCTT

CGCAACAGGCGGTCCTCCCAGGATTACAGAATTTCACCTCTACCCTGGGAGTACCTCTAA

CCTCTCCCGATCTCAAGTCTAATAGTATCTCCAGCAACTCCCACAGTTAAGCTGCAGGAT

TTCACCAGAGACTTATTAAACCGGCTACGGCCCCTTTAAGCAAGGGCGAATTCTGCAGAT

ATCCATCACACTGGCGGCCGCTCGAGCATGCATCTAGAGGGCCCAATTCGCCCTATAGTG

AGTCGTATTACAATTCACTGGCCGTCGTTTTACAACGTCGTGACTGGGAAAACCCTGGCG

TTACCCAACTTAATCGCCTTGCAGCACATCCCCCTTTCGCCAGCTGGCGTAATAGCGAAG

AGGCCCGCACCGATCGCCCTTCCCAACAGTTGCGCAGCCTGAATGGCGAATGGACGCGCC

CTGTAGCGGCGCATTAAGCGCGGCGGGTGTGGTGGTTACGCGCAGCGTGACCGCTACACT

TGCCAGCGCCCTAGCGCCCGCTCCTTTCGCTTTTCTTCCCTTCCTTTCTTCGCCACGGTT

CGC

UC Uninfl.15.4 for

AGTAAACGACTACTATAGGGCGAATTGGGCCCTCTAGATGCATGCTCGAGCGGCCGCCAG

TGTGATGGATATCTGCAGAATTCGCCCTTCAGGCGTTGAATCCAATTAAACCGCAGGCTC

CACGCGTTGTGGTGCTCCCCCGCCAATTCCTTTAAGTTTCAGTCTTGCGACCGTACTTCC

CAGGCGGCGGACTTAACAGCTTCCCTTCGGCACTGGGACAGCTCAAAGCCACCCCAACAC

CAAGTCCGCATCGTTTACAGCTAGGACTACCCGGGTATCTAATCCGGTTCGCGCCCCTAG

CTTTCGTCCCTCACCGTCAGAATCGTTCCAGTCAGACGCCTTCGCAACAGGCGGTCCTCC

CAGGATTACAGAATTTCACCTCTACCCTGGGAGTACCTCTAACCTCTCCCGATCTCAAGT

CTAATAGTATCTCCAGCAATTCCCACAGTTAAGCTGCAGGATTTCACCAGAGACTTATTA

AACCGGCTACGGCCCCTTTAAGCAAGGGCGAATTCCAGCACACTGGCGGCCGTTACTAGT

GGATCCGAGCTCGGTACCAAGCTTGATGCATAGCTTGAGTATTCTATAGTGTCACCTAAA

TAGCTTGGCGTAATCATGGTCATAGCTGTTTCCTGTGTGAAATTGTTATCCGCTCACAAT

TCCACACAACATACGAGCCGGAAGCATAAAGTGTAAAGCCTGGGGTGCCTAATGAGTGAG

CTAACTCACATTAATTGCGTTGCGCTCACTGCCCGCTTTCCAGTCGGGAAACCTGTCGTG

CCAGCTGCATTAATGAATCGGCCAACGCGCGGGGAGAGGCGGTTTGCGTATTGGGCGCTC

TTCCGCTTCCTCGCTCACTGACTCGCTGCGCTCCG

UC Uninfl.15.4 rev

GAACGCCGCTTTTAGGTGACACTATAGAATACTCAAGCTATGCATCAAGCTTGGTACCGA

GCTCGGATCCACTAGTAACGGCCGCCAGTGTGCTGGAATTCGCCCTTGCTTAAAGGGGCC

GTAGCCGGTTTAATAAGTCTCTGGTGAAATCCTGCAGCTTAACTGTGGGAATTGCTGGAG

ATACTATTAGACTTGAGATCGGGAGAGGTTAGAGGTACTCCCAGGGTAGAGGTGAAATTC

TGTAATCCTGGGAGGACCGCCTGTTGCGAAGGCGTCTGACTGGAACGATTCTGACGGTGA

GGGACGAAAGCTAGGGGCGCGAACCGGATTAGATACCCGGGTAGTCCTAGCTGTAAACGA

TGCGGACTTGGTGTTGGGGTGGCTTTGAGCTGTCCCAGTGCCGAAGGGAAGCTGTTAAGT

CCGCCGCCTGGGAAGTACGGTCGCAAGACTGAAACTTAAAGGAATTGGCGGGGGAGCACC

ACAACGCGTGGAGCCTGCGGTTTAATTGGATTCAACGCCTGAAGGGCGAATTCTGCAGAT

ATCCATCACACTGGCGGCCGCTCGAGCATGCATCTAGAGGGCCCAATTCGCCCTATAGTG

AGTCGTATTACAATTCACTGGCCGTCGTTTTACAACGTCGTGACTGGGAAAACCCTGGCG

TTACCCAACTTAATCGCCTTGCAGCACATCCCCCTTTCGCCAGCTGGCGTAATAGCGAAG

AGGCCCGCACCGATCGCCCTTCCCAACAGTTGCGCAGCCTGAATGGCGAATGGACGCGCC

CTGTAGCGGCGCATTAAGCGCGGCGGGTGTGGTGGTTACGCGCAGCGTGACCGCTACAAC

TTGCCAGCGCCCTAGCGCCCGCTCCTTTCGCTT

Crohn´s Disease Inflamed #1

CD Infl.16.1 for

TGAATCCGACTACCTATAGGGCGAATTGGGCCCTCTAGATGCATGCTCGAGCGGCCGCCA

GTGTGATGGATATCTGCAGAATTCGCCCTTGCTTAAAGCGGCCGTAGCCGGTTTAACAAG

TCTCTGGTGAAATCCTGCAGCTCAACTGTGGGAATTGCTGGAGATACTATTAGACTTGAG

ATCGGGAGAGGTTAGAGGTACTCCCAGGGTAGAGGTGAAATTCTGTAATCCTGGGAGGAC

CGCCTGTTGCGAAGGCGTCTGACTGGAACGATTCTGACGGTGAGGGACGAAAGCTAGGGG

CGCGAACCGGATTAGATACCCGGGTAGTCCTAGCTGTAAACGATGCGGACTTGGTGTTGG

GGTGGCTTTGAGCTGTCCCAGTGCCGAAGGGAAGCTGTTAAGTCCGCCGCCTGGGAAGTA

CGGTCGCAAGACTGAAACTTAAAGGAATTGGCGGGGGAGCACCACAACGCGTGGAGCCTG

CGGTTTAATTGGATTCAACGCCTGAAGGGCGAATTCCAGCACACTGGCGGCCGTTACTAG

TGGATCCGAGCTCGGTACCAAGCTTGATGCATAGCTTGAGTATTCTATAGTGTCACCTAA

ATAGCTTGGCGTAATCATGGTCATAGCTGTTTCCTGTGTGAAATTGTTATCCGCTCACAA

TTCCACACAACATACGAGCCGGAAGCATAAAGTGTAAAGCCTGGGGTGCCTAATGAGTGA

GCTAACTCACATTAATTGCGTTGCGCTCACTGCCCGCTTTCCAGTCGGGAAACCTGTCGT

GCCAGCTGCATTAATGAATCGGCCAACGCGCGGGGAGAGGCGGTTTGCGTATTGGGCGCT

CTTCCGCTTCCTCGCTCACTGACTCGCTGCGCTCGGTCGTTCGGCTGCGCGAAGCGGTAT

CAGCTCACTCAAAGGGCGGTAATAACGGTTATCCACAGATTCA

CD Infl.16.1 rev

TACGCCCACACATTTAGGTGACACTATAGAATACTCAAGCTATGCATCAAGCTTGGTACC

GAGCTCGGATCCACTAGTAACGGCCGCCAGTGTGCTGGAATTCGCCCTTCAGGCGTTGAA

TCCAATTAAACCGCAGGCTCCACGCGTTGTGGTGCTCCCCCGCCAATTCCTTTAAGTTTC

AGTCTTGCGACCGTACTTCCCAGGCGGCGGACTTAACAGCTTCCCTTCGGCACTGGGACA

GCTCAAAGCCACCCCAACACCAAGTCCGCATCGTTTACAGCTAGGACTACCCGGGTATCT

AATCCGGTTCGCGCCCCTAGCTTTCGTCCCTCACCGTCAGAATCGTTCCAGTCAGACGCC

TTCGCAACAGGCGGTCCTCCCAGGATTACAGAATTTCACCTCTACCCTGGGAGTACCTCT

AACCTCTCCCGATCTCAAGTCTAATAGTATCTCCAGCAATTCCCACAGTTGAGCTGCAGG

ATTTCACCAGAGACTTGTTAAACCGGCTACGGCCGCTTTAAGCAAGGGCGAATTCTGCAG

ATATCCATCACACTGGCGGCCGCTCGAGCATGCATCTAGAGGGCCCAATTCGCCCTATAG

TGAGTCGTATTACAATTCACTGGCCGTCGTTTTACAACGTCGTGACTGGGAAAACCCTGG

CGTTACCCAACTTAATCGCCTTGCAGCACATCCCCCTTTCGCCAGCTGGCGTAATAGCGA

AGAGGCCCGCACCGATCGCCCTTCCCAACAGTTGCGCAGCCTGAATGGCGAATGGACGCG

CCCTGTAGCGGCGCATTAAGCGCGGCGGGTGTGGTGGTTACGCGCAGCGTGACCGCTACA

CTTGCCAGCGCCCTAGCGCCCGCTCCTTTCGCTTTCTTCCCTTCTTTCTCGCCACGTTTC

GCCGCTTTCCCCGTCAGCTTCTAAATCGGGGGCTCCTTTTAGGGTTCCGATTTAATGCTT

TAACGGCAACT

CD Infl.16.2 for

TGTATCGACTACTATAGGGCGAATTGGGCCCTCTAGATGCATGCTCGAGCGGCCGCCAGT

GTGATGGATATCTGCAGAATTCGCCCTTGCTTAAAGGGGCCGTAGCCGGTTTAATAAGTC

TCTGGTGAAATCCTGCAGCTTAACTGTGGGAATTGCTGGAGATACTATTAGACTTGAGAT

CGGGAGAGGTTAGAGGTACTCCCAGGGTAGAGGTGAAATTCTGTAATCCTGGGAGGACCG

CCTGTTGCGAAGGCGTCTGACTGGGACGATTCTGACGGTGAGGGACGAAAGCTAGGGGCG

CGAACCGGATTAGATACCCGGGTAGTCCTAGCTGTAAACGATGCGGACTTGGTGTTGGGG

TGGCTTTGAGCTGTCCCAGTGCCGAAGGGAAGCTGTTAAGTCCGCCGCCTGGGAAGTACG

GTCGCAAGACTGAAACTTAAAGGAATTGGCGGGGGAGCACCACAACGCGTGGAGCCTGCG

GTTTAATTGGATTCAACGCCTGAAGGGCGAATTCCAGCACACTGGCGGCCGTTACTAGTG

GATCCGAGCTCGGTACCAAGCTTGATGCATAGCTTGAGTATTCTATAGTGTCACCTAAAT

AGCTTGGCGTAATCATGGTCATAGCTGTTTCCTGTGTGAAATTGTTATCCGCTCACAATT

CCACACAACATACGAGCCGGAAGCATAAAGTGTAAAGCCTGGGGGTGCCTAATGAGTGAG

CTAACTCACATTAATTGCGTTGCGCTCACTGCCCGCTTTCCAGTCGGGAAACCTGTCGTG

CCAGCTGCATTAATGAATCGGCCAACGCGCGGGGAGAGGCGGTTTGCGTATTGGGCGCTC

TTCCGCTTCCTCGCTCACTGACTCGCTGCGCTCGGTCGTTCGGCTGCGGCGAGCGTATCA

GCTCACTCAAAGGCGGTAATACGGTTATCCACAGGATCAGGGGATAACGCCAGGAAAGAA

ACATCGT

CD Infl.16.2 rev

TACGCCCGCTTTTAGGTGACACTATAGAATACTCAAGCTATGCATCAAGCTTGGTACCGA

GCTCGGATCCACTAGTAACGGCCGCCAGTGTGCTGGAATTCGCCCTTCAGGCGTTGAATC

CAATTAAACCGCAGGCTCCACGCGTTGTGGTGCTCCCCCGCCAATTCCTTTAAGTTTCAG

TCTTGCGACCGTACTTCCCAGGCGGCGGACTTAACAGCTTCCCTTCGGCACTGGGACAGC

TCAAAGCCACCCCAACACCAAGTCCGCATCGTTTACAGCTAGGACTACCCGGGTATCTAA

TCCGGTTCGCGCCCCTAGCTTTCGTCCCTCACCGTCAGAATCGTCCCAGTCAGACGCCTT

CGCAACAGGCGGTCCTCCCAGGATTACAGAATTTCACCTCTACCCTGGGAGTACCTCTAA

CCTCTCCCGATCTCAAGTCTAATAGTATCTCCAGCAATTCCCACAGTTAAGCTGCAGGAT

TTCACCAGAGACTTATTAAACCGGCTACGGCCCCTTTAAGCAAGGGCGAATTCTGCAGAT

ATCCATCACACTGGCGGCCGCTCGAGCATGCATCTAGAGGGCCCAATTCGCCCTATAGTG

AGTCGTATTACAATTCACTGGCCGTCGTTTTACAACGTCGTGACTGGGAAAACCCTGGCG

TTACCCAACTTAATCGCCTTGCAGCACATCCCCCTTTCGCCAGCTGGCGTAATAGCGAAG

AGGCCCGCACCGATCGCCCTTCCCAACAGTTGCGCAGCCTGAATGGCGAATGGACGCGCC

CTGTAGCGGCGCATTAAGCGCGGCGGGTGTGGTGGTTACGCGCAGCGTGACCGCTACACT

TGCCAGCGCCCTAGCGCCCGCTCCTTTCGCTTTCTTCCCTTCTTTCTCGCCACGTTCGCC

GGCTTTCCCGTCAAGCTCTAATCGGGGGCTCCCTTTAGGGTTTCCGAT

CD Infl.16.3 for

TTTAATCGACTACTATAGGGCGAATTGGGCCCTCTAGATGCATGCTCGAGCGGCCGCCAG

TGTGATGGATATCTGCAGAATTCGCCCTTGCCTAAAGCGGCCGTAGCCGGTTTAATAAGT

CTCTGGTGAAATCCTGCAGCTTAACTGTGGGAATTGCTGGAGATACTATTAGACTTGAGA

TCGGGAGAGGTTAGAGGTACTCCCAGGGTTGAGGTGAAATTCTGTAATCCTGGGAGGACC

GCCTGTTGCGAAGGCGTCTGACTTGAACGATTCTGACGGTGAGGGACGAAAGCTAGGGGC

GCGAACCGGATTAGATACCCGGGTAGTCCTAGCTGTAAACGATGCGGACTTGGTGTTGGG

GTGGCTTTGAGCTGTCCCAGTGCCGAAGGGAAGCTGTTAAGTCCGCCGCCTGGGAAGTAC

GGTCGCAAGACTGAAACTTAAAGGAATTGGCGGGGGAGCACCACAACGCGTGGAGCCTGC

GGTTTAATTGGATTCAACGCCTGAAGGGCGAATTCCAGCACACTGGCGGCCGTTACTAGT

GGATCCGAGCTCGGTACCAAGCTTGATGCATAGCTTGAGTATTCTATAGTGTCACCTAAA

TAGCTTGGCGTAATCATGGTCATAGCTGTTTCCTGTGTGAAATTGTTATCCGCTCACAAT

TCCACACAACATACGAGCCGGAAGCATAAAGTGTAAAGCCTGGGGTGCCTAATGAGTGAG

CTAACTCACATTAATTGCGTTGCGCTCACTGCCCGCTTTCCAGTCGGGAAACCTGTCGTG

CCAGCTGCATTAATGAATCGGCCAACGCGCGGGGAGAGGCGGGTTTGCGTATTGGGCGCT

CTCCGCTTCCTCGCTCACTGACTCGCTGCGCTCGGTCGTTCGGCTGCGGCGAGCGGTATC

AGCCTCACTCAAAGGCGGTAATACGGGTTATTCCACACAGAAATCA

CD Infl.16.3 rev

GCAATCTTTTAGGTGACACTATAGAATACTCAAGCTATGCATCAAGCTTGGTACCGAGCT

CGGATCCACTAGTAACGGCCGCCAGTGTGCTGGAATTCGCCCTTCAGGCGTTGAATCCAA

TTAAACCGCAGGCTCCACGCGTTGTGGTGCTCCCCCGCCAATTCCTTTAAGTTTCAGTCT

TGCGACCGTACTTCCCAGGCGGCGGACTTAACAGCTTCCCTTCGGCACTGGGACAGCTCA

AAGCCACCCCAACACCAAGTCCGCATCGTTTACAGCTAGGACTACCCGGGTATCTAATCC

GGTTCGCGCCCCTAGCTTTCGTCCCTCACCGTCAGAATCGTTCAAGTCAGACGCCTTCGC

AACAGGCGGTCCTCCCAGGATTACAGAATTTCACCTCAACCCTGGGAGTACCTCTAACCT

CTCCCGATCTCAAGTCTAATAGTATCTCCAGCAATTCCCACAGTTAAGCTGCAGGATTTC

ACCAGAGACTTATTAAACCGGCTACGGCCGCTTTAGGCAAGGGCGAATTCTGCAGATATC

CATCACACTGGCGGCCGCTCGAGCATGCATCTAGAGGGCCCAATTCGCCCTATAGTGAGT

CGTATTACAATTCACTGGCCGTCGTTTTACAACGTCGTGACTGGGAAAACCCTGGCGTTA

CCCAACTTAATCGCCTTGCAGCACATCCCCCTTTCGCCAGCTGGCGTAATAGCGAAGAGG

CCCGCACCGATCGCCCTTCCCAACAGTTGCGCAGCCTGAATGGCGAATGGACGCGCCCTG

TAGCGGCGCATTAAGCGCGCGGGTGTGGTGGTTACGCGCAGCGTGACCGCTACACTTGCC

AGCGCCCTAGCGCCCCGCTCCTTTCGCTTTCTTCCCTTTCCTTTCTCCGCCACGTTTCCG

CCGGCTTTTCCCCGT

Crohn´s Disease Inflamed #2

CD Infl.17.1 for

TTTCGACTACTATAGGGCGAATTGGGCCCTCTAGATGCATGCTCGAGCGGCCGCCAGTGT

GATGGATATCTGCAGAATTCGCCCTTGCTTAAAGCGGCCGTAGCCGGTTTAATAAGTCTC

TGGTGAAATCCTGCAGCTTAACTGTGGGAATTGCTGGAGATACTATTAGACTTGAGATCG

GGAGAGGTTAGAGGTACTCCCAGGGTAGAGGTGAAATTCTGTAATCCTGGGAGGACCGCC

TGTTGCGAAGGCGTCTGACTGGAACGATTCTGACGGTGAGGGACGAAAGCTAGGGGCGCG

AACCGGATTAGATACCCGGGTAGTCCTAGCTGTAAACGATGCGGACTTGGTGTTGGGGTG

GCTTTGAGCTGTCCCAGTGCCGAAGGGAAGCTGTTAAGTCCGCCGCCTGGGAAGTACGGT

CGCAAGACTGAAACTTAAAGGAATTGGCGGGGGGGCACCACAACGCGTGGAGCCTGCGGT

TTAATTGGATTCAACGCCTGAAGGGCGAATTCCAGCACACTGGCGGCCGTTACTAGTGGA

TCCGAGCTCGGTACCAAGCTTGATGCATAGCTTGAGTATTCTATAGTGTCACCTAAATAG

CTTGGCGTAATCATGGTCATAGCTGTTTCCTGTGTGAAATTGTTATCCGCTCACAATTCC

ACACAACATACGAGCCGGAAGCATAAAGTGTAAAGCCTGGGGTGCCTAATGAGTGAGCTA

ACTCACATTAATTGCGTTGCGCTCACTGCCCGCTTTCCAGTCGGGAAACCTGTCGTGCCA

GCTGCATTAATGAATCGGCCAACGCGCGGGGAGAGGCGGTTTGCGTATTGGGCGCTCTTC

CGCTTCCTCGCTCACTGACTCGCTGCGCTCGGTCGTTCGGCTGCGGCGAGCGTTATCAGC

TCCACTCAAAGGCGGGTA

CD Infl.17.1 rev

TTCGCCAAGCTTTTAGGTGACACTATAGAATACTCAAGCTATGCATCAAGCTTGGTACCG

AGCTCGGATCCACTAGTAACGGCCGCCAGTGTGCTGGAATTCGCCCTTCAGGCGTTGAAT

CCAATTAAACCGCAGGCTCCACGCGTTGTGGTGCCCCCCCGCCAATTCCTTTAAGTTTCA

GTCTTGCGACCGTACTTCCCAGGCGGCGGACTTAACAGCTTCCCTTCGGCACTGGGACAG

CTCAAAGCCACCCCAACACCAAGTCCGCATCGTTTACAGCTAGGACTACCCGGGTATCTA

ATCCGGTTCGCGCCCCTAGCTTTCGTCCCTCACCGTCAGAATCGTTCCAGTCAGACGCCT

TCGCAACAGGCGGTCCTCCCAGGATTACAGAATTTCACCTCTACCCTGGGAGTACCTCTA

ACCTCTCCCGATCTCAAGTCTAATAGTATCTCCAGCAATTCCCACAGTTAAGCTGCAGGA

TTTCACCAGAGACTTATTAAACCGGCTACGGCCGCTTTAAGCAAGGGCGAATTCTGCAGA

TATCCATCACACTGGCGGCCGCTCGAGCATGCATCTAGAGGGCCCAATTCGCCCTATAGT

GAGTCGTATTACAATTCACTGGCCGTCGTTTTACAACGTCGTGACTGGGAAAACCCTGGC

GTTACCCAACTTAATCGCCTTGCAGCACATCCCCCTTTCGCCAGCTGGCGTAATAGCGAA

GAGGCCCGCACCGATCGCCCTTCCCAACAGTTGCGCAGCCTGAATGGCGAATGGACGCGC

CCTGTAGCGGCGCATTAAGCGCGGCGGGTGTGGTGGTTACGCGCAGCGTGACCGCTACAC

TTGCCAGCGCCCTAGCGCCCGCTCCTTTCGCTTTCTTCCCTTCCTTTTCTCGCCCACGTT

CGCCGGCTTTCCC

CD Infl.17.2 for

AACCGACCTACTATAGGGCGAATTGGGCCCTCTAGATGCATGCTCGAGCGGCCGCCAGTG

TGATGGATATCTGCAGAATTCGCCCTTGCTTAAAGCGGCCGTAGCCGGTTTAATAAGTCT

CTGGTGAAATCCTGCAGCTTAACTGTGGGAATTGCTGGAGATACTATTAGACTTGAGATC

GGGAGAGGTTAGAGGTACTCCCAGGGTAGAGGTGAAATTCTGTAATCCTGGGAGGACCGC

CTGTTGCGAAGGCGTCTGACTGGAACGATTCTGACGGTGAGGGACGAAAGCTAGGGGCGC

GAACCGGATTAGATACCCGGGTAGTCCTAGCTGTAAACGATGCGGACTTGGTGTTGGGGT

GGCTTTGAGCTGTCCCAGTGCCGAAGGGAAGCTGTTAAGTCCGCCACCTGGGAAGTACGG

TCGCAAGACTGAAACTTAAAGGAATTGGCGGGGGAGCACCACAACGCGTGGAGCCTGCGG

TTTAATTGGATTCAACGCCTGAAGGGCGAATTCCAGCACACTGGCGGCCGTTACTAGTGG

ATCCGAGCTCGGTACCAAGCTTGATGCATAGCTTGAGTATTCTATAGTGTCACCTAAATA

GCTTGGCGTAATCATGGTCATAGCTGTTTCCTGTGTGAAATTGTTATCCGCTCACAATTC

CACACAACATACGAGCCGGAAGCATAAAGTGTAAAGCCTGGGGTGCCTAATGAGTGAGCT

AACTCACATTAATTGCGTTGCGCTCACTGCCCGCTTTCCAGTCGGGAAACCTGTCGTGCC

AGCTGCATTAATGAATCGGCCAACGCGCGGGGAGAGGCGGTTTGCGTATTGGGCGCTCTT

CCGCTTCCTCGCTCACTGACTCGCTGCGCTCGGTCGTTCGGCTGCGGCGAGCGGTATCAG

CTCACTCAAAGGCGGTAAATAA

CD Infl.17.2 rev

TTCGGCCACCTTTTAGGTGACACTATAGAATACTCAAGCTATGCATCAAGCTTGGTACCG

AGCTCGGATCCACTAGTAACGGCCGCCAGTGTGCTGGAATTCGCCCTTCAGGCGTTGAAT

CCAATTAAACCGCAGGCTCCACGCGTTGTGGTGCTCCCCCGCCAATTCCTTTAAGTTTCA

GTCTTGCGACCGTACTTCCCAGGTGGCGGACTTAACAGCTTCCCTTCGGCACTGGGACAG

CTCAAAGCCACCCCAACACCAAGTCCGCATCGTTTACAGCTAGGACTACCCGGGTATCTA

ATCCGGTTCGCGCCCCTAGCTTTCGTCCCTCACCGTCAGAATCGTTCCAGTCAGACGCCT

TCGCAACAGGCGGTCCTCCCAGGATTACAGAATTTCACCTCTACCCTGGGAGTACCTCTA

ACCTCTCCCGATCTCAAGTCTAATAGTATCTCCAGCAATTCCCACAGTTAAGCTGCAGGA

TTTCACCAGAGACTTATTAAACCGGCTACGGCCGCTTTAAGCAAGGGCGAATTCTGCAGA

TATCCATCACACTGGCGGCCGCTCGAGCATGCATCTAGAGGGCCCAATTCGCCCTATAGT

GAGTCGTATTACAATTCACTGGCCGTCGTTTTACAACGTCGTGACTGGGAAAACCCTGGC

GTTACCCAACTTAATCGCCTTGCAGCACATCCCCCTTTCGCCAGCTGGCGTAATAGCGAA

GAGGCCCGCACCGATCGCCCTTCCCAACAGTTGCGCAGCCTGAATGGCGAATGGACGCGC

CCTGTAGCGGCGCATTAAGCGCGGCGGGTGTGGTGGTTACGCGCAGCGTGACCGCTACAC

TTGCCAGCGCCCTAGCGCCCGCTCCTTTCGCTTTCTTCCCTTCCTTTCTCGCCCACGTTC

GCCGGCTTTCCCCGTCAAGCTTCTAAATCGGGGGCCTCC

CD Infl.17.3 for

TACCGACTACTATAGGGCGAATTGGGCCCTCTAGATGCATGCTCGAGCGGCCGCCAGTGT

GATGGATATCTGCAGAATTCGCCCTTGCCTAAAGCAGCCGTAGCCGGTTTGATAAGTCTT

TGGTGAAAGCTTGTAGCTTAACTATAAGAATTGCTGAAGATACTGTCAGACTTGAAGTCG

GGAGAGGTTAGAGGTACTACCGGGGTAGGGGTGAAATCCTATAATCCTGGGAGGACCACC

TGTGGCGAAGGCGTCTAACTGGAACGATCTTGACGGTGAGTAACGAAAGCCAGGGGCGCG

AACCGGATTAGATACCCGGGTAGTCCTGGCCGTAAACGATGTGGACTTGGTGTTGGAATG

GCCTCGAGTTGTTCCGGTGCCGAAGGGAAGCTGTTAAGTCCACCGCCTGGGAAGTACGGT

CGCAAGACTGAAACTTAAAGGAATTGGCGGGGGAGCACCACAACGCGTGGAGCCTGCGGT

TCAATTGGATTCAACGCCTGAAGGGCGAATTCCAGCACACTGGCGGCCGTTACTAGTGGA

TCCGAGCTCGGTACCAAGCTTGATGCATAGCTTGAGTATTCTATAGTGTCACCTAAATAG

CTTGGCGTAATCATGGTCATAGCTGTTTCCTGTGTGAAATTGTTATCCGCTCACAATTCC

ACACAACATACGAGCCGGAAGCATAAAGTGTAAAGCCTGGGGTGCCTAATGAGTGAGCTA

ACTCACATTAATTGCGTTGCGCTCACTGCCCGCTTTCCAGTCGGGAAACCTGTCGTGCCA

GCTGCATTAATGAATCGGCCAACGCGCGGGGAGAGGCGGTTTGCGTATTGGGCGCTCTTC

CGCTTCCTCGCTCACTGACTCGCTGCGCTCGGTCGTTCGGCTGCGGCGAGCGGTATCAGC

TCACTCAAAGGCGGTAATACCGGTTATTCCCACAGAATTCAGGGGATT

CD Infl.17.3 rev

TCCGCCAACTTTTAGGTGACACTATAGAATACTCAAGCTATGCATCAAGCTTGGTACCGA

GCTCGGATCCACTAGTAACGGCCGCCAGTGTGCTGGAATTCGCCCTTCAGGCGTTGAATC

CAATTGAACCGCAGGCTCCACGCGTTGTGGTGCTCCCCCGCCAATTCCTTTAAGTTTCAG

TCTTGCGACCGTACTTCCCAGGCGGTGGACTTAACAGCTTCCCTTCGGCACCGGAACAAC

TCGAGGCCATTCCAACACCAAGTCCACATCGTTTACGGCCAGGACTACCCGGGTATCTAA

TCCGGTTCGCGCCCCTGGCTTTCGTTACTCACCGTCAAGATCGTTCCAGTTAGACGCCTT

CGCCACAGGTGGTCCTCCCAGGATTATAGGATTTCACCCCTACCCCGGTAGTACCTCTAA

CCTCTCCCGACTTCAAGTCTGACAGTATCTTCAGCAATTCTTATAGTTAAGCTACAAGCT

TTCACCAAAGACTTATCAAACCGGCTACGGCTGCTTTAGGCAAGGGCGAATTCTGCAGAT

ATCCATCACACTGGCGGCCGCTCGAGCATGCATCTAGAGGGCCCAATTCGCCCTATAGTG

AGTCGTATTACAATTCACTGGCCGTCGTTTTACAACGTCGTGACTGGGAAAACCCTGGCG

TTACCCAACTTAATCGCCTTGCAGCACATCCCCCTTTCGCCAGCTGGCGTAATAGCGAAG

AGGCCCGCACCGATCGCCCTTCCCAACAGTTGCGCAGCCTGAATGGCGAATGGACGCGCC

CTGTAGCGGCGCATTAAGCGCGGCGGGTGTGGTGGTTACGCGCAGCGTGACCGCTACACT

TGCCAGCGCCCTAGCGCCCGCTCCTTTCGCTTTCTTCCCTTCCTTTCTCGCCACGTTCGC

CGGCTTTCCCGTCAAGCTCTAAATCGGGGGCCTCCCTTTAGGTTCCG

Crohn´s Disease Inflamed #3

CD Infl.18.1 for

GAACCGACCTACTATAGGGCGAATTGGGCCCTCTAGATGCATGCTCGAGCGGCCGCCAGT

GTGATGGATATCTGCAGAATTCGCCCTTCAGGCGTTGAATCCAATTAAACCGCAGGCTCC

ACGCGTTGTGGTGCTCCCCCGCCAATTCCTTTAAGTTTCAGTCTTGCGACCGTACTTCCC

AGGCGGCGGACTTAACAGCTTCCCTTCGGCACTGGGACAGCTCAAAGCCACCCCAACACC

AAGTCCGCATCGTTTACAGCTAGGACTACCCGGGTATCTAATCCGGTTCGCGCCCCTAGC

TTTCGTCCCTCACCGTCAGAATCGTTCCAGTCAGACGCCTTCGCAACAGGCGGTCCTCCC

AGGATTACAGAATTTCACCTCTACCCTGGGAGTACCTCTAACCTCTCCCGATCTCAAGTC

TAATAGTATCTCCAGCAATTCCCACAGTTAAGCTGCAGGATTTCACCAGAGACTTATTAA

ACCGGCTACGGCCGCTTTAGGCAAGGGCGAATTCCAGCACACTGGCGGCCGTTACTAGTG

GATCCGAGCTCGGTACCAAGCTTGATGCATAGCTTGAGTATTCTATAGTGTCACCTAAAT

AGCTTGGCGTAATCATGGTCATAGCTGTTTCCTGTGTGAAATTGTTATCCGCTCACAATT

CCACACAACATACGAGCCGGAAGCATAAAGTGTAAAGCCTGGGGTGCCTAATGAGTGAGC

TAACTCACATTAATTGCGTTGCGCTCACTGCCCGCTTTCCAGTCGGGAAACCTGTCGTGC

CAGCTGCATTAATGAATCGGCCAACGCGCGGGGAGAGGCGGTTTGCGTATTGGGCGCTCT

TCCGCTTCTCGCTCACTGACTCGCTGCGCTCGGTCGTTCGGCTGCGGCGAGCGGTATCAG

CTCACTCAAAGGCGGTAATAACGGTTATTCCACAGGAT

CD Infl.18.1 rev

TTCGCCAACTTTTAGGTGACACTATAGAATACTCAAGCTATGCATCAAGCTTGGTACCGA

GCTCGGATCCACTAGTAACGGCCGCCAGTGTGCTGGAATTCGCCCTTGCCTAAAGCGGCC

GTAGCCGGTTTAATAAGTCTCTGGTGAAATCCTGCAGCTTAACTGTGGGAATTGCTGGAG

ATACTATTAGACTTGAGATCGGGAGAGGTTAGAGGTACTCCCAGGGTAGAGGTGAAATTC

TGTAATCCTGGGAGGACCGCCTGTTGCGAAGGCGTCTGACTGGAACGATTCTGACGGTGA

GGGACGAAAGCTAGGGGCGCGAACCGGATTAGATACCCGGGTAGTCCTAGCTGTAAACGA

TGCGGACTTGGTGTTGGGGTGGCTTTGAGCTGTCCCAGTGCCGAAGGGAAGCTGTTAAGT

CCGCCGCCTGGGAAGTACGGTCGCAAGACTGAAACTTAAAGGAATTGGCGGGGGAGCACC

ACAACGCGTGGAGCCTGCGGTTTAATTGGATTCAACGCCTGAAGGGCGAATTCTGCAGAT

ATCCATCACACTGGCGGCCGCTCGAGCATGCATCTAGAGGGCCCAATTCGCCCTATAGTG

AGTCGTATTACAATTCACTGGCCGTCGTTTTACAACGTCGTGACTGGGAAAACCCTGGCG

TTACCCAACTTAATCGCCTTGCAGCACATCCCCCTTTCGCCAGCTGGCGTAATAGCGAAG

AGGCCCGCACCGATCGCCCTTCCCAACAGTTGCGCAGCCTGAATGGCGAATGGACGCGCC

CTGTAGCGGCGCATTAAGCGCGGCGGGTGTGGTGGTTACGCGCAGCGTGACCGCTACACT

TGCCAGCGCCCTAGCGCCGCTCCTTTCGCTTTCTTCCCTTCCTTTCTCGCACGTTCGCCG

GCTTTCCCCGTCAAGCTCTAATTCGGGGGCCTCCCTTTAGGGTTCCGGATTTAGTGC

CD Infl.18.2 for

AGTATCGACTCTATAGGGCGAATTGGGCCCTCTAGATGCATGCTCGAGCGGCCGCCAGTG

TGATGGATATCTGCAGAATTCGCCCTTCAGGCGTTGAATCCAATTAAACCGCAGGCTCCA

CGCGTTGTGGTGCTCCCCCGCCAATTCCTTTAAGTTTCAGTCTTGCGACCGTACTTCCCA

GGCGGCGGACTTAACAGCTTCCCTTCGGCGCTGGGACAGCTCAAAGCCACCCCAACACCA

AGTCCGCATCGTTTACAGCTAGGACTACCCGGGTATCTAATCCGGTTCGCGCCCCTAGCT

TTCGTCCCTCACCGTCAGAATCGTTCCAGTCAGACGCCTTCGCAACAGGCGGTCCTCCCA

GGATTACAGAATTTCACCTCTACCCTGGGAGTACCTCTAACCTCTCCCGATCTCAAGTCT

AATAGTATCTCCAGCAATTCCCACAGTTAAGCTGCAGGATTTTACCAGAGACTTATTAAA

CCGGCTACGGCCCCTTTAAGCAAGGGCGAATTCCAGCACACTGGCGGCCGTTACTAGTGG

ATCCGAGCTCGGTACCAAGCTTGATGCATAGCTTGAGTATTCTATAGTGTCACCTAAATA

GCTTGGCGTAATCATGGTCATAGCTGTTTCCTGTGTGAAATTGTTATCCGCTCACAATTC

CACACAACATACGAGCCGGAAGCATAAAGTGTAAAGCCTGGGGTGCCTAATGAGTGAGCT

AACTCACATTAATTGCGTTGCGCTCACTGCCCGCTTTCCAGTCGGGAAACCTGTCGTGCC

AGCTGCATTAATGAATCGGCCAACGCGCGGGGAGAGGCGGTTTGCGTATTGGGCGCTCTT

CCGCTTCCTCGCTCACTGACTCGCTGCGCTCGGTCGTTCGGCTGCGGCGAGCGGTATCAG

CTCACTCAAAGGCGGTAATACGGTTATCCACAGAATCAGGGGATACGGCAGGAAGGACAT

CD Infl.18.2 rev

TACGGCCCCACTTTTAGGTGACACTATAGAATACTCAAGCTATGCATCAAGCTTGGTACC

GAGCTCGGATCCACTAGTAACGGCCGCCAGTGTGCTGGAATTCGCCCTTGCTTAAAGGGG

CCGTAGCCGGTTTAATAAGTCTCTGGTAAAATCCTGCAGCTTAACTGTGGGAATTGCTGG

AGATACTATTAGACTTGAGATCGGGAGAGGTTAGAGGTACTCCCAGGGTAGAGGTGAAAT

TCTGTAATCCTGGGAGGACCGCCTGTTGCGAAGGCGTCTGACTGGAACGATTCTGACGGT

GAGGGACGAAAGCTAGGGGCGCGAACCGGATTAGATACCCGGGTAGTCCTAGCTGTAAAC

GATGCGGACTTGGTGTTGGGGTGGCTTTGAGCTGTCCCAGCGCCGAAGGGAAGCTGTTAA

GTCCGCCGCCTGGGAAGTACGGTCGCAAGACTGAAACTTAAAGGAATTGGCGGGGGAGCA

CCACAACGCGTGGAGCCTGCGGTTTAATTGGATTCAACGCCTGAAGGGCGAATTCTGCAG

ATATCCATCACACTGGCGGCCGCTCGAGCATGCATCTAGAGGGCCCAATTCGCCCTATAG

TGAGTCGTATTACAATTCACTGGCCGTCGTTTTACAACGTCGTGACTGGGAAAACCCTGG

CGTTACCCAACTTAATCGCCTTGCAGCACATCCCCCTTTCGCCAGCTGGCGTAATAGCGA

AGAGGCCCGCACCGATCGCCCTTCCCAACAGTTGCGCAGCCTGAATGGCGAATGGACGCG

CCCTGTAGCGGCGCATTAAGCGCGCGGGTGTGTGGTTACGCGCAGCGTGACCGCTACACT

TGCCAGCGCCCTAGCGCCCGCTCCTTTCGCTTTCTTCCCTTCCTTTCTCGCACGTTCGCG

CTTTCCCCGTCAGCTCTAAATCGGGGCTCCCTTTAGGGTTCCGATTAGGTGGCTTTACGG

CACCTCGACCCC

CD Infl.18.3 for

AGAATCGACTACTATAGGGCGAATTGGGCCCTCTAGATGCATGCTCGAGCGGCCGCCAGT

GTGATGGATATCTGCAGAATTCGCCCTTCAGGCGTTGAATCCAATTAAACCGCAGGCTCC

ACGCGTTGTGGTGCTCCCCCGCCAATTCCTTTAAGTTTCAGTCTTGCGACCGTACTTCCC

AGGCGGCGGACTTAACAGCTTCCCTTCGGCACTGGGACAGCTCAAAGCCACCCCAACACC

AAGTCCGCATCGTTTACAGCTAGGACTACCCGGGTATCTAATCCGGTTCGCGCCCCTAGC

TTTCGTCCCTCACCGTCAGAATCGTTCCAGTCAGACGCCTTCGCAACAGGCGGTCCTCCC

AGGATTACAGAATTTCACCTCTACCCTGGGAGTACCTCTAACCTCTCCCGATCTCAAGTC

TAATAGTATCTCCAGCAATTCCCACAGTTAAGCTGCAGGATTTCACCAGAGACTTATTAA

ACCGGCTACGGCCGCTTTAAGCAAGGGCGAATTCCAGCACACTGGCGGCCGTTACTAGTG

GATCCGAGCTCGGTACCAAGCTTGATGCATAGCTTGAGTATTCTATAGTGTCACCTAAAT

AGCTTGGCGTAATCATGGTCATAGCTGTTTCCTGTGTGAAATTGTTATCCGCTCACAATT

CCACACAACATACGAGCCGGAAGCATAAAGTGTAAAGCCTGGGGTGCCTAATGAGTGAGC

TAACTCACATTAATTGCGTTGCGCTCACTGCCCGCTTTCCAGTCGGGAAACCTGTCGTGC

CAGCTGCATTAATGAATCGGCCACGCGCGGGGAGAGGCGGTTTGCGTATTGGGCGCTCTT

CCGCTTCCTCGCCTCACTGACTCGCTGCGCTCGGTCGTCGGCTGCGGCGAGCGGTATCAG

CTCACTCAAGGCGGTAATACGGTTATCACCAGAATCAGGGATAACCGCCAGGAAG

CD Infl.18.3 rev

TTTCCCCCCCTATTTAGGTGACACTATAGAATACTCAAGCTATGCATCAAGCTTGGTACC

GAGCTCGGATCCACTAGTAACGGCCGCCAGTGTGCTGGAATTCGCCCTTGCTTAAAGCGG

CCGTAGCCGGTTTAATAAGTCTCTGGTGAAATCCTGCAGCTTAACTGTGGGAATTGCTGG

AGATACTATTAGACTTGAGATCGGGAGAGGTTAGAGGTACTCCCAGGGTAGAGGTGAAAT

TCTGTAATCCTGGGAGGACCGCCTGTTGCGAAGGCGTCTGACTGGAACGATTCTGACGGT

GAGGGACGAAAGCTAGGGGCGCGAACCGGATTAGATACCCGGGTAGTCCTAGCTGTAAAC

GATGCGGACTTGGTGTTGGGGTGGCTTTGAGCTGTCCCAGTGCCGAAGGGAAGCTGTTAA

GTCCGCCGCCTGGGAAGTACGGTCGCAAGACTGAAACTTAAAGGAATTGGCGGGGGAGCA

CCACAACGCGTGGAGCCTGCGGTTTAATTGGATTCAACGCCTGAAGGGCGAATTCTGCAG

ATATCCATCACACTGGCGGCCGCTCGAGCATGCATCTAGAGGGCCCAATTCGCCCTATAG

TGAGTCGTATTACAATTCACTGGCCGTCGTTTTACAACGTCGTGACTGGGAAAACCCTGG

CGTTACCCAACTTAATCGCCTTGCAGCACATCCCCCTTTCGCCAGCTGGCGTAATAGCGA

AGAGGCCCGCACCGATCGCCCTTCCCAACAGTTGCGCAGCCTGAATGGCGAATGGACGCG

CCCTGTAGCGGCGCATTAAGCGCGGCGGGTGTGGTGGTTACGCGCAGCGTGACCGCTACC

ACTTGCCAGCGCCCTAGCGCCCGCTCCTTTCGCTTTCTTCCCTTCTTTCTCGCACGTTCG

CCGCTTTCCCCGTCAAGCTCTAATCGGGGGCTCCTTAGGGTTCCGATTAAGTGCCTTACG

G

CD Infl.18.4 for

AGTATCCGACCTACTATAGGGCGAATTGGGCCCTCTAGATGCATGCTCGAGCGGCCGCCA

GTGTGATGGATATCTGCAGAATTCGCCCTTGCCTAAAGCGGCCGTAGCCGGTTTAATAAG

TCTCTGGTGAAATCCTGCAGCTTAACTGTGGGAATTGCTGGAGATACTATTAGACTTGAG

ATCGGGAGAGGTTAGAGGTACTCCCAGGGTAGAGGTGAAATTCTGTAATCCTGGGAGGAC

CGCCTGTTGCGAAGGCGTCTGACTGGAACGATTCTGACGGTGAGGGACGAAAGCTAGGGG

CGCGAACCGGATTAGATACCCGGGTAGTCCTAGCTGTAAACGATGCGGACTTGGTGTTGG

GGTGGCTTTGAGCTGTCCCAGTGCCGAAGGGAAGCTGTTAAGTCCGCCGCCTGGGAAGTA

CGGTCGCAAGACTGAAACTTAAAGGAATTGGCGGGGGAGCACCACAACGCGTGGAGCCTG

CGGTTTAATTGGATTCAACGCCTGAAGGGCGAATTCCAGCACACTGGCGGCCGTTACTAG

TGGATCCGAGCTCGGTACCAAGCTTGATGCATAGCTTGAGTATTCTATAGTGTCACCTAA

ATAGCTTGGCGTAATCATGGTCATAGCTGTTTCCTGTGTGAAATTGTTATCCGCTCACAA

TTCCACACAACATACGAGCCGGAAGCATAAAGTGTAAAGCCTGGGGTGCCTAATGAGTGA

GCTAACTCACATTAATTGCGTTGCGCTCACTGCCCGCTTTCCAGTCGGGAAACCTGTCGT

GCCAGCTGCATTAATGAATCGGCCAACGCGCGGGGAGAGGCGGTTTGCGTATTGGCGCTC

TTCCGCTTCCTCGCTCACTGACTCGCTGCGCTCGGTCGTTCGGCTGCGGCGAGCGGTATC

AGCTCACTTCAAAAGGCGGTAATACGGGTTATCCAACAGAAT

CD Infl.18.4 rev

TTCGCAAACCTTTTAGGTGACACTATAGAATACTCAAGCTATGCATCAAGCTTGGTACCG

AGCTCGGATCCACTAGTAACGGCCGCCAGTGTGCTGGAATTCGCCCTTCAGGCGTTGAAT

CCAATTAAACCGCAGGCTCCACGCGTTGTGGTGCTCCCCCGCCAATTCCTTTAAGTTTCA

GTCTTGCGACCGTACTTCCCAGGCGGCGGACTTAACAGCTTCCCTTCGGCACTGGGACAG

CTCAAAGCCACCCCAACACCAAGTCCGCATCGTTTACAGCTAGGACTACCCGGGTATCTA

ATCCGGTTCGCGCCCCTAGCTTTCGTCCCTCACCGTCAGAATCGTTCCAGTCAGACGCCT

TCGCAACAGGCGGTCCTCCCAGGATTACAGAATTTCACCTCTACCCTGGGAGTACCTCTA

ACCTCTCCCGATCTCAAGTCTAATAGTATCTCCAGCAATTCCCACAGTTAAGCTGCAGGA

TTTCACCAGAGACTTATTAAACCGGCTACGGCCGCTTTAGGCAAGGGCGAATTCTGCAGA

TATCCATCACACTGGCGGCCGCTCGAGCATGCATCTAGAGGGCCCAATTCGCCCTATAGT

GAGTCGTATTACAATTCACTGGCCGTCGTTTTACAACGTCGTGACTGGGAAAACCCTGGC

GTTACCCAACTTAATCGCCTTGCAGCACATCCCCCTTTCGCCAGCTGGCGTAATAGCGAA

GAGGCCCGCACCGATCGCCCTTCCCAACAGTTGCGCAGCCTGAATGGCGAATGGACGCGC

CCTGTAGCGGCGCATTAAGCGCGCGGGTGTGGTGGTTACGCGCAGCGTGACCGCTACACT

TGCCAGCGCCCTAGCGCCCGCTCCTTTCGCTTTCTTCCCTTTCCTTTCTCGCCACGTTCG

CCGGCTTTTCCCCGTCAAGCTCCTAAATCGGGGGCT

Crohn´s Disease Inflamed #5

CD Infl.20.1 for

ATCCGACCTAACTATAGGGCGAATTGGGCCCTCTAGATGCATGCTCGAGCGGCCGCCAGT

GTGATGGATATCTGCAGAATTCGCCCTTGCCTAAAGCAGCCGTAGCCGGTTTGATAAGTC

TTTGGTGAAAGCTTGTAGCTTAACTATAAGAATTGCTGAAGATACTGTCAGACTTGAAGT

CGGGAGAGGTTAGAGGTACTACCGGGGTAGGGGTGAAATCCTATAATCCTGGGAGGACCA

CCTGTGGCGAAGGCGTCTAACTGGAACGATCTTGACGGTGAGTAACGAAAGCCAGGGGCG

CGAACCGGATTAGATACCCGGGTAGTCCTGGCCGTAAACGATGTGGACTTGGTGTTGGAA

TGGCCTCGAGTTGTTCCGGCGCCGAAGGGAAGCTGTTAAGTCCACCGCCTGGGAAGTACG

GTCGCAAGACTGAAACTTAAAGGAATTGGCGGGGGAGCACCACAACGCGTGGAGCCTGCG

GTTTAATTGGATTCAACGCCTGAAGGGCGAATTCCAGCACACTGGCGGCCGTTACTAGTG

GATCCGAGCTCGGTACCAAGCTTGATGCATAGCTTGAGTATTCTATAGTGTCACCTAAAT

AGCTTGGCGTAATCATGGTCATAGCTGTTTCCTGTGTGAAATTGTTATCCGCTCACAATT

CCACACAACATACGAGCCGGAAGCATAAAGTGTAAAGCCTGGGGTGCCTAATGAGTGAGC

TAACTCACATTAATTGCGTTGCGCTCACTGCCCGCTTTCCAGTCGGGAAACCTGTCGTGC

CAGCTGCATTAATGAATCGGCCAACGCGCGGGGAGAGGCGGTTTGCGTATTGGGCGCTCT

TCCGCTTCCTCGCTCACTGACTCGCTGCGCTCGGTCGTTCGGCTGCGGCGAGCGGTATCA

GCTCACTCAAAGGCGGTATACGGTTATCCACCAGAATTCAGGGGATAAACGCAGGAAGAA

CATTGTGA

CD Infl.20.1 rev

TACGCCCACCATTTAGGTGACACTATAGAATACTCAAGCTATGCATCAAGCTTGGTACCG

AGCTCGGATCCACTAGTAACGGCCGCCAGTGTGCTGGAATTCGCCCTTCAGGCGTTGAAT

CCAATTAAACCGCAGGCTCCACGCGTTGTGGTGCTCCCCCGCCAATTCCTTTAAGTTTCA

GTCTTGCGACCGTACTTCCCAGGCGGTGGACTTAACAGCTTCCCTTCGGCGCCGGAACAA

CTCGAGGCCATTCCAACACCAAGTCCACATCGTTTACGGCCAGGACTACCCGGGTATCTA

ATCCGGTTCGCGCCCCTGGCTTTCGTTACTCACCGTCAAGATCGTTCCAGTTAGACGCCT

TCGCCACAGGTGGTCCTCCCAGGATTATAGGATTTCACCCCTACCCCGGTAGTACCTCTA

ACCTCTCCCGACTTCAAGTCTGACAGTATCTTCAGCAATTCTTATAGTTAAGCTACAAGC

TTTCACCAAAGACTTATCAAACCGGCTACGGCTGCTTTAGGCAAGGGCGAATTCTGCAGA

TATCCATCACACTGGCGGCCGCTCGAGCATGCATCTAGAGGGCCCAATTCGCCCTATAGT

GAGTCGTATTACAATTCACTGGCCGTCGTTTTACAACGTCGTGACTGGGAAAACCCTGGC

GTTACCCAACTTAATCGCCTTGCAGCACATCCCCCTTTCGCCAGCTGGCGTAATAGCGAA

GAGGCCCGCACCGATCGCCCTTCCCAACAGTTGCGCAGCCTGAATGGCGAATGGACGCGC

CCTGTAGCGGCGCATTAAGCGCGGCGGGTGTGGTGGTTACGCGCAGCGTGACCGCTACAC

TTGCCAGCGCCCTAGCGCCCGCTCCTTTCGCTTTCTTCCCTTCCTTTCTCGCACGTCGCC

GCTTTCCCGTCAAGCTCTAATCGGGGCTCCCTTTAGGGTTCCGATTAGTGGCTTACGGCC

ACCCTCCGACCCCAAAT

CD Infl.20.2 for

AGTTCCGACTCTATAGGGCGAATTGGGCCCTCTAGATGCATGCTCGAGCGGCCGCCAGTG

TGATGGATATCTGCAGAATTCGCCCTTGCCTAAAGCAGCCGTAGCCGGTTTGATAAGTCT

TTGGTGAAAGCTTGTAGCTTAACTATAAGAATTGCTGAAGATACTGTCAGACTTGAAGTC

GGGAGAGGTTAGAGGTACTACCGGGGTAGGGGTGAAATCCTATAATCCTGGGAGGACCAC

CTGTGGCGAAGGCGTCTAACTGGAACGATCTTGACGGTGAGTAACGAAAGCCAGGGGCGC

GAACCGGATTAGATACCCGGGTAGTCCTGGCCGTAAACGATGTGGACTTGGTGTTGGAAT

GGCCTCGAGTTGTTCCGGTGCCGGAGGGAAGCTGTTAAGTCCACCGCCTGAGAAGTACGG

TCGCAAGACTGAAACTTAAAGGAATTGGCGGGGGAGCACCACAACGCGTGGAGCCTGCGG

TTTAATTGGATTCAACGCCTGAAGGGCGAATTCCAGCACACTGGCGGCCGTTACTAGTGG

ATCCGAGCTCGGTACCAAGCTTGATGCATAGCTTGAGTATTCTATAGTGTCACCTAAATA

GCTTGGCGTAATCATGGTCATAGCTGTTTCCTGTGTGAAATTGTTATCCGCTCACAATTC

CACACAACATACGAGCCGGAAGCATAAAGTGTAAAGCCTGGGGTGCCTAATGAGTGAGCT

AACTCACATTAATTGCGTTGCGCTCACTGCCCGCTTTCCAGTCGGGAAACCTGTCGTGCC

AGCTGCATTAATGAATCGGCCAACGCGCGGGGAGAGGCGGTTTGCGTATTGGGCGCTCTT

CCGCTTCCTCGCTCACTGACTCGCTGCCGCTCGGTCGTTCGCTGCGGCGAGCGGTATCAG

CTCCCTCAAAGGCGGTAATACGGTTATCCCACAGAATCAAGGGGATAACCGCCAGGAAAG

AAACCATGTGGAGCA

CD Infl.20.2 rev

CACGGCCCACTTTTAGGTGACACTATAGAATACTCAAGCTATGCATCAAGCTTGGTACCG

AGCTCGGATCCACTAGTAACGGCCGCCAGTGTGCTGGAATTCGCCCTTCAGGCGTTGAAT

CCAATTAAACCGCAGGCTCCACGCGTTGTGGTGCTCCCCCGCCAATTCCTTTAAGTTTCA

GTCTTGCGACCGTACTTCTCAGGCGGTGGACTTAACAGCTTCCCTCCGGCACCGGAACAA

CTCGAGGCCATTCCAACACCAAGTCCACATCGTTTACGGCCAGGACTACCCGGGTATCTA

ATCCGGTTCGCGCCCCTGGCTTTCGTTACTCACCGTCAAGATCGTTCCAGTTAGACGCCT

TCGCCACAGGTGGTCCTCCCAGGATTATAGGATTTCACCCCTACCCCGGTAGTACCTCTA

ACCTCTCCCGACTTCAAGTCTGACAGTATCTTCAGCAATTCTTATAGTTAAGCTACAAGC

TTTCACCAAAGACTTATCAAACCGGCTACGGCTGCTTTAGGCAAGGGCGAATTCTGCAGA

TATCCATCACACTGGCGGCCGCTCGAGCATGCATCTAGAGGGCCCAATTCGCCCTATAGT

GAGTCGTATTACAATTCACTGGCCGTCGTTTTACAACGTCGTGACTGGGAAAACCCTGGC

GTTACCCAACTTAATCGCCTTGCAGCACATCCCCCTTTCGCCAGCTGGCGTAATAGCGAA

GAGGCCCGCACCGATCGCCCTTCCCAACAGTTGCGCAGCCTGAATGGCGAATGGACGCGC

CCTGTAGCGGCGCATTAAGCGCGGCGGGTGTGGTGGTTACGCGCAGCGTGACCGCTACAC

TTGCCAGCGCCCTAGCGCCCGCTCCTTTCGCTTTCTTCCCTTCCTTTCTCGCCACGTTCG

CCGGCTTTCCCGTCAAGCTCTAATCGGGGGCTCCTTTAGGGTCCGATTAGTGCTTACGCA

CCTCGACCCAAAAACTG

CD Infl.20.3 for

TGTATCGACTACTATAGGGCGAATTGGGCCCTCTAGATGCATGCTCGAGCGGCCGCCAGT

GTGATGGATATCTGCAGAATTCGCCCTTCAGGCGTTGAATCCAATTAAACCGCAGGCTCC

ACGCGTTGTGGTGCTCCCCCGCCAATTCCTTTAAGTTTCAGTCTTGCGACCGTACTTCTC

AGGCGGTGGACTTAACAGCTTCCCTTCGGCACCGGAACAACTCGAGGCCATTCCAACACC

AAGTCCACATCGTTTACGGCCAGGACTACCCGGGTATCTAATCCGGTTCGCGCCCCTGGC

TTTCGTTACTCACCGTCAAGATCGTTCCAGTTAGACGCCTTCGCCACAGGTGGTCCTCCC

AGGATTATAGGATTTCACCCCTACCCCGGTAGTACCTCTAACCTCTCCCGACTTCAAGTC

TGACAGTATCTTCAGCAATTCTTATAGTTAAGCTACAAGCTTTCACCAAAGACTTATCAA

ACCGGCTACGGCCGCTTTAGGCAAGGGCGAATTCCAGCACACTGGCGGCCGTTACTAGTG

GATCCGAGCTCGGTACCAAGCTTGATGCATAGCTTGAGTATTCTATAGTGTCACCTAAAT

AGCTTGGCGTAATCATGGTCATAGCTGTTTCCTGTGTGAAATTGTTATCCGCTCACAATT

CCACACAACATACGAGCCGGAAGCATAAAGTGTAAAGCCTGGGGTGCCTAATGAGTGAGC

TAACTCACATTAATTGCGTTGCGCTCACTGCCCGCTTTCCAGTCGGGAAACCTGTCGTGC

CAGCTGCATTAATGAATCGGCCAACGCGCGGGGAGAGGCGGTTTGCGTATTGGGCGCTCT

TCCGCTTCCTCGCTCACTGACTCGCTGCGCTCGGTCGTTCGGCTGCGCGAGCGTATTCAG

CTCACTCAAAGGCGGTATACGGTATCCACAAGATCAGGGGATAACCGCAGGAAAGAAACA

TG

CD Infl.20.3 rev

TATGGCCACTTTTAGGTGACACTATAGAATACTCAAGCTATGCATCAAGCTTGGTACCGA

GCTCGGATCCACTAGTAACGGCCGCCAGTGTGCTGGAATTCGCCCTTGCCTAAAGCGGCC

GTAGCCGGTTTGATAAGTCTTTGGTGAAAGCTTGTAGCTTAACTATAAGAATTGCTGAAG

ATACTGTCAGACTTGAAGTCGGGAGAGGTTAGAGGTACTACCGGGGTAGGGGTGAAATCC

TATAATCCTGGGAGGACCACCTGTGGCGAAGGCGTCTAACTGGAACGATCTTGACGGTGA

GTAACGAAAGCCAGGGGCGCGAACCGGATTAGATACCCGGGTAGTCCTGGCCGTAAACGA

TGTGGACTTGGTGTTGGAATGGCCTCGAGTTGTTCCGGTGCCGAAGGGAAGCTGTTAAGT

CCACCGCCTGAGAAGTACGGTCGCAAGACTGAAACTTAAAGGAATTGGCGGGGGAGCACC

ACAACGCGTGGAGCCTGCGGTTTAATTGGATTCAACGCCTGAAGGGCGAATTCTGCAGAT

ATCCATCACACTGGCGGCCGCTCGAGCATGCATCTAGAGGGCCCAATTCGCCCTATAGTG

AGTCGTATTACAATTCACTGGCCGTCGTTTTACAACGTCGTGACTGGGAAAACCCTGGCG

TTACCCAACTTAATCGCCTTGCAGCACATCCCCCTTTCGCCAGCTGGCGTAATAGCGAAG

AGGCCCGCACCGATCGCCCTTCCCAACAGTTGCGCAGCCTGAATGGCGAATGGACGCGCC

CTGTAGCGGCGCATTAAGCGCGGCGGGTGTGGTGGTTACGCGCAGCGTGACCGCTACACT

TGCCAGCGCCCTAGCGCCCCGCTCCTTTCGCTTTCTTCCCTTCTTTCTCGCCACGTTCGC

CGCTTTCCCGTCAAGCTTCTAATCGGGGGCCTCCCTTTAGGGTTCCG

CD Infl.20.4 for

CGAACGGACCTCTATAGGGCGAATTGGGCCCTCTAGATGCATGCTCGAGCGGCCGCCAGT

GTGATGGATATCTGCAGAATTCGCCCTTGCTTAAAGCGGCCGTAGCCGGTTTGATAAGTC

TTTGGTGAAAGCTTGTAGCTTAACTATAAGAATTGCTGAAGATACTGTCAGACTTGAAGT

CGGGAGAGGTTAGAGGTACTACCGGGGTAGGGGTGAAATCCTATAATCCTGGGAGGACCA

CCTGTGGCGAAGGCGTCTAACTGGAACGATCTTGACGGTGAGTAACGAAAGCCCAGGGGG

CGCGAAACCGGGATTAGATACCCCGGGTAGTCCCTGGCCGTAAACGATGTGGACTTGGTG

TTGGAAATGGCCTCGAGTTGTTCGGTGCCCAAGGGAAACTGGTTAAGTCCCCCCGCCTGG

GGAAGTACGGGCGCAAGATGAAACTTTAAAGGAATTGGCGGGGGGAGCCCCCCAAACCCC

CGGGGACCCGGGGGGTTAATTTGGGTTTAAACCCCTGGAAGGGGCGAAATCCCACCCCAC

GGGGGGGGCCGTTTTTTAGGGGAACCCAACCCCGGGCCCCAACCTTGGGGGCCAAAATTT

GGGTTTTTATAAGGGGCCCCCAAAAAACTTGGGGGAAAAAAGGGGAAAAAGGTTTTTTCC

GGGGAAAAATTTTTTTTCCCCCAAAATTCCCCCAAAATTAAAACCCGCGGGAAAAAAAAT

TTAAAAACCCGGGGGGGCCCAAAAAAGAAAACAACACCAACACTATTTTGTGGGGCCCCC

CCGCCCCCTTCTCCCGGAAAAAAATTGCTGTCGCCTTTTTTTATATATTACCCCCCC

CD Infl.20.4 rev

TACGCCGGCATTTAGGTGACACTATAGAATACTCAAGCAGGACAAAGCTTGGTACGAGCT

CGGATCCACTAGTAACGGCCGCCAGTGTGCTGGAATTCGCCCTTCAGGCGTTGAATCCAA

TTAAACCGCAGGCTCCACGCGTTGTGGTGCTCCCCCGCCAATTCCTTTAAGTTTCAGTCT

TGCGACCGTACTTCCCAGGCGGTGGACTTAACAGCTTCCCTTCGGCACCGGAACAACTCG

AGGCCATTCCAACACCAAGTCCACATCGTTTACGGCCAGGACTACCCGGGTATCTAATCC

GGTTCGCGCCCCTGGCTTTCGTTACTCACCGTCAAGATCGTTCCAGTTAGACGCCTTCGC

CACAGGTGGTCCTCCCAGGATTATAGGATTTCACCCCTACCCCGGTAGTACCTCTAACCT

CTCCCGACTTCAAGTCTGACAGTATCTTCAGCAATTCTTATAGTTAAGCTACAAGCTTTC

ACCAAAGACTTATCAAACCGGCTACGGCCGCTTTAAGCAAGGGCGAATTCTGCAGATATC

CATCACACTGGCGGCCGCTCGAGCATGCATCTAGAGGGCCCAATTCGCCCTATAGTGAGT

CGTATTACAATTCACTGGCCGTCGTTTTACAACGTCGTGACTGGGAAAACCCTGGCGTTA

CCCAACTTAATCGCCTTGCAGCACATCCCCCTTTCGCCAGCTGGCGTAATAGCGAAGAGG

CCCGCACCGATCGCCCTTCCCAACAGTTGCGCAGCCCTGAATGGCGAATGGACGCGCCCT

GTAGCGGCGCATTAAGCGCGGCGGGTGTGGTGGTTACGCGCAGCGTGACCGCTACACTTT

GCAGCGCCCTAGCGCCCGCTCCTTTCGCTTTCTTCCCTTCCTTTCTCGCCCCCGTTCGCC

CGGCTTTCCCCCGTCAAGCTCTAAATCCGGGGGCTC

Crohn´s Disease Uninflamed #1

CD Uninfl.21.1 for

ATCGACCTACTATAGGGCGAATTGGGCCCTCTAGATGCATGCTCGAGCGGCCGCCAGTGT

GATGGATATCTGCAGAATTCGCCCTTCTGGCGTTGAATCCAATTAAACCGCAGGCTCCAC

GCGTTGTGGTGCTCCCCCGCCAATTCCTTTAAGTTTCAGTCTTGCGACCGTACTTCCCAG

GCGGTGGACTTAACAGCTTCCCTTCGGCACCGGAACAACTCGAGGCCATTCCAACACCAA

GTCCACATCGTTTACGGCCAGGACTACCCGGGTATCTAATCCGGTTCGCGCCCCTGGCTT

TCGTTACTCACCGTCAAGATCGTTCCAGTTAGACGCCTTCGCCACAGGTGGTCCTCCCAG

GATTATAGGATTTCACCCCTACCCCGGTAGTACCTCTAACCTCTCCCGACTTCAAGTCTG

ACAGTATCTTCAGCAATTCTTATAGTTAAGCTACAAGCTTTCACCAAAGACTTATCAAAC

CGGCTACGGCCGCTTTAGGCAAGGGCGAATTCCAGCACACTGGCGGCCGTTACTAGTGGA

TCCGAGCTCGGTACCAAGCTTGATGCATAGCTTGAGTATTCTATAGTGTCACCTAAATAG

CTTGGCGTAATCATGGTCATAGCTGTTTCCTGTGTGAAATTGTTATCCGCTCACAATTCC

ACACAACATACGAGCCGGAAGCATAAAGTGTAAAGCCTGGGGTGCCTAATGAGTGAGCTA

ACTCACATTAATTGCGTTGCGCTCACTGCCCGCTTTCCAGTCGGGAAACCTGTCGTGCCA

GCTGCATTAATGAATCGGCCAACGCGCGGGGAGAGGCGGTTTGCGTATTGGGCGCTCTTC

CGCTTCCTCGCTCACTGACTCGCTGCGCTCGGTCGTTCGGCTGCGGCGAGCGGTATCAGC

TCACTCCAAAGGCGGTAATACCGGTTATCCCACAGAATTCAGGA

CD Uninfl.21.1 rev

TACGCCGCTATTTAGGTGACACTATAGAATACTCAAGCTATGCATCAAGCTTGGTACCGA

GCTCGGATCCACTAGTAACGGCCGCCAGTGTGCTGGAATTCGCCCTTGCCTAAAGCGGCC

GTAGCCGGTTTGATAAGTCTTTGGTGAAAGCTTGTAGCTTAACTATAAGAATTGCTGAAG

ATACTGTCAGACTTGAAGTCGGGAGAGGTTAGAGGTACTACCGGGGTAGGGGTGAAATCC

TATAATCCTGGGAGGACCACCTGTGGCGAAGGCGTCTAACTGGAACGATCTTGACGGTGA

GTAACGAAAGCCAGGGGCGCGAACCGGATTAGATACCCGGGTAGTCCTGGCCGTAAACGA

TGTGGACTTGGTGTTGGAATGGCCTCGAGTTGTTCCGGTGCCGAAGGGAAGCTGTTAAGT

CCACCGCCTGGGAAGTACGGTCGCAAGACTGAAACTTAAAGGAATTGGCGGGGGAGCACC

ACAACGCGTGGAGCCTGCGGTTTAATTGGATTCAACGCCAGAAGGGCGAATTCTGCAGAT

ATCCATCACACTGGCGGCCGCTCGAGCATGCATCTAGAGGGCCCAATTCGCCCTATAGTG

AGTCGTATTACAATTCACTGGCCGTCGTTTTACAACGTCGTGACTGGGAAAACCCTGGCG

TTACCCAACTTAATCGCCTTGCAGCACATCCCCCTTTCGCCAGCTGGCGTAATAGCGAAG

AGGCCCGCACCGATCGCCCTTCCCAACAGTTGCGCAGCCTGAATGGCGAATGGACGCGCC

CTGTAGCGGCGCATTAAGCGCGGCGGGTGTGGTGGTTACGCGCAGCGTGACCGCTACACT

TGCCAGCGCCTAGCGCCCGCTCCTTTCGCTTTCTTCCCTTCC

CD Uninfl.21.2 for

TTTAAACGACTACTATAGGGCGAATTGGGCCCTCTAGATGCATGCTCGAGCGGCCGCCAG

TGTGATGGATATCTGCAGAATTCGCCCTTGCTTAAAGCGGCCGTAGCCGGTTTGATAAGT

CTTTGGTGAAAGCTTGTAGCTTAACTATAAGAATTGCTGAAGATACTGTCAGACTTGAAG

TCGGGAGAGGTTAGAGGTACTACCGGGGTAGGGGTGAAATCCTATAATCCTGGGAGGACC

ACCTGTGGCGGAGGCGTCTAACTGGAACGATCTTGGCGGTGAGTAACGAAAGCCAGGGGC

GCGAACCGGATTAGATACCCGGGTAGTCCTGGCCGTAAACGATGTGGACTTGGTGTTGGA

ATGGCCTCGAGTTGTTCCGGTGCCGAAGGGAAGCTGTTAAGTCCACCGCCTGGGAAGTAC

GGTCGCAAGACTGAAACTTAAAGGAATTGGCGGGGGAGCACCACAACGCGTGGAGCCTGC

GGTTTAATTGGATTCAACGCCTGAAGGGCGAATTCCAGCACACTGGCGGCCGTTACTAGT

GGATCCGAGCTCGGTACCAAGCTTGATGCATAGCTTGAGTATTCTATAGTGTCACCTAAA

TAGCTTGGCGTAATCATGGTCATAGCTGTTTCCTGTGTGAAATTGTTATCCGCTCACAAT

TCCACACAACATACGAGCCGGAAGCATAAAGTGTAAAGCCTGGGGTGCCTAATGAGTGAG

CTAACTCACATTAATTGCGTTGCGCTCACTGCCCGCTTTCCAGTCGGGAAAACCTGTCGT

GCCAGCTGCATTAATGAATCGGCCAACGCGCGGGGAGAGGCGGTTTGCGTATTGGGCGCT

CTTCCGCTTCCTCGCTCACTGACTCGCTGCGCTCGGTCGTTCGGC

CD Uninfl.21.2 rev

TTCGCCAACTTTTAGGTGACACTATAGAATACTCAAGCTATGCATCAAGCTTGGTACCGA

GCTCGGATCCACTAGTAACGGCCGCCAGTGTGCTGGAATTCGCCCTTCAGGCGTTGAATC

CAATTAAACCGCAGGCTCCACGCGTTGTGGTGCTCCCCCGCCAATTCCTTTAAGTTTCAG

TCTTGCGACCGTACTTCCCAGGCGGTGGACTTAACAGCTTCCCTTCGGCACCGGAACAAC

TCGAGGCCATTCCAACACCAAGTCCACATCGTTTACGGCCAGGACTACCCGGGTATCTAA

TCCGGTTCGCGCCCCTGGCTTTCGTTACTCACCGCCAAGATCGTTCCAGTTAGACGCCTC

CGCCACAGGTGGTCCTCCCAGGATTATAGGATTTCACCCCTACCCCGGTAGTACCTCTAA

CCTCTCCCGACTTCAAGTCTGACAGTATCTTCAGCAATTCTTATAGTTAAGCTACAAGCT

TTCACCAAAGACTTATCAAACCGGCTACGGCCGCTTTAAGCAAGGGCGAATTCTGCAGAT

ATCCATCACACTGGCGGCCGCTCGAGCATGCATCTAGAGGGCCCAATTCGCCCTATAGTG

AGTCGTATTACAATTCACTGGCCGTCGTTTTACAACGTCGTGACTGGGAAAACCCTGGCG

TTACCCAACTTAATCGCCTTGCAGCACATCCCCCTTTCGCCAGCTGGCGTAATAGCGAAG

AGGCCCGCACCGATCGCCCTTCCCAACAGTTGCGCAGCCTGAATGGCGAATGGACGCGCC

CTGTAGCGGCGCATTAAGCGCGGCGGGTGTGGTGGTTACGCGCAGCGTGACCGCTACACT

TGCCAGCGCCCTAGCGCCCGCTCCTTTCG

CD Uninfl.21.3 for

TAACGAACTACTATAGGGCGAATTGGGCCCTCTAGATGCATGCTCGAGCGGCCGCCAGTG

TGATGGATATCTGCAGAATTCGCCCTTCAGGCGTTGAATCCAATTAAACCGCAGGCTCCA

CGCGTTGTGGTGCTCCCCCGCCAATTCCTTTAAGTTTCAGTCTTGCGACCGTACTTCCCA

GGCGGTGGACTTAACAGCTTCCCTTCGGCACCGGAACAACTCGAGGCCATTCCAACACCA

AGTCCACATCGTTTACGGCCAGGACTACCCGGGTATCTAATCCGGTTCGCGCCCCTGGCT

TTCGTTACTCACCGTCAAGATCGTTCCAGTTAGACGCCTTCGCCACAGGTGGTCCTCCCA

GGATTATAGGATTTCACCCCTACCCCGGTAGTACCTCTAACCTCTCCCGACTTCAAGTCT

GACAGTATCTTCAGCAATTCTTATAGTTAAGCTACAAGCTTTCACCAAAGACTTATCAAA

CCGGCTACGGCCGCTTTAAGCAAGGGCGAATTCCAGCACACTGGCGGCCGTTACTAGTGG

ATCCGAGCTCGGTACCAAGCTTGATGCATAGCTTGAGTATTCTATAGTGTCACCTAAATA

GCTTGGCGTAATCATGGTCATAGCTGTTTCCTGTGTGAAATTGTTATCCGCTCACAATTC

CACACAACATACGAGCCGGAAGCATAAAGTGTAAAGCCTGGGGTGCCTAATGAGTGAGCT

AACTCACATTAATTGCGTTGCGCTCACTGCCCGCTTTCCAGTCGGGAAACCTGTCGTGCC

AGCTGCATTAATGAATCGGCCAACGCGCGGGGAGAGGCGGTTTGCGTATTGGGCGCTCTT

CCGCTTCCTCGCTCACCTGACTCGC

CD Uninfl.21.3 rev

CCCCCCCACCTTTTAGGTGACACTATAGAATACTCAAGCTATGCATCAAGCTTGGTACCG

AGCTCGGATCCACTAGTAACGGCCGCCAGTGTGCTGGAATTCGCCCTTGCTTAAAGCGGC

CGTAGCCGGTTTGATAAGTCTTTGGTGAAAGCTTGTAGCTTAACTATAAGAATTGCTGAA

GATACTGTCAGACTTGAAGTCGGGAGAGGTTAGAGGTACTACCGGGGTAGGGGTGAAATC

CTATAATCCTGGGAGGACCACCTGTGGCGAAGGCGTCTAACTGGAACGATCTTGACGGTG

AGTAACGAAAGCCAGGGGCGCGAACCGGATTAGATACCCGGGTAGTCCTGGCCGTAAACG

ATGTGGACTTGGTGTTGGAATGGCCTCGAGTTGTTCCGGTGCCGAAGGGAAGCTGTTAAG

TCCACCGCCTGGGAAGTACGGTCGCAAGACTGAAACTTAAAGGAATTGGCGGGGGAGCAC

CACAACGCGTGGAGCCTGCGGTTTAATTGGATTCAACGCCTGAAGGGCGAATTCTGCAGA

TATCCATCACACTGGCGGCCGCTCGAGCATGCATCTAGAGGGCCCAATTCGCCCTATAGT

GAGTCGTATTACAATTCACTGGCCGTCGTTTTACAACGTCGTGACTGGGAAAACCCTGGC

GTTACCCAACTTAATCGCCTTGCAGCACATCCCCCTTTCGCCAGCTGGCGTAATAGCGAA

GAGGCCCGCACCGATCGCCCTTCCCAACAGTTGCGCAGCCTGAATGGCGAATGGACGCGC

CCTGTAGCGGCGCATTAAGCGCGGCGGGTGTGGTGGTTACGCGCAGCGTGACCGCTACAC

TTGCCAGCGCCTAGCGCCCGCTCCTTTCGCTTTCTTCCCTTTCCTTTTCTTCCGCCACGT

TCCGCCGGCTTTTCCCCGGTCAAGCC

CD Uninfl.21.4 for

TGTTTTCGACTCTATAGGGCGAATTGGGCCCTCTAGATGCATGCTCGAGCGGCCGCCAGT

GTGATGGATATCTGCAGAATTCGCCCTTGCCTAAAGGGGCTGTAGCCGGTTTAATAAGTC

TCTGGTGAAATCCTGCAGCTTAACTGTGGGAATTGCTGGAGATACTATTAGACTTGAGAT

CGGGAGAGGTTAGAGGTACTCCCAGGGTAGAGGTGAAATTCTGTAATCCTGGGTGGACCG

CCTGTTGCGAAGGCGTCTGACTGGAACGATTCTGACGGTGAGGGACGAAAGCTAGGGGCG

CGAACCGGGTTAGATACCCGGGTAGTCCTAGCTGTAAACGATGCGGACTTGGTGTTGGGG

TGGCTTTGAGCTGTCCCAGTGCCGAAGGGAAGCTGTTAAGTCCGCCGCCTGGGAAGTACG

GTCGCAAGACTGAAACTTAAAGGAATTGGCGGGGGAGCACCACAACGCGTGGAGCCTGCG

GTTTAATTGGATTCAACGCCAGAAGGGCGAATTCCAGCACACTGGCGGCCGTTACTAGTG

GATCCGAGCTCGGTACCAAGCTTGATGCATAGCTTGAGTATTCTATAGTGTCACCTAAAT

AGCTTGGCGTAATCATGGTCATAGCTGTTTCCTGTGTGAAATTGTTATCCGCTCACAATT

CCACACAACATACGAGCCGGAAGCATAAAGTGTAAAGCCTGGGGGTGCCTAATGAGTGAG

CTAACTCACATTAATTGCGTTGCGCTCACTGCCCGCTTTCCAGTCGGGAAACCTGTCGTG

CCAGCTGCATTAATGAATCGGCCAACGCGCGGGGAGAGGCGGTTTGCGTATTGGGCGCTC

TTCCGCTTCCTCGCTCACTGACTCGCTGCGCTCGGTCGTTCGGCTGCGC

CD Uninfl.21.4 rev

TACGCCAACTTTTAGGTGACACTATAGAATACTCAAGCTATGCATCAAGCTTGGTACCGA

GCTCGGATCCACTAGTAACGGCCGCCAGTGTGCTGGAATTCGCCCTTCTGGCGTTGAATC

CAATTAAACCGCAGGCTCCACGCGTTGTGGTGCTCCCCCGCCAATTCCTTTAAGTTTCAG

TCTTGCGACCGTACTTCCCAGGCGGCGGACTTAACAGCTTCCCTTCGGCACTGGGACAGC

TCAAAGCCACCCCAACACCAAGTCCGCATCGTTTACAGCTAGGACTACCCGGGTATCTAA

CCCGGTTCGCGCCCCTAGCTTTCGTCCCTCACCGTCAGAATCGTTCCAGTCAGACGCCTT

CGCAACAGGCGGTCCACCCAGGATTACAGAATTTCACCTCTACCCTGGGAGTACCTCTAA

CCTCTCCCGATCTCAAGTCTAATAGTATCTCCAGCAATTCCCACAGTTAAGCTGCAGGAT

TTCACCAGAGACTTATTAAACCGGCTACAGCCCCTTTAGGCAAGGGCGAATTCTGCAGAT

ATCCATCACACTGGCGGCCGCTCGAGCATGCATCTAGAGGGCCCAATTCGCCCTATAGTG

AGTCGTATTACAATTCACTGGCCGTCGTTTTACAACGTCGTGACTGGGAAAACCCTGGCG

TTACCCAACTTAATCGCCTTGCAGCACATCCCCCTTTCGCCAGCTGGCGTAATAGCGAAG

AGGCCCGCACCGATCGCCCTTCCCAACAGTTGCGCAGCCTGAATGGCGAATGGACGCGCC

CTGTAGCGGCGCATTAAGCGCGGCGGGTGTGGTGGTTACGCGCAGCGTGACCGCTACACT

TGCCAGCGCCCTAGCGCCCGCTCCTTTCGCTTTTCTTTCCCTTTCCTTTTCTCGG

Crohn´s Disease Uninflamed #2

CD Uninfl.22.1 for

ATCCGACCTCTATAGGGCGAATTGGGCCCTCTAGATGCATGCTCGAGCGGCCGCCAGTGT

GATGGATATCTGCAGAATTCGCCCTTGCTTAAAGGGGCCGTAGCCGGTTTAATAAGTCTC

TGGTGAAATCCTGCAGCTTAACTGTGGGAATTGCTGGAGATACTATTAGACTTGAGATCG

GGAGAGGTTAGAGGTACTCCCAGGGTAGAGGTGAAATTCTGTAATCCTGGGAGGACCGCC

TGTTGCGAAGGCGTCTGACTGGAACGATTCTGACGGTGAGGGACGAAAGCTAGGGGCGCG

AACCGGATTAGATACCCGGGTAGTCCTAGCTGTAAACGATGCGGACTTGGTGTTGGGGTG

GCTTTGAGCTGTCCCAGTGCCGAAGGGAAGCTGTTAAGTCCGCCGCCTGGGAAGTACGGT

CGCAAGACTGAAACTTAAAGGAATTGGCGGGGGAGCACCACAACGCGTGGAGCCTGCGGT

TTAATTGGATTCAACGCCAGAAGGGCGAATTCCAGCACACTGGCGGCCGTTACTAGTGGA

TCCGAGCTCGGTACCAAGCTTGATGCATAGCTTGAGTATTCTATAGTGTCACCTAAATAG

CTTGGCGTAATCATGGTCATAGCTGTTTCCTGTGTGAAATTGTTATCCGCTCACAATTCC

ACACAACATACGAGCCGGAAGCATAAAGTGTAAAGCCTGGGGTGCCTAATGAGTGAGCTA

ACTCACATTAATTGCGTTGCGCTCACTGCCCGCTTTCCAGTCGGGAAACCTGTCGTGCCA

GCTGCATTAATGAATCGGCCAACGCGCGGGGAGAGGCGGTTTGCGTATTGGGCGCTCTTC

CGCTTCCTCGCTCACTGACTCGCTGCGCTCGGTCGTTCGCTGCGGCGAGGCGGTATCAGC

TCACCTCAAAGGCGTAATACCGGTTATCCACAGAATCAGGGGATAACGGCA

CD Uninfl.22.1 rev

TTCGCCCAACTTTTAGGTGACACTATAGAATACTCAAGCTATGCATCAAGCTTGGTACCG

AGCTCGGATCCACTAGTAACGGCCGCCAGTGTGCTGGAATTCGCCCTTCTGGCGTTGAAT

CCAATTAAACCGCAGGCTCCACGCGTTGTGGTGCTCCCCCGCCAATTCCTTTAAGTTTCA

GTCTTGCGACCGTACTTCCCAGGCGGCGGACTTAACAGCTTCCCTTCGGCACTGGGACAG

CTCAAAGCCACCCCAACACCAAGTCCGCATCGTTTACAGCTAGGACTACCCGGGTATCTA

ATCCGGTTCGCGCCCCTAGCTTTCGTCCCTCACCGTCAGAATCGTTCCAGTCAGACGCCT

TCGCAACAGGCGGTCCTCCCAGGATTACAGAATTTCACCTCTACCCTGGGAGTACCTCTA

ACCTCTCCCGATCTCAAGTCTAATAGTATCTCCAGCAATTCCCACAGTTAAGCTGCAGGA

TTTCACCAGAGACTTATTAAACCGGCTACGGCCCCTTTAAGCAAGGGCGAATTCTGCAGA

TATCCATCACACTGGCGGCCGCTCGAGCATGCATCTAGAGGGCCCAATTCGCCCTATAGT

GAGTCGTATTACAATTCACTGGCCGTCGTTTTACAACGTCGTGACTGGGAAAACCCTGGC

GTTACCCAACTTAATCGCCTTGCAGCACATCCCCCTTTCGCCAGCTGGCGTAATAGCGAA

GAGGCCCGCACCGATCGCCCTTCCCAACAGTTGCGCAGCCTGAATGGCGAATGGACGCGC

CCTGTAGCGGCGCATTAAGCGCGCGGGTGTGGTGGTTACGCGCAGCGTGACCGCTACAAC

TTGCCAGCGCCCTAGCGCCCGCTCCTTTCGCTTTCTTCCCTTCTTTCTCGCCACGTTCGC

CGCTTTCCCCGTCAAGCTCTAAATCGGGGCTCCCTTAGGTTCCGATTAGTGCTTTACGGC

ACCCTCGGAC

CD Uninfl.22.2 for

AGCATCGGACTCTATAGGGCGAATTGGGCCCTCTAGATGCATGCTCGAGCGGCCGCCAGT

GTGATGGATATCTGCAGAATTCGCCCTTGCCTAAAGCGGCCGTAGCCGGTTTAATAAGTC

TCTGGTGAAATCCTGCAGCTTAACTGTGGGAATTGCTGGAGATACTATTAGACTTGAGAT

CGGGAGAGGTTAGAGGTACTCCCAGGGTAGAGGTGAAATTCTGTAATCCTGGGAGGACCG

CCTGTTGCGAAGGCGTCTGACTGGAACGATTCTGACGGTGAGGGACGAAAGCTAGGGGCG

CGAACCGGATTAGATACCCGGGTAGTCCTAGCTGTAAACGATGCGGACTTGGTGTTGGGG

TGGCTTTGAGCTGTCCCAGTGCCGAAGGGAAGCTGTTAAGTCCGCCGCCTGGGAAGTACG

GTCGCAAGACTGAAACTTAAAGGAATTGGCGGGGGAGCACCACAACGCGTGGAGCCTGCG

GTTTAATTGGATTCAACGCCTGAAGGGCGAATTCCAGCACACTGGCGGCCGTTACTAGTG

GATCCGAGCTCGGTACCAAGCTTGATGCATAGCTTGAGTATTCTATAGTGTCACCTAAAT

AGCTTGGCGTAATCATGGTCATAGCTGTTTCCTGTGTGAAATTGTTATCCGCTCACAATT

CCACACAACATACGAGCCGGAAGCATAAAGTGTAAAGCCTGGGGTGCCTAATGAGTGAGC

TAACTCACATTAATTGCGTTGCGCTCACTGCCCGCTTTCCAGTCGGGAAACCTGTCGTGC

CAGCTGCATTAATGAATCGGCCAACGCGCGGGGAGAGGCGGTTTGCGTATTGGGCGCTCT

TCCGCTTCCTCGCTCACTGACTCGCTGCGCTCGGTCGTTCGGCTGCGGCGAGCGTATCAG

CTCACTCAAAGGCGGTAATACGGTTATCCACAGAATCAGGGATAACGGCAGGCA

CD Uninfl.22.2 rev

TAACGCCCACTATTTAGGTGACACTATAGAATACTCAAGCTATGCATCAAGCTTGGTACC

GAGCTCGGATCCACTAGTAACGGCCGCCAGTGTGCTGGAATTCGCCCTTCAGGCGTTGAA

TCCAATTAAACCGCAGGCTCCACGCGTTGTGGTGCTCCCCCGCCAATTCCTTTAAGTTTC

AGTCTTGCGACCGTACTTCCCAGGCGGCGGACTTAACAGCTTCCCTTCGGCACTGGGACA

GCTCAAAGCCACCCCAACACCAAGTCCGCATCGTTTACAGCTAGGACTACCCGGGTATCT

AATCCGGTTCGCGCCCCTAGCTTTCGTCCCTCACCGTCAGAATCGTTCCAGTCAGACGCC

TTCGCAACAGGCGGTCCTCCCAGGATTACAGAATTTCACCTCTACCCTGGGAGTACCTCT

AACCTCTCCCGATCTCAAGTCTAATAGTATCTCCAGCAATTCCCACAGTTAAGCTGCAGG

ATTTCACCAGAGACTTATTAAACCGGCTACGGCCGCTTTAGGCAAGGGCGAATTCTGCAG

ATATCCATCACACTGGCGGCCGCTCGAGCATGCATCTAGAGGGCCCAATTCGCCCTATAG

TGAGTCGTATTACAATTCACTGGCCGTCGTTTTACAACGTCGTGACTGGGAAACCCTGGC

GTTACCCAACTTAATCGCCTTGCAGCACATCCCCCTTTCGCCAGCTGGCGTAATAGCGAA

GAGGCCCGCACCGATCGCCCTTCCCAACAGTTGCGCAGCCTGAATGGCGAATGGACGCGC

CCTGTAGCGGCGCATTAAGCGCGGCGGGTGTGGTGGTTACGCGCAGCGTGACCGCTACAC

TTGCCAGCGCCTAGCGCCCGCTCCTTTCGCTTTCTTCCCTTCTTTCTCGCCACGTTCGCG

GCTTTCCCGTCAAGCTCTAAATCGGGGGCTCCTTTAGGGTTCCGATTTA

CD Uninfl.22.3 for

AACGCCCGCTTTTAGGTGACACTATAGAATACTCAAGCTATGCATCAAGCTTGGTACCGA

GCTCGGATCCACTAGTAACGGCCGCCAGTGTGCTGGAATTCGCCCTTGCCTAAAGCAGCC

GTAGCCGGTTTAATAAGTCTCTGGTGAAATCCTGCAGCTTAACTGTGGGAATTGCTGGAG

ATACTATTAGACTTGAGATCGGGAGAGGTTAGAGGTACTCCCAGGGTAGAGGTGAAATTC

TGTAATCCTGGGAGGACCGCCTGTTGCGAAGGCGTCTGACTGGAACGATTCTGACGGTGA

GGGACTAAAGCTAGGGGCGCGAACCGGATTAGATACCCGGGTAGCCCTAGCTGTAAACGA

TGCGGACTTGGTGTTGAGGTGGCTTTGAGCTGTCCCAGTGCCGAAGGGAAGCTGTTAAGT

CCGCCGCCTGGGAAGTACGGTCGCAAGACTGAAACTTAAAGGAATTGGCGGGGGAGCACC

ACAACGCGTGGAGCCTGCGGTTTAATTGGATTCAACGCCTGAAGGGCGAATTCTGCAGAT

ATCCATCACACTGGCGGCCGCTCGAGCATGCATCTAGAGGGCCCAATTCGCCCTATAGTG

AGTCGTATTACAATTCACTGGCCGTCGTTTTACAACGTCGTGACTGGGAAAACCCTGGCG

TTACCCAACTTAATCGCCTTGCAGCACATCCCCCTTTCGCCAGCTGGCGTAATAGCGAAG

AGGCCCGCACCGATCGCCCTTCCCAACAGTTGCGCAGCCTGAATGGCGAATGGACGCGCC

CTGTAGCGGCGCATTAAGCGCGGCGGGTGTGGTGGTTACGCGCAGCGTGACCGCTACACT

TGCCAGCGCCCTAGCGCCCGCTCCTTTCGCTTTCTTCCCTTCTTTTCTCGCCACGTTCGC

CGGCTTTTCCCCGTCAAGCTTCTAAATTCGG

CD Uninfl.22.3 rev

AGTTATACGACTCTATAGGGCGAATTGGGCCCTCTAGATGCATGCTCGAGCGGCCGCCAG

TGTGATGGATATCTGCAGAATTCGCCCTTCAGGCGTTGAATCCAATTAAACCGCAGGCTC

CACGCGTTGTGGTGCTCCCCCGCCAATTCCTTTAAGTTTCAGTCTTGCGACCGTACTTCC

CAGGCGGCGGACTTAACAGCTTCCCTTCGGCACTGGGACAGCTCAAAGCCACCTCAACAC

CAAGTCCGCATCGTTTACAGCTAGGGCTACCCGGGTATCTAATCCGGTTCGCGCCCCTAG

CTTTAGTCCCTCACCGTCAGAATCGTTCCAGTCAGACGCCTTCGCAACAGGCGGTCCTCC

CAGGATTACAGAATTTCACCTCTACCCTGGGAGTACCTCTAACCTCTCCCGATCTCAAGT

CTAATAGTATCTCCAGCAATTCCCACAGTTAAGCTGCAGGATTTCACCAGAGACTTATTA

AACCGGCTACGGCTGCTTTAGGCAAGGGCGAATTCCAGCACACTGGCGGCCGTTACTAGT

GGATCCGAGCTCGGTACCAAGCTTGATGCATAGCTTGAGTATTCTATAGTGTCACCTAAA

TAGCTTGGCGTAATCATGGTCATAGCTGTTTCCTGTGTGAAATTGTTATCCGCTCACAAT

TCCACACAACATACGAGCCGGAAGCATAAAGTGTAAAGCCTGGGGGTGCCTAATGAGTGA

GCTAACTCACATTAATTGCGTTGCGCTCACTGCCCGCTTTCCAGTCGGGAAACCTGTCGT

GCCAGCTGCATTAATGAATCGGCCAACGCGCGGGGAGAGGCGGTTTGCGTTATTGGGCGC

TCTTCCGCTTCCTCGCTCACTGACTCGCTGCGCTCGGTCGTTCGGCTGCGGCGAGCGGTA

TCAGCCTCACCTCAAAGGCGGTAAATACCGGTTATCACAAGAATCAA

Crohn´s Disease Uninflamed #3

CD Uninfl.23.1 for

TGTATACCGACTACTATAGGGCGAATTGGGCCCTCTAGATGCATGCTCGAGCGGCCGCCA

GTGTGATGGATATCTGCAGAATTCGCCCTTCTGGCGTTGAATCCAATTAAACCGCAGGCT

CCACGCGTTGTGGTGCTCCCCCGCCAATTCCTTTAAGTTTCAGTCTTGCGACCGTACTTC

CCAGGCGGTGGACTTAACAGCTTCCCTTCGGCACCGGAACAACTCGAGGCCATTCCAACA

CCAAGTCCACATCGTTTACGGCCAGGACTACCCGGGTATCTAATCCGGTTCGCGCCCCTG

GCTTTCGTTACTCACCGTCAAGATCGTTCCAGTTAGACGCCTTCGCCACAGGTGGTCCTC

CCAGGATTATAGGATTTCACCCCTACCCCGGTAGTACCTCTAACCTCTCCCGACTTCAAG

TCTGACAGTATCTTCAGCAATTCTTATAGTTAAGCTACAAGCTTTCACCAAAGACTTATC

AAACCGGCTACGGCCCCTTTAAGCAAGGGCGAATTCCAGCACACTGGCGGCCGTTACTAG

TGGATCCGAGCTCGGTACCAAGCTTGATGCATAGCTTGAGTATTCTATAGTGTCACCTAA

ATAGCTTGGCGTAATCATGGTCATAGCTGTTTCCTGTGTGAAATTGTTATCCGCTCACAA

TTCCACACAACATACGAGCCGGAAGCATAAAGTGTAAAGCCTGGGGTGCCTAATGAGTGA

GCTAACTCACATTAATTGCGTTGCGCTCACTGCCCGCTTTCCAGTCGGGAAACCTGTCGT

GCCAGCTGCATTAATGAATCGGCCAACGCGCGGGGAGAGGCGGTTTGCGTATTGGGCGCT

CTTCCGCTTCCTCGCTCACTGACTCGCTGCGCTCGGTCGTTCGGCTGCGGCGAGCGGTAT

CAGCTCACTCAAAAGC

CD Uninfl.23.1 rev

TACGCCCCGCTTTTAGGTGACACTATAGAATACTCAAGCTATGCATCAAGCTTGGTACCG

AGCTCGGATCCACTAGTAACGGCCGCCAGTGTGCTGGAATTCGCCCTTGCTTAAAGGGGC

CGTAGCCGGTTTGATAAGTCTTTGGTGAAAGCTTGTAGCTTAACTATAAGAATTGCTGAA

GATACTGTCAGACTTGAAGTCGGGAGAGGTTAGAGGTACTACCGGGGTAGGGGTGAAATC

CTATAATCCTGGGAGGACCACCTGTGGCGAAGGCGTCTAACTGGAACGATCTTGACGGTG

AGTAACGAAAGCCAGGGGCGCGAACCGGATTAGATACCCGGGTAGTCCTGGCCGTAAACG

ATGTGGACTTGGTGTTGGAATGGCCTCGAGTTGTTCCGGTGCCGAAGGGAAGCTGTTAAG

TCCACCGCCTGGGAAGTACGGTCGCAAGACTGAAACTTAAAGGAATTGGCGGGGGAGCAC

CACAACGCGTGGAGCCTGCGGTTTAATTGGATTCAACGCCAGAAGGGCGAATTCTGCAGA

TATCCATCACACTGGCGGCCGCTCGAGCATGCATCTAGAGGGCCCAATTCGCCCTATAGT

GAGTCGTATTACAATTCACTGGCCGTCGTTTTACAACGTCGTGACTGGGAAAACCCTGGC

GTTACCCAACTTAATCGCCTTGCAGCACATCCCCCTTTCGCCAGCTGGCGTAATAGCGAA

GAGGCCCGCACCGATCGCCCTTCCCAACAGTTGCGCAGCCTGAATGGCGAATGGACGCGC

CCTGTAGCGGCGCATTAAGCGCGGCGGGTGTGGTGGTTACGCGCAGCGTGACCGCTACAC

TTGCCAGCGCCCTAGCGCCCGCTCCTTTCGCTTTCTTCCCTTCCTTTTCTCGCCACGTTT

CGC

CD Uninfl.23.2 for

AGATAACCGACCTACTATAGGGCGAATTGGGCCCTCTAGATGCAAGCTCGAGCGGCCCCC

AGTGTGATGGATATCTGCAGAATTCGCCCTTGCTTAAAGGGGCCGTAGCCGGTTTGATAA

GTCTTTGGTGAAAGCTTGTAGCTTAACTATAAGAATTGCTGAAGATACTGCCAGACCTGA

AGTCGGGAGAGGTTAGAGGTACTACCGGGGTAGGGGTGAAATCCTATAATCCTGGGAGGA

CCACCTGTGGCGAAGGCGTCTAACTGGAACGATCTTGACGGTGATTAACGAAAGCCAGGG

GCGCGAACCGGATTAGATACCCGGGTAGTCCTGGCCGTAAACGATGTGGACTTGGTGTTG

GAATGGCCTCGAGTTGTTCCGGTGCCGAAGGGAAGCTGTTAAGTCCACCGCCTGGGAAGT

ACGGTCGCAAGACTGAAACTTAAAGGAATTGGCGGGGGAGCACCACAACGCGTGGAGCCT

GCGGTTTAATTGGATTCAACGCCAGAAGGGCGAATTCCAGCACACTGGCGGCCGTTACTA

GTGGATCCGAGCTCGGTACCAAGCTTGATGCATAGCTTGAGTATTCTATAGTGTCACCTA

AATAGCTTGGCGTAATCATGGTCATAGCTGTTTCCTGTGTGAAATTGTTATCCGCTCACA

ATTCCACACAACATACGAGCCGGAAGCATAAAGTGTAAAGCCTGGGGGTGCCTAATGAGT

GAGCTAACTCACATTAATTGCGTTGCGCTCACTGCCCGCTTTCCAGTCGGGAAACCTGTC

GTGCCAGCTGCATTAATGAATCGGCCCAACGCGCGGGGAGAGGCGGGTTTGCGTATTGGG

CGCTCTTCCGCTTCCTCGCTCACTGACTCGCTGCGCTCCGTCGTTCGGCTGCCGCGAAGC

GTATCAGCCTCAACTCAAAGGGCGGTAATACGGTTATCCACAGAAACTAAGGCGATATAC

GC

CD Uninfl.23.2 rev

GACCCCCAAGCTATTTAGGTGACACTATAGAATACTCAAGCTATGCATCAAGCTTGGTAC

CGAGCTCGGATCCACTAGTAACGGCCGCCAGTGTGCTGGAATTCGCCCTTCTGGCGTTGA

ATCCAATTAAACCGCAGGCTCCACGCGTTGTGGTGCTCCCCCGCCAATTCCTTTAAGTTT

CAGTCTTGCGACCGTACTTCCCAGGCGGTGGACTTAACAGCTTCCCTTCGGCACCGGAAC

AACTCGAGGCCATTCCAACACCAAGTCCACATCGTTTACGGCCAGGACTACCCGGGTATC

TAATCCGGTTCGCGCCCCTGGCTTTCGTTAATCACCGTCAAGATCGTTCCAGTTAGACGC

CTTCGCCACAGGTGGTCCTCCCAGGATTATAGGATTTCACCCCTACCCCGGTAGTACCTC

TAACCTCTCCCGACTTCAGGTCTGGCAGTATCTTCAGCAATTCTTATAGTTAAGCTACAA

GCTTTCACCAAAGACTTATCAAACCGGCTACGGCCCCTTTAAGCAAGGGCGAATTCTGCA

GATATCCATCACACTGGCGGCCGCTCGAGCATGCATCTAGAGGGCCCAATTCGCCCTATA

GTGAGTCGTATTACAATTCACTGGCCGTCGTTTTACAACGTCGTGACTGGGAAAACCCTG

GCGTTACCCAACTTAATCGCCTTGCAGCACATCCCCCTTTCGCCAGCTGGCGTAATAGCG

AAGAGGCCCGCACCGATCGCCCTTCCCAACAGTTGCGCAGCCTGAATGGCGAATGGACGC

GCCCTGTAGCGGCGCATTAAGCGCGCGGGTGTGGTGGTTACGCGCAGCGTGACCGCTACA

CTTGCCAGCGCCCTAGCGCCCGCTCCTTTCGCTTTTCTTTCCTTTCTTTTCTCGCAACGT

TCCGCCTGCTTTC

CD Uninfl.23.3 for

AATCGGACTACTATAGGGCGAATTGGGCCCTCTAGATGCATGCTCGAGCGGCCGCCAGTG

TGATGGATATCTGCAGAATTCGCCCTTCAGGCGTTGAATCCAATTAAACCGCAGGCTCCA

CGCGTTGTGGTGCTCCCCCGCCAATTCCTTTAAGTTTCAGTCTTGCGACCGTACTTCCCA

GGCGGTGGACTTAACAGCTTCCCTTCGGCACCGGAACAACTCGAGGCCATTCCAACACCA

AGTCCACATCGTTTACGGCCAGGACTACCCGGGTATCTAATCCGGTTCGCGCCCCTGGCT

TTCGTTACTCACCGTCAAGATCGTTCCAGTTAGACGCCTTCGCCACAGGTGGTCCTCCCT

GGATTATAGGATTTCACCCCTACCCCGGTAGTACCTCTAACCTCTCCCGACTTCAGGTCT

GACAGTATCTTCAGCAATTCTTATAGTTAAGCTACAAGCTTTCACCAAAGACTTATCAAA

CCGGCTACAGCCGCTTTAAGCAAGGGCGAATTCCAGCACACTGGCGGCCGTTACTAGTGG

ATCCGAGCTCGGTACCAAGCTTGATGCATAGCTTGAGTATTCTATAGTGTCACCTAAATA

GCTTGGCGTAATCATGGTCATAGCTGTTTCCTGTGTGAAATTGTTATCCGCTCACAATTC

CACACAACATACGAGCCGGAAGCATAAAGTGTAAAGCCTGGGGTGCCTAATGAGTGAGCT

AACTCACATTAATTGCGTTGCGCTCACTGCCCGCTTTCCAGTCGGGAAACCTGTCGTGCC

AGCTGCATTAATGAATCGGCCAACGCGCGGGGAGAGGCGGTTTGCGTATTGGGCGCTCTT

CCGCTTCCTCGCTCACTGACTCGCTGCGCTCGGTCGTTCGGCTGCGCGAGCGGTATCAGC

TCACTCAAAGGCGGTAATACG

CD Uninfl.23.3 rev

TACGGCCGCTTTTAGGTGACACTATAGAATACTCAAGCTATGCATCAAGCTTGGTACCGA

GCTCGGATCCACTAGTAACGGCCGCCAGTGTGCTGGAATTCGCCCTTGCTTAAAGCGGCT

GTAGCCGGTTTGATAAGTCTTTGGTGAAAGCTTGTAGCTTAACTATAAGAATTGCTGAAG

ATACTGTCAGACCTGAAGTCGGGAGAGGTTAGAGGTACTACCGGGGTAGGGGTGAAATCC

TATAATCCAGGGAGGACCACCTGTGGCGAAGGCGTCTAACTGGAACGATCTTGACGGTGA

GTAACGAAAGCCAGGGGCGCGAACCGGATTAGATACCCGGGTAGTCCTGGCCGTAAACGA

TGTGGACTTGGTGTTGGAATGGCCTCGAGTTGTTCCGGTGCCGAAGGGAAGCTGTTAAGT

CCACCGCCTGGGAAGTACGGTCGCAAGACTGAAACTTAAAGGAATTGGCGGGGGAGCACC

ACAACGCGTGGAGCCTGCGGTTTAATTGGATTCAACGCCTGAAGGGCGAATTCTGCAGAT

ATCCATCACACTGGCGGCCGCTCGAGCATGCATCTAGAGGGCCCAATTCGCCCTATAGTG

AGTCGTATTACAATTCACTGGCCGTCGTTTTACAACGTCGTGACTGGGAAAACCCTGGCG

TTACCCAACTTAATCGCCTTGCAGCACATCCCCCTTTCGCCAGCTGGCGTAATAGCGAAG

AGGCCCGCACCGATCGCCCTTCCCAACAGTTGCGCAGCCTGAATGGCGAATGGACGCGCC

CTGTAGCGGCGCATTAAGCGCGGCGGGTGTGGTGGTTACGCGCAGCGTGACCGCTACACT

TGCCAGCGCCCTAGCGCCCGCTCCTTTCGCTTTTCTTCCCTTCCTTTCCTCGCCACC

CD Uninfl.23.4 for

TGTAAACGACTACTATAGGGCGAATTGGGCCCTCTAGATGCATGCTCGAGCGGCCGCCAG

TGTGATGGATATCTGCAGAATTCGCCCTTCAGGCGTTGAATCCAATTAAACCGCAGGCTC

CACGCGTTGTGGTGCTCCCCCGCCAATTCCTTTAAGTTTCAGTCTTGCGACCGTACTTCC

CAGGCGGTGGACTTAACAGCTTCCCTTCGGCACCGGAACAACTCGAGGCCATTCCAACAC

CAAGTCCACATCGTTTACGGCCAGGACTACCCGGGTATCTAATCCGGTTCGCGCCCCTGG

CTTTCGTTACTCACCGTCAAGATCGTTCCAGTTAGACGCCTTCGCCACAGGTGGTCCTCC

CAGGATTATAGGATCTCACCCCTACCCCGGTAGTACCTCTAACCTCTCCCGACTTCAAGT

CTGACAGTATCTTCAGCAATTCTTATAGTTAAGCTACAAGCTTTCACCAAAGACTTATCA

AACCGGCTACGGCCGCTTTAGGCAAGGGCGAATTCCAGCACACTGGCGGCCGTTACTAGT

GGATCCGAGCTCGGTACCAAGCTTGATGCATAGCTTGAGTATTCTATAGTGTCACCTAAA

TAGCTTGGCGTAATCATGGTCATAGCTGTTTCCTGTGTGAAATTGTTATCCGCTCACAAT

TCCACACAACATACGAGCCGGAAGCATAAAGTGTAAAGCCTGGGGGTGCCTAATGAGTGA

GCTAACTCACATTAATTGCGTTGCGCTCACTGCCCGCTTTCCAGTCGGGAAACCTGTCGT

GCCAGCTGCATTAATGAATCGGCCAACGCGCGGGGAGAGGCGGTTTGCGTATTGGGCGCT

CTTCCGCTTCCTCGCTCACTGACTCGCTGCGCTCGGTCGTTCGGCTGCGGC

CD Uninfl.23.4 rev

AAGCCTTTTTTTAGGTGACACTATAGAATACTCAAGCTATGCATCAAGCTTGGTACCGAG

CTCGGATCCACTAGTAACGGCCGCCAGTGTGCTGGAATTCGCCCTTGCCTAAAGCGGCCG

TAGCCGGTTTGATAAGTCTTTGGTGAAAGCTTGTAGCTTAACTATAAGAATTGCTGAAGA

TACTGTCAGACTTGAAGTCGGGAGAGGTTAGAGGTACTACCGGGGTAGGGGTGAGATCCT

ATAATCCTGGGAGGACCACCTGTGGCGAAGGCGTCTAACTGGAACGATCTTGACGGTGAG

TAACGAAAGCCAGGGGCGCGAACCGGATTAGATACCCGGGTAGTCCTGGCCGTAAACGAT

GTGGACTTGGTGTTGGAATGGCCTCGAGTTGTTCCGGTGCCGAAGGGAAGCTGTTAAGTC

CACCGCCTGGGAAGTACGGTCGCAAGACTGAAACTTAAAGGAATTGGCGGGGGAGCACCA

CAACGCGTGGAGCCTGCGGTTTAATTGGATTCAACGCCTGAAGGGCGAATTCTGCAGATA

TCCATCACACTGGCGGCCGCTCGAGCATGCATCTAGAGGGCCCAATTCGCCCTATAGTGA

GTCGTATTACAATTCACTGGCCGTCGTTTTACAACGTCGTGACTGGGAAAACCCTGGCGT

TACCCAACTTAATCGCCTTGCAGCACATCCCCCTTTCGCCAGCTGGCGTAATAGCGAAGA

GGCCCGCACCGATCGCCCTTCCCAACAGTTGCGCAGCCTGAATGGCGAATGGACGCGCCC

TGTAGCGGCGCATTAAGCGCGCGGGTGTGGTGGTTACGCGCAGCGTGACCGCTACACTTG

CCAGCGCCCTAGCGCCCCGCTCCTTCCGCTTTCTTCCCTTCCTTTCTCCCACCGTTC

Crohn´s Disease Uninflamed #4

CD Uninfl.24.1 for

AGGTATACGACTACTATAGGGCGAATTGGGCCCTCTAGATGCATGCTCGAGCGGCCGCCA

GTGTGATGGATATCTGCAGAATTCGCCCTTCTGGCGTTGAATCCAATTAAACCGCAGGCT

CCACGCGTTGTGGTGCTCCCCCGCCAATTCCTTTAAGTTTCAGTCTTGCGACCGTACTTC

CCAGGCGGCGGACTAACAGCTTCCCTTCGGCACTGGGACAGCTCAAAGCCACCCCAACAC

CAAGTCCGCATCGTTTACAGCTAGGACTACCCGGGTATCTAATCCGGTTCGCACCCCTAG

CTTTCGTCCCTCACCGTCAGAATCGTTCCAGTCAGACGCCTTCGCAACAGGCGGTCCTCC

CAGGATTACAGAACTTCACCTCTACCCTGGGAGTACCTCTAACCTCTCCCGATCTCAAGT

CCAATAGTATCTCCAGCAATTCCCACAGTTAAGCTGCAGGATTTCACCAGAGACTTATTA

AACCGGCTACAGCCCCTTTAAGCAAGGGCGAATTCCAGCACACTGGCGGCCGTTACTAGT

GGATCCGAGCTCGGTACCAAGCTTGATGCATAGCTTGAGTATTCTATAGTGTCACCTAAA

TAGCTTGGCGTAATCATGGTCATAGCTGTTTCCTGTGTGAAATTGTTATCCGCTCACAAT

TCCACACAACATACGAGCCGGAAGCATAAAGTGTAAAGCCTGGGGTGCCTAATGAGTGAG

CTAACTCACATTAATTGCGTTGCGCTCACTGCCCGCTTTCCAGTCGGGAAACCTGTCGTG

CCAGCTGCATTAATGAATCGGCCAACGCGCGGGGAGAGGCGGTTTGCGTATTGGGCGCTC

TTCCGCTTCCTCGCTCACTGACTCGCTGCCGCTCGGTCG

CD Uninfl.24.1 rev

CCCGCCCCACTTTTAGGTGACACTATAGAATACTCAAGCTATGCATCAAGCTTGGTACCG

AGCTCGGATCCACTAGTAACGGCCGCCAGTGTGCTGGAATTCGCCCTTGCTTAAAGGGGC

TGTAGCCGGTTTAATAAGTCTCTGGTGAAATCCTGCAGCTTAACTGTGGGAATTGCTGGA

GATACTATTGGACTTGAGATCGGGAGAGGTTAGAGGTACTCCCAGGGTAGAGGTGAAGTT

CTGTAATCCTGGGAGGACCGCCTGTTGCGAAGGCGTCTGACTGGAACGATTCTGACGGTG

AGGGACGAAAGCTAGGGGTGCGAACCGGATTAGATACCCGGGTAGTCCTAGCTGTAAACG

ATGCGGACTTGGTGTTGGGGTGGCTTTGAGCTGTCCCAGTGCCGAAGGGAAGCTGTTAGT

CCGCCGCCTGGGAAGTACGGTCGCAAGACTGAAACTTAAAGGAATTGGCGGGGGAGCACC

ACAACGCGTGGAGCCTGCGGTTTAATTGGATTCAACGCCAGAAGGGCGAATTCTGCAGAT

ATCCATCACACTGGCGGCCGCTCGAGCATGCATCTAGAGGGCCCAATTCGCCCTATAGTG

AGTCGTATTACAATTCACTGGCCGTCGTTTTACAACGTCGTGACTGGGAAAACCCTGGCG

TTACCCAACTTAATCGCCTTGCAGCACATCCCCCTTTCGCCAGCTGGCGTAATAGCGAAG

AGGCCCGCACCGATCGCCCTTCCCAACAGTTGCGCAGCCTGAATGGCGAATGGACGCGCC

CTGTAGCGGCGCATTAAGCGCGGCGGGTGTGGTGGTTACGCGCAGCGTGACCGCTACACT

TGCCAGCGCCCTAGCGCCCGCTCCTTTTCGCTTTTCTTCCC

CD Uninfl.24.2 for

GTGTTATCCCACCTACTATAGGGCGAATTGGGCCCTCTAGATGCATGCTCGAGCGGCCGC

CAGTGTGATGGATATCTGCAGAATTCGCCCTTGCCTAAAGCGGCCGTAGCCGGTTTAATA

AGTCTCTGGTGAAATCCTGCAGCTTAACTGTGGGAATTGCTGGAGATACTATTAGACTTG

AGATCGGGAGAGGTTAGAGGTACTCC

CD Uninfl.24.2 rev

GACGCCCACCATTTAGGTGACACTATAGAATACTCAAGCTATGCATCAAGCTTGGTACCG

AGCTCGGATCCACTAGTAACGGCCGCCAGTGTGCTGGAATTCGCCCTTCTGGCGTTGAAT

CCAATTAAACCGCAGGCTCCACGCGTTGTGGTGCTCCCCCGCCAATTCCTTTAAGTTTCA

GTCTTGCAACCGTACTTCCCAGGCGGCGGACTTAACAGCTTCCCTTCGGCACTGGGACAG

CTCAAAGCCACCCCAACACCAAGTCCGCATCATTTACAGCTAGGACTACCCGGGTATCTA

ATCCGGTCCGCGCCCCTAGCTTTCGTCCCTCACCGTCAGAATCGTTCCAGTCAGACGCCT

TCGCAACAGGCGGTCCTCCCAGGATTACAGAATTTCACCTCTACCCTGGGAGTACCTCTA

ACCTCTCCCGATCTCAAGTCTAATAGTATCTCCAGCAATTCCCACAGTTAAGCTGCAGGA

TTTCACCAGAGACTTATTAAACCGGCTACGGCCGCTTTAGGCAAGGGCGAATTCTGCAGA

TATCCATCACACTGGCGGCCGCTCGAGCATGCATCTAGAGGGCCCAATTCGCCCTATAGT

GAGTCGTATTACAATTCACTGGCCGTCGTTTTACAACGTCGTGACTGGGAAAACCCTGGC

GTTACCCAACTTAATCGCCTTGCAGCACATCCCCCTTTCGCCAGCTGGCGTAATAGCGAA

GAGGCCCGCACCGATCGCCCTTCCCAACAGTTGCGCAGCCTGAATGGCGAATGGACGCGC

CCTGTAGCGGCGCATTAAGCGCGGCGGGTGTGGTGGTTACGCGCAGCGTGACCGCTACAC

TTGCCCAGCGCCCTAGCGCCCGCTCCTTTCGCTTTTCTTCCCTTCCTT

CD Uninfl.24.3 for

AGTATTCGACTTCTATAGGGCGAATTGGGCCCTCTAGATGCATGCTCGAGCGGCCGCCAG

TGTGATGGATATCTGCAGAATTCGCCCTTGCCTAAAGCGGCCGTAGCCTTTTGCACTACC

TCGTCACTAGCAGAAAATAGGATTTTTCACCTGCAAATGGTGAAGAGAGAAGAGGCTAGC

CTAAACATTAGGTAGAAATTACTTGTCAAGTTTGATCAATTCACAGAAAATCCAAATCCC

CTTTGAAAAGGCACAGTTGATACTCAGAGATTAGCGGGGATGTCCTCACAGATCCATTGT

TTGGCTAAATTGTCTGCATTTGCAGCATGTTTGCTAAATGAGTGATACATTTGCATCTAA

ATGGTCCATAAATAATTGGATTCAACGCCTGAAGGGCGAATTCCAGCACACTGGCGGCCG

TTACTAGTGGATCCGAGCTCGGTACCAAGCTTGATGCATAGCTTGAGTATTCTATAGTGT

CACCTAAATAGCTTGGCGTAATCATGGTCATAGCTGTTTCCTGTGTGAAATTGTTATCCG

CTCACAATTCCACACAACATACGAGCCGGAAGCATAAAGTGTAAAGCCTGGGGTGCCTAA

TGAGTGAGCTAACTCACATTAATTGCGTTGCGCTCACTGCCCGCTTTCCAGTCGGGAAAC

CTGTCGTGCCAGCTGCATTAATGAATCGGCCAACGCGCGGGGAGAGGCGGTTTGCGTATT

GGGCGCTCTTCCGCTTCCTCGCTCACTGACTCGCTGCGCTCGGTCGTTCGGCTGCGGCGA

GCGGTATCAGCTCACTCAAAGGCGGTAATACGGTTATCCACAGAATCAGGGGATAACGCA

GGAAAGAACATGTGAGCAAAAGGCCAG

CD Uninfl.24.3 rev

CCCCCCAAACCCATTTAGGTGACACTATAGAATACTCAAGCTATGCATCAAGCTTGGTAC

CGAGCTCGGATCCACTAGTAACGGCCGCCAGTGTGCTGGAATTCGCCCTTCAGGCGTTGA

ATCCAATTATTTATGGACCATTTAGATGCAAATGTATCACTCATTTAGCAAACATGCTGC

AAATGCAGACAATTTAGCCAAACAATGGATCTGTGAGGACATCCCCGCTAATCTCTGAGT

ATCAACTGTGCCTTTTCAAAGGGGATTTGGATTTTCTGTGAATTGATCAAACTTGACAAG

TAATTTCTACCTAATGTTTAGGCTAGCCTCTTCTCTCTTCACCATTTGCAGGTGAAAAAT

CCTATTTTCTGCTAGTGACGAGGTAGTGCAAAAGGCTACGGCCGCTTTAGGCAAGGGCGA

ATTCTGCAGATATCCATCACACTGGCGGCCGCTCGAGCATGCATCTAGAGGGCCCAATTC

GCCCTATAGTGAGTCGTATTACAATTCACTGGCCGTCGTTTTACAACGTCGTGACTGGGA

AAACCCTGGCGTTACCCAACTTAATCGCCTTGCAGCACATCCCCCTTTCGCCAGCTGGCG

TAATAGCGAAGAGGCCCGCACCGATCGCCCTTCCCAACAGTTGCGCAGCCTGAATGGCGA

ATGGACGCGCCCTGTAGCGGCGCATTAAGCGCGGCGGGTGTGGTGGTTACGCGCAGCGTG

ACCGCTACACTTGCCAGCGCCCTAGCGCCCGCTCCTTTCGCTTTCTTCCCTTCCTTTCTC

GCCACGTTCGCCGGCTTTCCCCGTCAAGCTCTAAATCGGGGGCTCCCTTTAGGGTTCCGA

TTTAGTGCTTTACGGCACCTCGACCCCAAAAAACTTGATTAGGGTGATG
