# Supplementary figures and images for: First Insights into the Diverse Human Archaeome: Specific Detection of Archaea in the Gastrointestinal Tract, Lung, and Nose and on Skin
Source: mBio. 2017 Nov 14;8(6):e00824-17. doi: 10.1128/mBio.00824-17 (PMC5686531; doi:10.1128/mBio.00824-17)

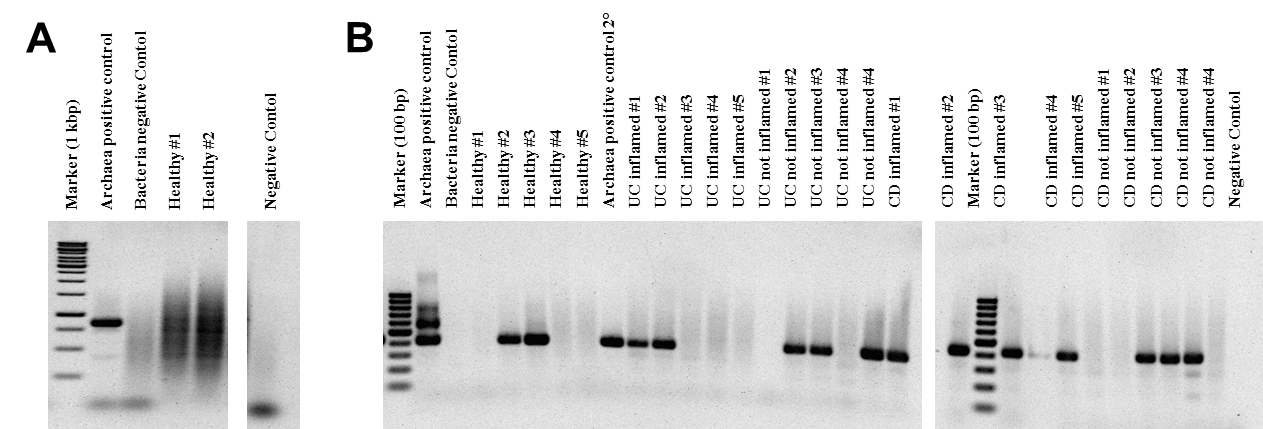

Supplement: FIG S1 [file mbo005173581sf1.tif]

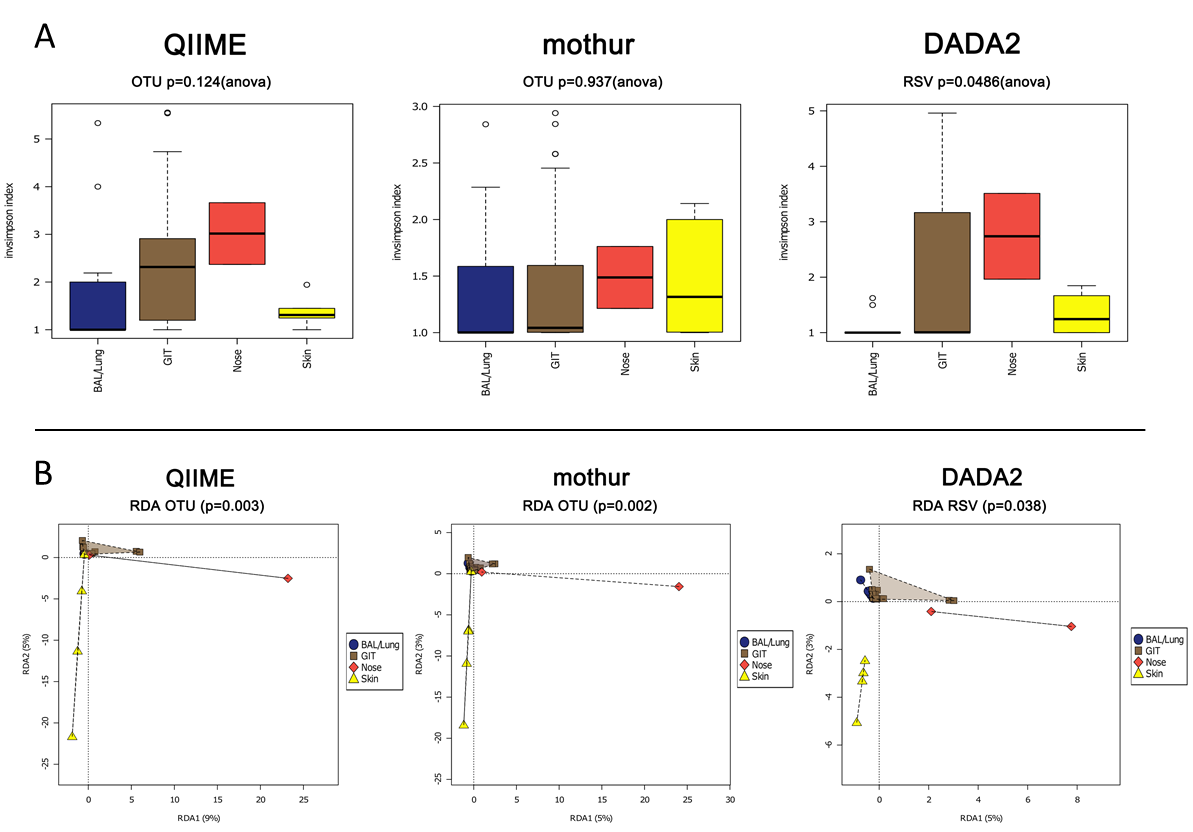

Supplement: FIG S2 [file mbo005173581sf2.tif]

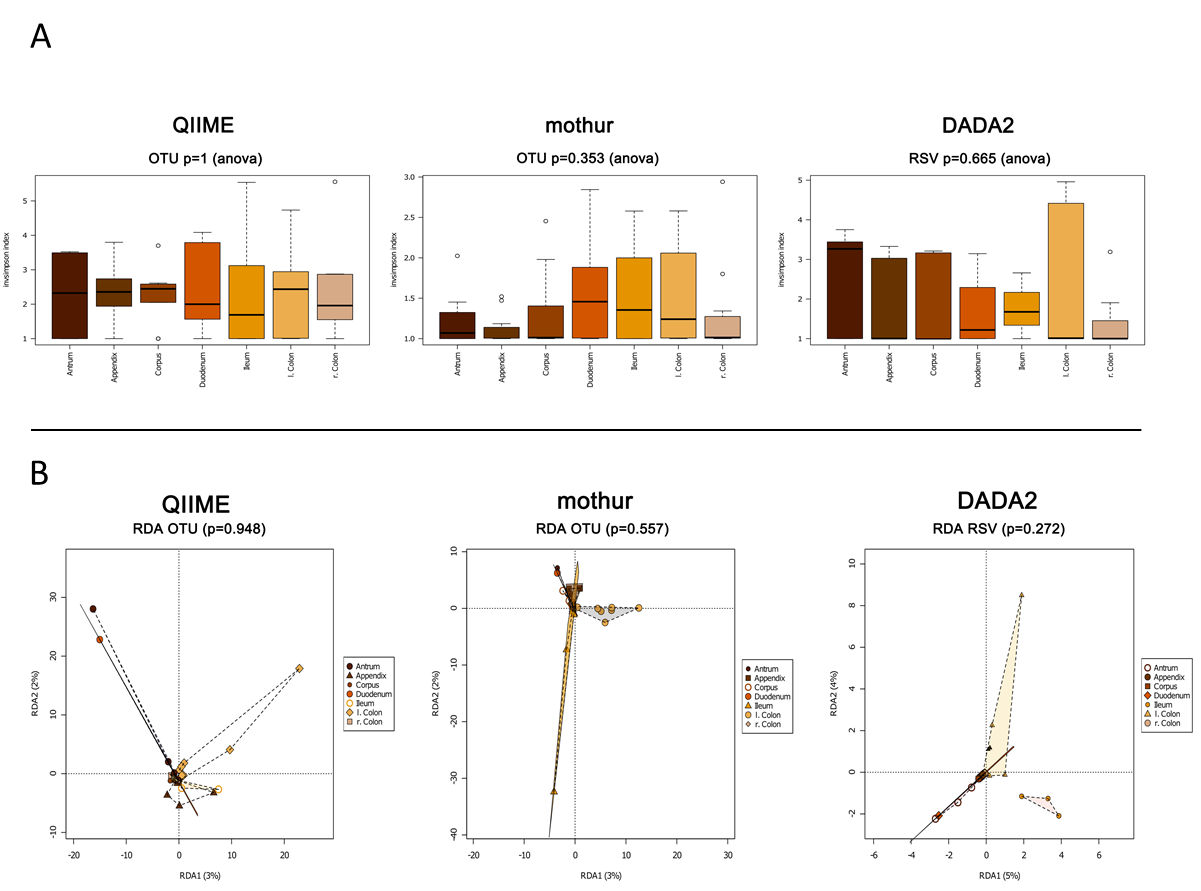

Supplement: FIG S3 [file mbo005173581sf3.tif]

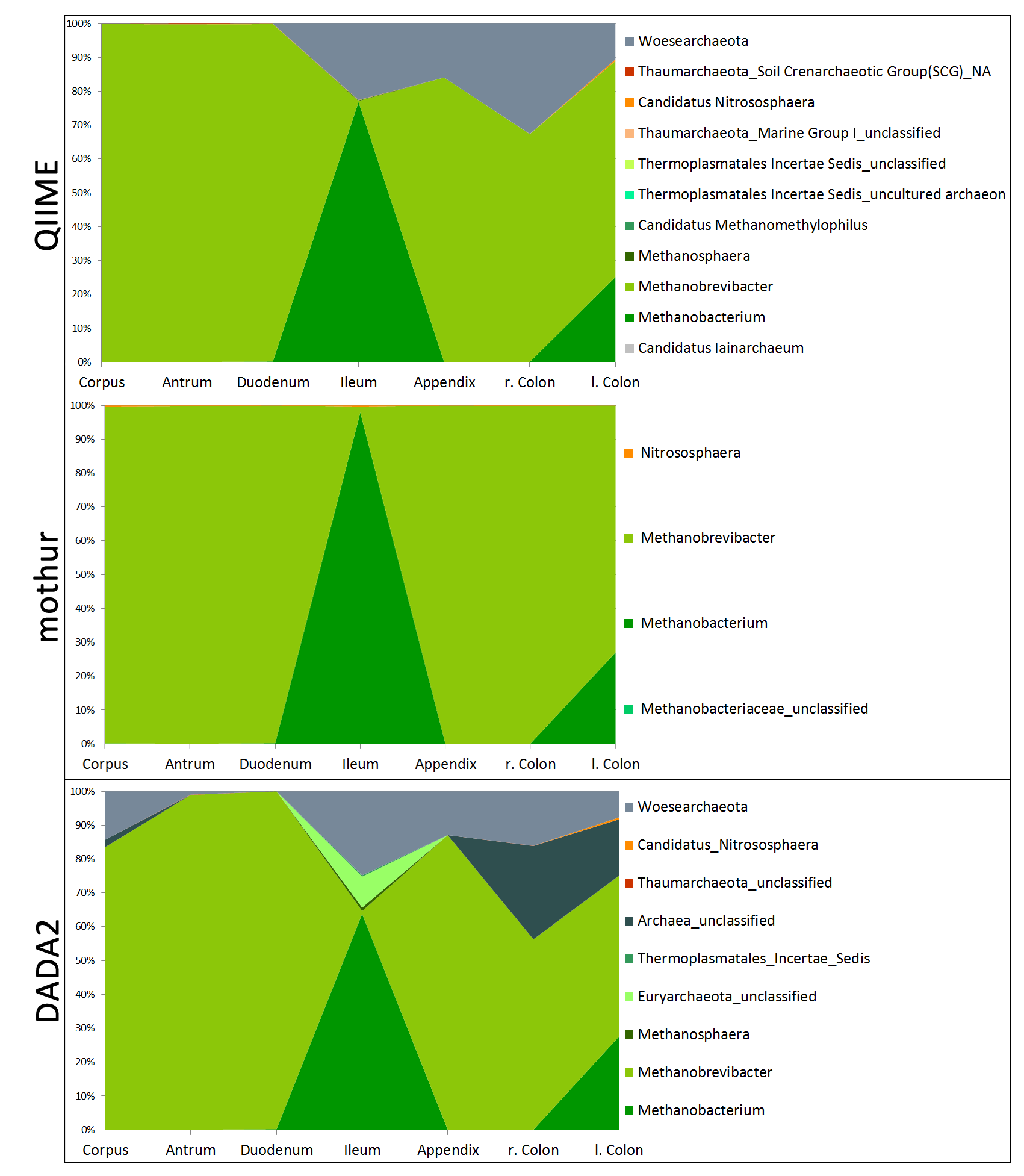

Supplement: FIG S4 [file mbo005173581sf4.tif]

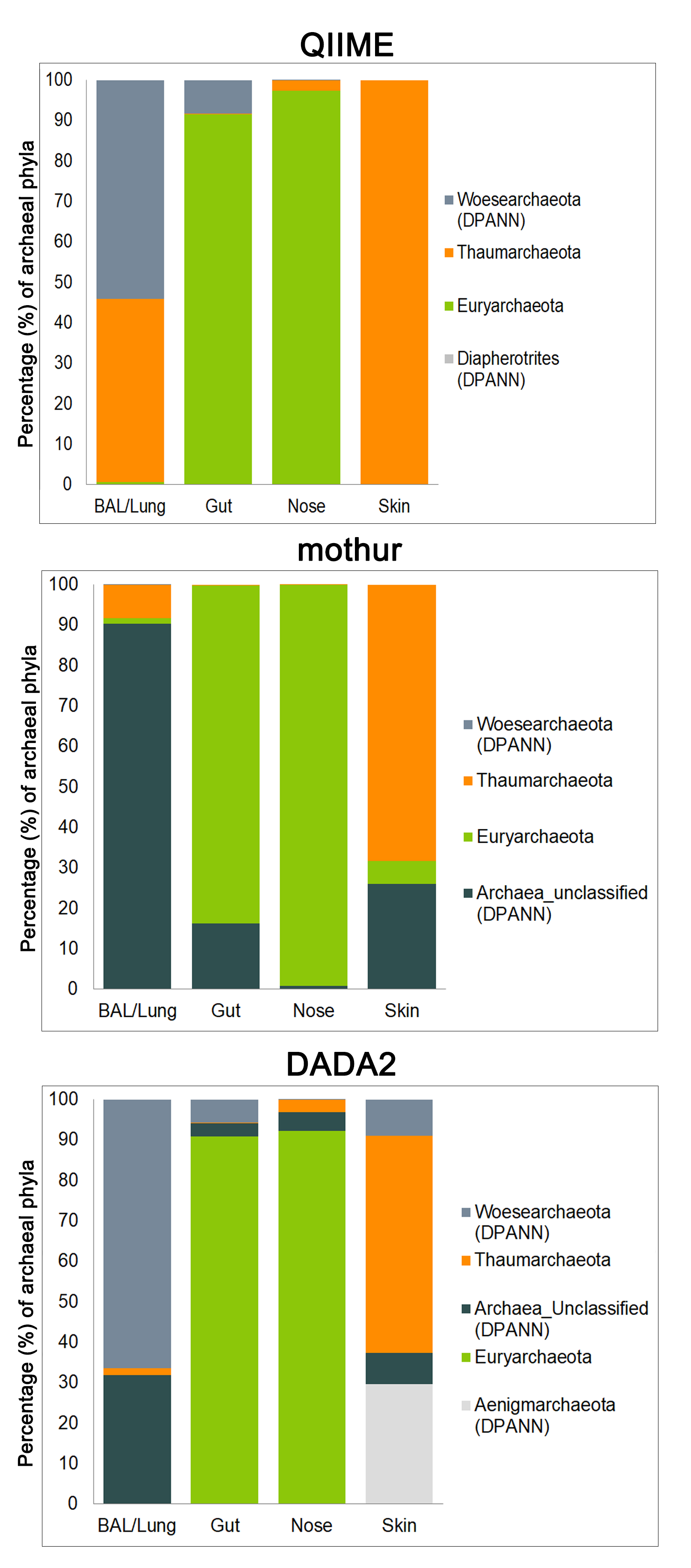

Supplement: FIG S5 [file mbo005173581sf5.tif]

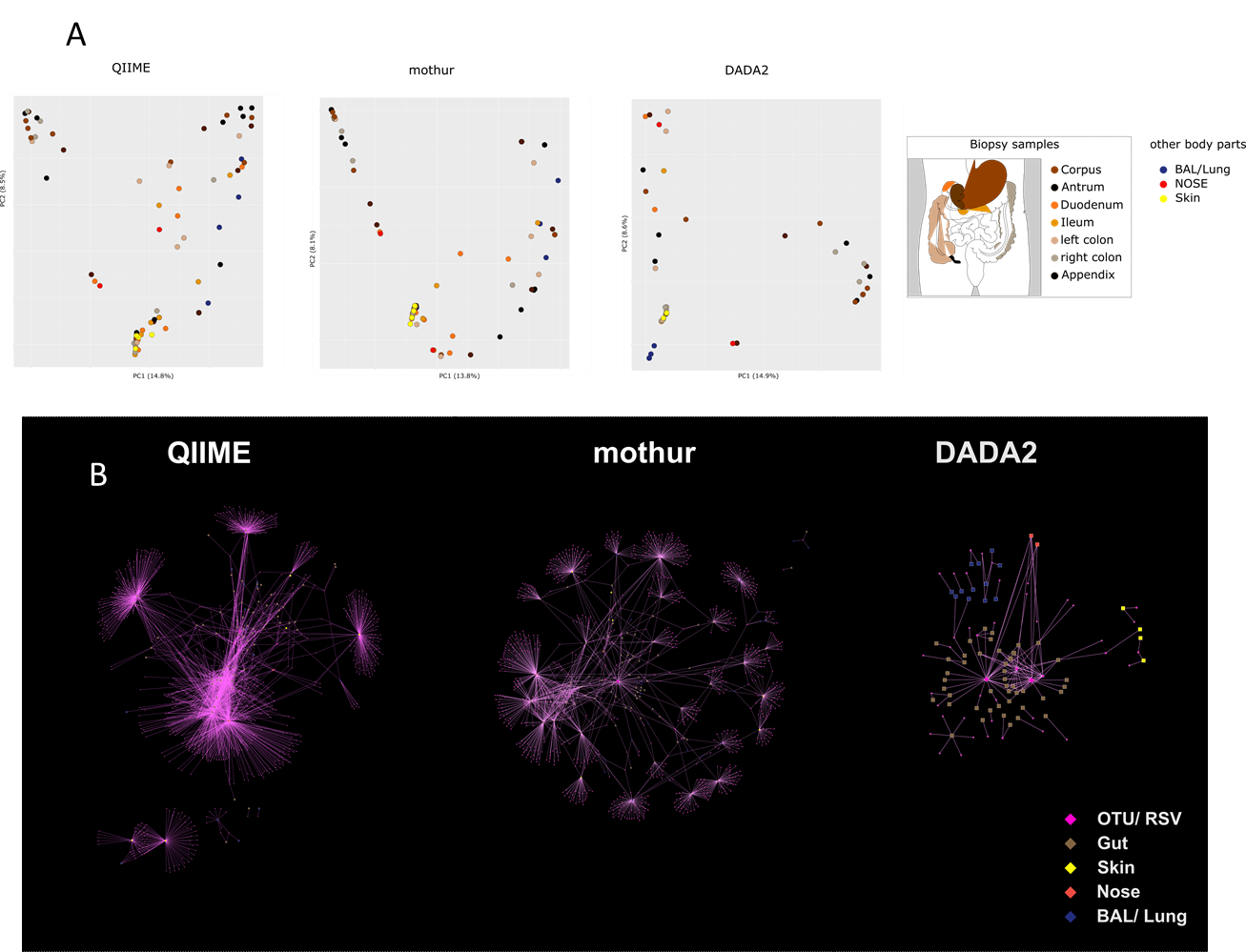

Supplement: FIG S6 [file mbo005173581sf6.tif]
